# An efficient synthesis of N-substituted 3-nitrothiophen-2-amines

**DOI:** 10.3762/bjoc.11.185

**Published:** 2015-09-22

**Authors:** Sundaravel Vivek Kumar, Shanmugam Muthusubramanian, J Carlos Menéndez, Subbu Perumal

**Affiliations:** 1 Department of Organic Chemistry, School of Chemistry, Madurai Kamaraj University,Madurai – 625021, Tamil Nadu, India; 2 Departmento de Química Orgánica y Farmacéutica, Facultad de Farmacia, Universidad Complutense, 28040 Madrid, Spain

**Keywords:** C–C bond generation, nitroarenes, nitroketene acetals, sulfur heterocycles

## Abstract

A novel protocol for the synthesis of 3-nitro-*N*-aryl/alkylthiophen-2-amines in good yields from the reaction of α-nitroketene *N*,*S*-aryl/alkylaminoacetals and 1,4-dithiane-2,5-diol in the presence of K_2_CO_3_ in refluxing ethanol is described. This transformation generates two C–C bonds in a single operation and presumably proceeds through a reaction sequence comprising 2-mercaptoacetaldehyde generation, nucleophilic carbonyl addition, annelation and elimination steps.

## Introduction

**Figure 1:**
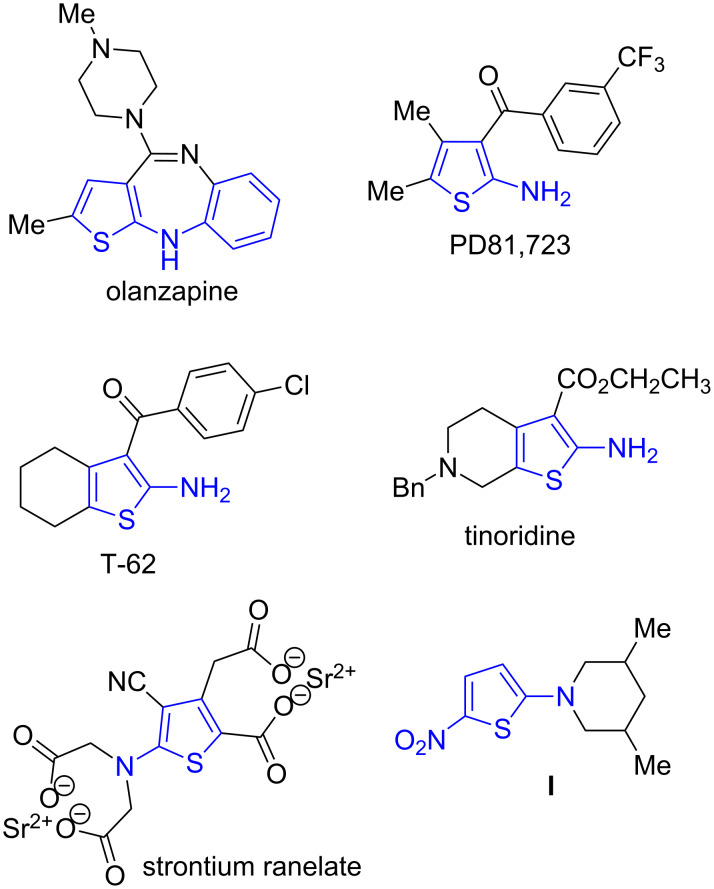
Selected examples of biologically active 2-aminothiophene derivatives.

Thiophenes constitute an important class of heterocyclic compounds, and are embedded in natural [1] and unnatural [2] organic molecules with diverse biological activities. Among them, many 2-aminothiophenes have reached the market or have undergone extensive pharmacological studies. Representative examples are the neuroleptic olanzapine [3], the 2A3BT family of allosteric modulators of the A1 adenosine receptor, derived from 2-amino-3-benzoylthiophene and compounds such as PD-81,723 and T-62 [4], the non-steroidal anti-inflammatory tinoridine [5] and ranelic acid, whose strontium salt is used as a medication for osteoporosis [6] (Figure 1). Other interesting properties identified in 2-aminothiophene derivatives include GluR6 antagonism [7], antiviral and antitumor activities [8–10], inhibition of p53-MDM2 interactions [11] and protein-tyrosine phosphatase 1B inhibition [12]. Furthermore, 2-aminothiophenes have potential applications in the field of functional materials such as liquid crystals [13], nonlinear optical materials (NLOs) [14], organic solar cells [15], photonic polymers [16] and electroluminescent materials [17]. Nitrothiophenes are also important as chemotherapeutic agents and in a few cases both functional groups are combined in the same molecule. For instance, compound **I** (Figure 1) is an antitubercular agent that acts by releasing nitric oxide following activation by F420-dependent nitroreductase DDN [18]. However, the pharmacological study of this class of compounds is hampered by limitations in existing synthetic methodologies, particularly in the direct preparation of N-substituted amino derivatives.

**Scheme 1:**
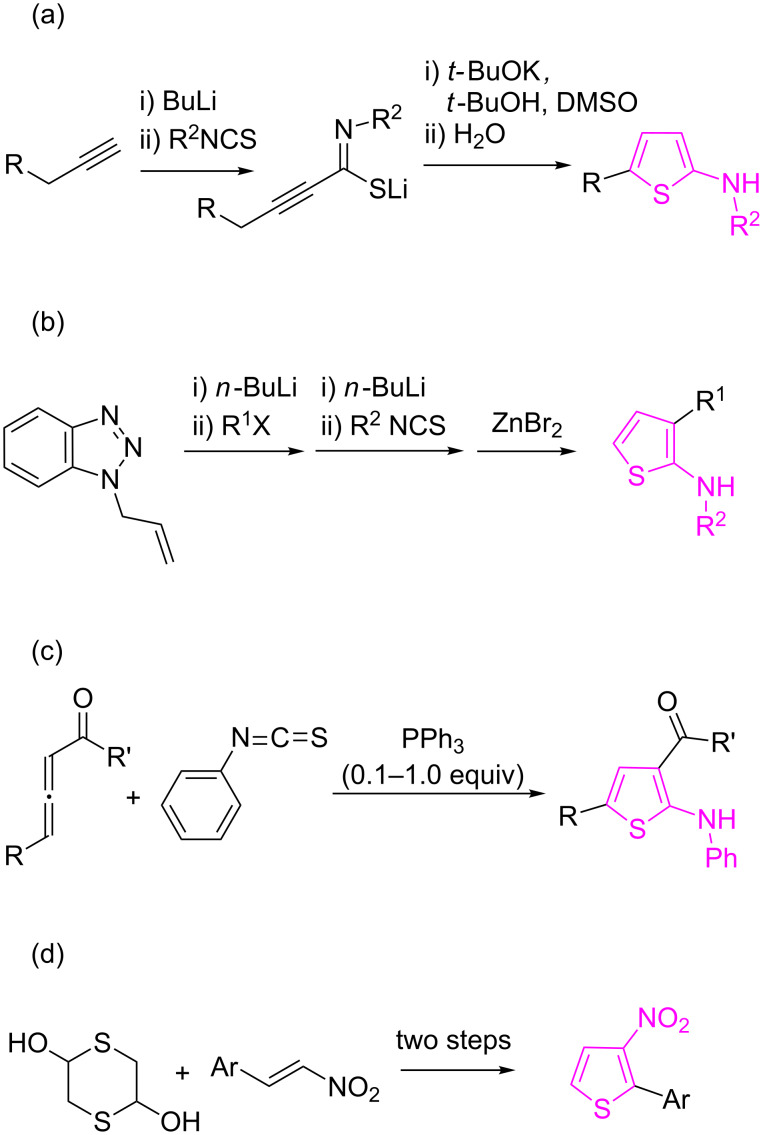
Some strategies for the synthesis of 2-aryl/alkylaminothiophenes and 3-nitrothiophenes.

2-Aminothiophenes have traditionally been synthesized from mercapto- or halogen-substituted thiophenes through nucleophilic displacements [19]. More recently, these derivatives have been assembled from acyclic starting materials by methods such as i) reaction of lithiated 1-alkynes or allenes with substituted phenyl isothiocyanates [20], ii) treatment of *N*-allylbenzotriazole with substituted phenylisothiocyanates [21] and iii) reaction between phenyl isothiocyanate and electron-deficient allenes catalyzed by PPh_3_ [22] (Scheme 1a–c). On the other hand, studies on the synthesis of 3-nitrothiophenes are scarce. One of the traditional methods involves electrophilic aromatic substitution reactions of thiophenes, which introduces substituents at the 2- and 5-positions, but with some drawbacks associated with the lack of regioselectivity and difficult isomer separation [23]. Other methods for the synthesis of 2,3-disubsituted thiophenes include the reactions of (i) 1,4-dithiane-2,5-diol with nitroalkenes [24], activated nitriles [25] and cyanoacetamide [26]; (ii) 3-nitrothiophene with Grignard reagents [27] and (iii) 3-bromo-2-silylthiophene with aldehydes [28]. The major drawback of these methods is that none of them allow the simultaneous introduction of 2-amino and 3-nitro substituents (see Scheme 1d for a representative example).

**Scheme 2:**
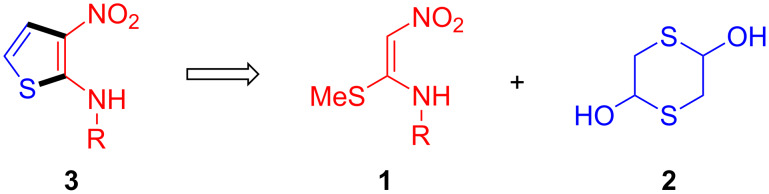
Our plan for the synthesis of N-substituted 3-nitrothiophen-2-amines.

Multiple bond-forming transformations [29–31] are very attractive because of the high synthetic efficiency associated to the formation of several bonds in a single operation. The corresponding reduction in the number of isolation and purification steps and hence in the use of organic solvents and chromatographic stationary phases helps to achieve one of the main goals of green chemistry [32]. In this context, we wish to report herein a new and efficient synthesis of 3-nitro-*N*-aryl/alkylthiophen-2-amines based on domino reactions between α-nitroketene *N*,*S*-aryl/alkylaminoacetals and 1,4-dithiane-2,5-diol, the dimer of 2-mercaptoacetaldehyde (Scheme 2). This work emerges as a continuation of our recent efforts directed towards the construction of biologically relevant heterocycles employing multiple bond-forming transformations [33–40].

## Results and Discussion

In a preparatory study, the optimization of the reaction between (*E*)-4-methyl-*N*-(1-(methylthio)-2-nitrovinyl)aniline (**1e**, 1 mmol) and 1,4-dithiane-2,5-diol (**2**, 0.5 mmol, 1 equiv) was performed. Initially, this model reaction was attempted in ethanol and in the absence of base, with negative results (Table 1, entry 1). In the presence of TEA (1 equiv), it gave 62% yield (Table 1, entry 2), which was increased to 88% when the reaction was performed in refluxing ethanol for 25 min (Table 1, entry 3). In both cases, work-up had the advantage of being very simple. After completion of the reaction, the mixture was poured into water and the precipitated solid was ﬁltered and washed with ethanol to give the pure 3-nitro-*N*-(*p*-tolyl)thiophen-2-amine (**3e**) without the need for chromatography. Then the model reaction was further investigated by employing alternative bases such as 1,4-diazabicyclo[2.2.2]octane (DABCO, Table 1, entry 4), 1,8-diazabicyclo[5.4.0]undec-7-ene (DBU, Table 1, entry 5), pyridine (Table 1, entry 6), *N*,*N-*dimethylaminopyridine (DMAP, Table 1, entry 7), piperidine (Table 1, entry 8), L-proline (Table 1, entry 9), potassium carbonate (Table 1, entry 10), sodium carbonate (Table 1, entry 11) and caesium carbonate (Table 1, entry 12). After identifying potassium carbonate as the optimal base in refluxing ethanol (93% yield), alternative solvents such as methanol (Table 1, entry 13), *N*,*N*-dimethylformamide (Table 1, entry 14), acetonitrile (Table 1, entry 15), tetrahydrofuran (Table 1, entry 16), 1,4-dioxane (Table 1, entry 17) and water (Table 1, entry 18) were examined, without affording additional improvements. Finally, the use of substoichiometric amounts of base was examined (Table 1, entries 19–21), leading to the conclusion that 0.25 equivalents of potassium carbonate were sufficient to promote the desired transformation, but lower amounts led to longer reaction times and diminished yields (Table 1, entry 22). All reactions included in Table 1 were performed under heating at reflux temperature of the respective solvents used, except reactions corresponding to entries 1 and 2, which were performed at room temperature.

**Table 1:** Optimization of the synthesis of compound **3e**^a^.

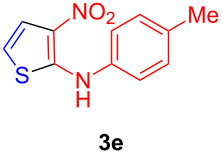				

Entry	Base (1 equiv)	Solvent	Time, min	Yield, %^b^

1	–	EtOH	60	0^b,c^
2	TEA	EtOH	180	62^b^
3	TEA	EtOH	25	88
4	DABCO	EtOH	25	76
5	DBU	EtOH	25	64
6	pyridine	EtOH	30	0
7	DMAP	EtOH	25	44
8	piperidine	EtOH	30	0
9	L-proline	EtOH	30	0
10	K_2_CO_3_	EtOH	25	93
11	Na_2_CO_3_	EtOH	25	83
12	Cs_2_CO_3_	EtOH	25	90
13	K_2_CO_3_	MeOH	25	88
14	K_2_CO_3_	DMF	25	86
15	K_2_CO_3_	CH_3_CN	30	53
16	K_2_CO_3_	THF	30	trace
17	K_2_CO_3_	1,4-dioxane	30	22
18	K_2_CO_3_	water	30	48
19	K_2_CO_3_^d^	EtOH	25	94
20	K_2_CO_3_^e^	EtOH	25	93
21	K_2_CO_3_^f^	EtOH	25	94
22	K_2_CO_3_^g^	EtOH	30	88

^a^All reactions were performed with a mixture of **1e** (1 mmol) and **2** (0.5 mmol). ^b^Isolated yield after washing with cold ethanol. ^c^Reaction performed at room temperature. ^d^75 mol %. ^e^50 mol %. ^f^25 mol %. ^g^20 mol %.

**Scheme 3:**
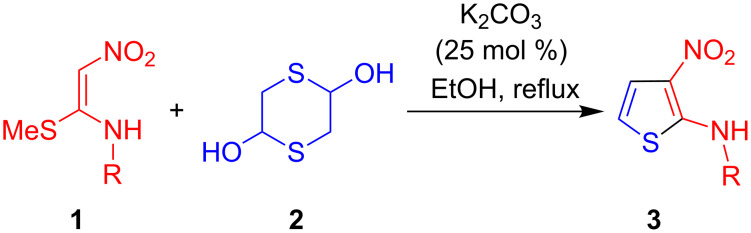
Synthesis of N-substituted 3-nitrothiophen-2-amines.

The scope and generality of this transformation was investigated by examining the reaction between a variety of α-nitroketene *N*,*S*-anilinoacetals **1** with 1,4-dithiane-2,5-diol (**2**, Scheme 3 and Table 2). The starting materials **1** were readily available by refluxing in ethanol the suitable primary amine and 1,1-bis(methylthio)-2-nitroethylene. Regarding aryl substituents, the thiophene synthesis was compatible with the presence of unsubstituted phenyl rings (compound **3e**) and phenyl substituents bearing moderately electron-withdrawing groups such as *m*- and *p*-halogens and *m*-trifluoroalkyl substituents (compounds **3a**–**d** and **3l**–**n**), although substrates **1** with aryl rings bearing strongly electron-withdrawing groups such as *p*-nitro, *p*-cyano and *p*-trifluoromethyl failed to afford the final products. Furthermore, it was gratifying to observe that our method was compatible with the presence of electron-releasing substituents at the aromatic ring, including alkyl (compounds **3f**–**h**, **3j**, **3o** and **3q**) and alkoxy (compounds **3i**, **3k** and **3p**) substituents, even at the more hindered *ortho*-positions (**3j**, **3k**, **3q**). The preparation of a compound bearing a 1-naphthylamino substituent (compound **3r**) also proceeded uneventfully. We also investigated the reactions of α-nitroketene *N*,*S*-alkylaminoacetals and 1,4-dithiane-2,5-diol (**2**). These reactions provided the desired 2-(alkylamino)-3-nitrothiophenes in excellent yields, albeit with the need for longer reaction times. The method allowed the preparation of compounds bearing linear (compounds **3s**–**u**) and α-branched alkyl substituents (compounds **3v**, **3x**). Benzyl substituents could also be accommodated, as shown by the efficient preparation of **3y** and **3z**.

**Table 2:** Scope of the 2-amino-3-nitrothiophene synthesis.

Compound	R	Time, min	Yield, %

**3a**	4-FC_6_H_4_	25	90
**3b**	4-ClC_6_H_4_	25	92
**3c**	4-BrC_6_H_4_	25	91
**3d**	4-IC_6_H_4_	25	93
**3e**	C_6_H_5_	24	94
**3f**	4-MeC_6_H_4_	23	94
**3g**	4-EtC_6_H_4_	23	95
**3h**	4-iPrC_6_H_4_	22	93
**3i**	4-MeOC_6_H_4_	20	96
**3j**	2-MeC_6_H_4_	22	93
**3k**	2-MeOC_6_H_4_	25	91
**3l**	3-FC_6_H_4_	24	94
**3m**	3-BrC_6_H_4_	24	90
**3n**	3-F_3_CC_6_H_4_	25	89
**3o**	3-MeC_6_H_4_	24	94
**3p**	3-MeOC_6_H_4_	22	95
**3q**	2,4-Me_2_C_6_H_3_	23	82
**3r**	1-naphthyl	25	91
**3s**	Me	205	93
**3t**	*n*-Pr	190	95
**3u**	*n*-Bu	190	98
**3v**	iPr	205	93
**3w**	cyclopropyl	190	94
**3x**	cyclohexyl	190	95
**3y**	Bn	180	92
**3z**	(*R*)-α-MeBn	180	96

**Figure 2:**
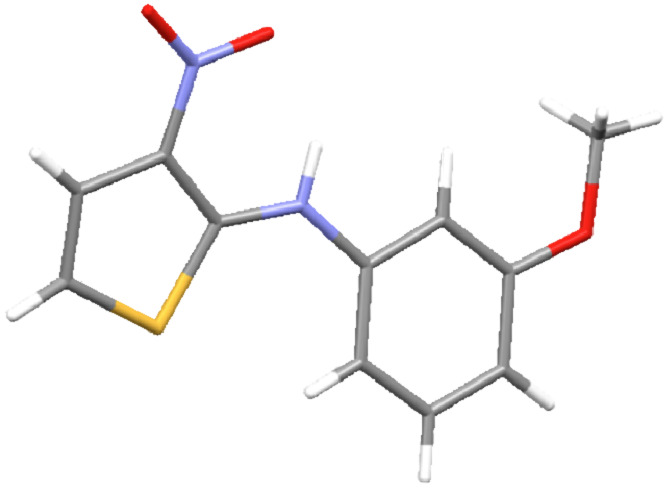
X-ray diffraction study of compound **3p**.

The structure of the 3-nitro-*N*-arylthiophen-2-amines **3** was deduced from one- and two-dimensional NMR spectroscopic, microanalytical and mass spectral data, as detailed for **3c** as a representative example (see Supporting Information File 1) and conﬁrmed unambiguously by a single-crystal X-ray crystallographic study [41] of **3p** (Figure 2).

**Scheme 4:**
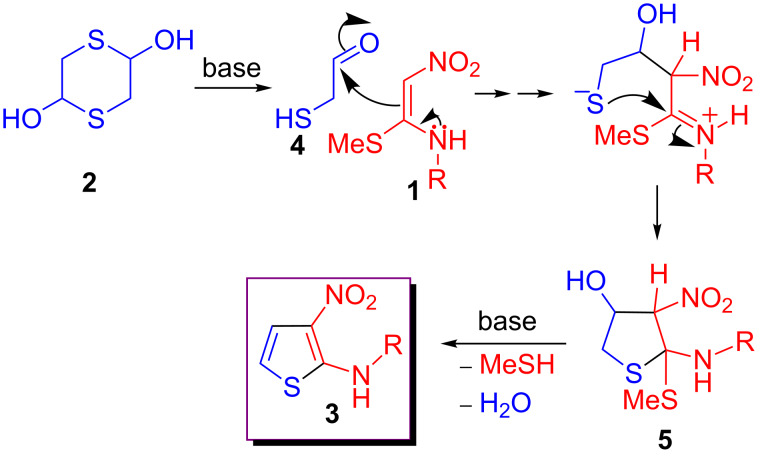
Proposed reaction sequence leading to the formation of **3**.

The transformation leading to compounds **3** presumably proceeds through the initial formation of 2-mercaptoacetaldehyde (**4**) from the base-promoted decomposition of 1,4-dithiane-2,5-diol (**2**). This would be followed by a formal [3 + 2] annexation via nucleophilic addition of compounds **1** to the carbonyl of intermediate **4** followed by addition of the thiolate anion onto the iminium functionality thus generated to give **5**. Subsequent elimination of methylmercaptan and water furnishes the product **3** (Scheme 4).

## Conclusion

In conclusion, we have developed a very efficient synthesis of a synthetically and biologically relevant class of push–pull heterocyclic compounds under mild conditions and starting from simple acyclic substrates and catalysts. Previously, these compounds were available only through multistep sequences, most notably one involving as the final step an S_N_Ar reaction with the amine and having the serious limitation of requiring the presence of an additional strong electron-withdrawing group at C-5 [42–44]. Our protocol requires only two steps (including the preparation of starting materials **1**) and generates two C–C bonds through a sequence of reactions that comprise up to five individual steps and proceeds in excellent yields (93% in average for 26 examples) and atom economies.

## Supporting Information

File 1 Experimental procedures, characterization data, details of the NMR structural determination of **3c** and copies of ^1^H NMR, ^13^C NMR and ESI mass spectra of all new compounds **1** and **3**.
