# Supplementary material for: An efficient synthesis of N-substituted 3-nitrothiophen-2-amines
Source: Beilstein J Org Chem. 2015 Sep 22;11:1707–12. doi: 10.3762/bjoc.11.185 (PMC4660920; doi:10.3762/bjoc.11.185)
Supplement: File 1 — Experimental procedures, characterization data, details of the NMR structural determination of 3c and copies of 1H NMR, 13C NMR and ESI mass spectra of all new compounds 1 and 3. [file Beilstein_J_Org_Chem-11-1707-s001.pdf]

Supporting Information

for

An efficient synthesis of N-substituted 3-nitrothiophen-2-amines

Sundaravel Vivek Kumar[1], Shanmugam Muthusubramanian[1], J. Carlos Menéndez[2*] and Subbu Perumal[1*]

Address: [1]Department of Organic Chemistry, School of Chemistry, Madurai Kamaraj University,Madurai – 625021, Tamil Nadu, India and [2]Departmento de Química Orgánica y Farmacéutica, Facultad de Farmacia, Universidad Complutense, 28040 Madrid, Spain

Email: J. Carlos Menéndez - josecm@farm.ucm.es; Subbu Perumal - subbu.perum@gmail.com

* Corresponding author

Experimental procedures, characterization data, details of the NMR structural determination of **3c** and copies of ^{1}H NMR, ^{13}C NMR and ESI mass spectra of all new compounds **1** and **3**

1. Experimental Section

General experimental information. Melting points were measured in open capillary tubes and are uncorrected. Infrared spectra were obtained on a Shimadzu 8400S FT-IR Spectrophotometer, in a 4000-400 cm^{-1} spectral window, using neat samples on a KBr disk or KBr pellets. ^{1}H NMR, ^{13}C NMR, DEPT, H,H-COSY, C,H-COSY and HMBC spectra were recorded on a Bruker (Avance) 300 MHz NMR instrument using TMS as internal standard and $CDCl_3$ as solvent. Standard Bruker software was used throughout. Chemical shifts are given in parts per million (δ scale) and the coupling constants are given in Hertz (Hz). Mass spectra were recorded with a LCQ Fleet mass spectrometer, Thermo Fisher Instruments Limited, US. Electrospray ionization mass spectrometry (ESIMS) analysis was performed in the positive/negative ion mode on a liquid chromatography ion trap. Combustion microanalyses were performed by the CAI de Microanálisis Elemental, Universidad Complutense, using a Leco-932 CHNS microanalyzer. Silica gel-G plates (Merck) were used for TLC analysis with mixtures of petroleum ether (bp 60–80 °C) and ethyl acetate as eluent.

General procedure for the synthesis of α-nitroketene N,S-arylaminoacetals 1a–1r [1]. A mixture of 1,1-bis(methylthio)-2-nitroethylene (1 mmol) and the suitable aromatic amine in ethanol was magnetically stirred while heated under reflux for 24 h. After completion of the reaction (TLC), the mixture was cooled to room temperature, and the separated solid was filtered and washed with ethanol to afford the pure *α*-nitroketene *N,S*-arylaminoacetals.

General procedure for the synthesis of α-nitroketene N,S-alkylaminoacetals 1s–1z [2]. A mixture of 1,1-bis(methylthio)-2-nitroethylene (1 mmol) and the

suitable aliphatic amine in ethanol was magnetically stirred while heated under reflux for 24 h. After completion of the reaction (TLC), the solvent was removed under reduced pressure and the resulting crude products were purified by flash chromatography eluting with $CHCl_3$/MeOH mixtures to afford the pure α-nitroketene N,S-alkylaminoacetals.

Compounds **1d**, **1g**, **1m**, **1q**, **1w** and **1z** are new and their characterization data follow.

(*E*)-4-Iodo-*N*-(1-(methylthio)-2-nitrovinyl)aniline (1d): Isolated as a pale green solid. Yield: 85%; mp = 174–175 °C; IR (KBr) ν_{max}: 3145, 3003, 2954, 2926, 2853,1544, 1460, 1344, 1265, 1168 cm $^{-1}$; H NMR (300 MHz, $CDCl_3$) δ_H: 2.40 (s, 3H, SCH_3),6.69 (s, 1H, CH), 7.06 (d, *J* = 8.4 Hz, 2H, Ar-H), 7.75 (d, *J* = 8.7 Hz, 2H, Ar-H), 11.72 (br s, 1H, NH); ^{13}C NMR (75 MHz, $CDCl_3$) δ_c: 14.7, 93.5, 108.2, 127.6, 135.9, 138.5, 162.8; ESI-MS: m/z. Calcd: 335.94. Found: 334.90 (M-1).

(*E*)-4-Ethyl-*N*-(1-(methylthio)-2-nitrovinyl)aniline (1g): Isolated as a yellow solid. Yield: 88%; mp = 111–112 °C; IR (KBr) ν_{max}: 3157, 2997, 2962, 2929, 1560, 1425, 1346, 1265, 1166 cm $^{-1}$; H NMR (300 MHz, $CDCl_3$) δ_H: 2.38 (s, 3H, SCH_3), 6.70 (s, 1H, CH), 7.20 (d, *J* = 8.4 Hz, 2H, Ar-H), 7.25 (d, *J* = 6.9 Hz, 2H, Ar-H), 11.78 (br s, 1H, NH); ^{13}C NMR (75 MHz, $CDCl_3$) δ_c: 14.6, 15.2, 28.3, 107.4, 125.8, 128.7, 133.6, 144.5, 163.9; ESI-MS: m/z. Calcd: 238.08. Found: 239.01 (M+1).

(*E*)-3-Bromo-*N*-(1-(methylthio)-2-nitrovinyl)aniline (1m): Isolated as a pale yellow solid. Yield: 83%; mp = 118–119 °C; IR (KBr) ν_{max}: 3152, 3001, 2929, 1547, 1460, 1352, 1247, 1174 cm $^{-1}$; H NMR (300 MHz, $CDCl_3$) δ_H: 2.40 (s, 3H, SCH_3), 6.69 (s, 1H, CH), 7.24-7.33 (m, 2H, Ar-H), 7.48-7.51 (m, 2H, Ar-H) 11.74 (br s, 1H, NH); ^{13}C NMR (75

MHz, $CDCl_3$) δ_c: 14.7, 108.3, 122.7, 124.6, 128.9, 130.6, 131.1, 137.4, 162.9; ESI-MS: m/z. Calcd: 287.96. Found: 286.94 (M-1), 288.91(M+1).

(*E*)-2,4-Dimethyl-*N*-(1-(methylthio)-2-nitrovinyl)aniline (1q): Isolated as a yellow solid. Yield: 87%; mp = 132–133 °C; IR (KBr) ν_{max}: 3146, 2995, 2926, 1544,1460, 1325, 1267, 1178 cm $^{-1}$; H NMR (300 MHz, $CDCl_3$) δ_H: 2.26 (s, 3H, SCH_3), 2.35 (s, 6H, Ar-$(CH_3)_2$) 6.70 (s, 1H, CH), 7.05 (d, J = 7.8 Hz, 1H, Ar-H), 7.11-7.13 (m, 2H, Ar-H) 11.53 (br s, 1H, NH); ^{13}C NMR (75 MHz, $CDCl_3$) δ_c: 14.4, 17.7, 21.1, 107.1, 127.2, 127.4, 131.8, 132.3, 134.8, 139.0, 165.0; ESI-MS: m/z. Calcd: 238.08. Found: 239.02 (M+1).

(*E*)-*N*-(1-(Methylthio)-2-nitrovinyl)cyclopropanamine (1w): Isolated as a pale yellow solid. Yield: 93%; mp = 85–86 °C; IR (Neat) ν_{max}: 3199, 3169, 3088, 2991, 1564, 1467, 1340, 1230, 1165, 1047 cm $^{-1}$; H NMR (300 MHz, $CDCl_3$) δ_H: 0.81-0.98 (m, 4H, CH_2-CH_2), 2.42 (s, 3H, SCH_3), 2.70-2.73 (m, 1H, CH) 6.55 (s, 1H, CH), 10.34 (br s, 1H, NH); ^{13}C NMR (75 MHz, $CDCl_3$) δ_c: 8.3, 14.2, 25.7, 106.1, 167.3; ESI-MS: m/z. Calcd: 174.05. Found: 175.01 (M+1).

(*R,E*)-1-(Methylthio)-2-nitro-*N*-(1-phenylethyl)ethen-1-amine (1z): Isolated as a yellow solid. Yield: 90%; mp = 41–42 °C; IR (KBr) ν_{max}: 3146, 3022, 2978, 2928, 1557,1464, 1415, 1325, 1215, 1118, 1080 cm $^{-1}$; H NMR (300 MHz, $CDCl_3$) δ_H: 1.65 (d, *J* = 6.6 Hz, 3H, CH_3), 2.36 (s, 3H, SCH_3), 4.89-4.96 (m, 1H, CH), 6.56 (s, 1H, CH), 7.26-7.40 (m, 5H, Ar-H) 10.90 (br s, 1H, NH); ^{13}C NMR (75 MHz, $CDCl_3$) δ_c: 14.6, 24.0, 54.7, 106.6, 125.8, 128.0, 129.0, 141.6, 164.0; ESI-MS: m/z. Calcd: 238.08. Found: 237.02 (M-1).

General procedure for the synthesis of 3-nitro-N-arylthiophen-2-amines 3a–r. A mixture of the suitable α-nitroketene N,S-arylaminoacetal (1 mmol), 1,4-dithiane-2,5-diol (0.5 mmol) and K_2CO_3 (25 mol %) in ethanol (6 mL) was heated under reflux for 20–25 min. After completion of the reaction (TLC), the mixture was cooled to room temperature, and the precipitated solid was filtered and washed with ethanol to afford the pure 3-nitro-N-arylthiophen-2-amines **3a–r**.

General procedure for the synthesis of 3-nitro-*N*-alkylthiophen-2-amines 3s–3z. A mixture of the suitable α-nitroketene N,S-alkylaminoacetal (1 mmol), 1,4-dithiane-2,5-diol (0.5 mmol) and K_2CO_3 (25 mol %) in ethanol (6 mL) was heated under reflux for 3–3.4 h. After completion of the reaction (TLC), the mixture was poured into water and extracted with ethyl acetate. After removal of the solvent, the residue was purified by filtration through a pad of silica gel, eluting with a petroleum ether/ethyl acetate mixture (4:1 v/v), which afforded 3-nitro-N-alkylthiophen-2-amines **3s**–**3z**.

***N*-(4-Fluorophenyl)-3-nitrothiophen-2-amine (3a)**. Isolated as a pale yellow solid. Yield: 90%; mp = 129–130 °C; IR (KBr) ν_{max}: 3261,1560,1508, 1490, 1342, 1226, 1192 cm^{-1}. ^1H NMR (300 MHz, $CDCl_3$) δ_H: 6.29 (d, J = 6.3 Hz, 1H, H-5), 7.12–7.18 (m, 2H, H-3’,5’), 7.34–7.39 (m, 3H, H-4, H-2’,6’), 10.16 (br s, 1H, NH); ^{13}C NMR (75 MHz, $CDCl_3$) δ_c: 107.0 (C-4), 116.8 (d, $^2J_{C,F}$ = 22.9 Hz, C-3’,5’), 121.9 (C-5), 123.4 (d, $^3J_{C,F}$ = 8.3 Hz, C-2’,6’), 127.9 (C-1’), 135.2 (C-1), 156.8 (C-3), 160.5 (d, $^1J_{C,F}$ = 245.5 Hz, C4’); ESI-MS: m/z. Calcd: 238.02. Found: 237.08 (M-1). Analysis: Calcd for $C_{10}H_7FN_2O_2S$: C, 50.41; H, 2.96; N, 11.76. Found: C, 49.91; H, H, 2.98; N, 11.60.

***N*-(4-Chlorophenyl)-3-nitrothiophen-2-amine (3b)**. Isolated as a pale yellow solid. Yield: 92%; mp = 161–162 °C. IR (KBr) ν_{max}: 3259, 3116, 3099, 1560, 1508, 1491,

1342, 1261, 1203,1091 cm^{-1}. ^{1}H NMR (300 MHz, $CDCl_3$) δ_H: 6.33 (d, *J* = 6.0 Hz, 1H, H-5), 7.31–7.43 (m, 5H, H-4, H-2',3',5',6'), 10.29 (br s, 1H, NH); ^{13}C NMR (75 MHz, $CDCl_3$) δ_c: 107.4 (C-4), 121.8 , 121.9 (C-2',6'), 128.4 (C-4'), 130.0 (C-3',5'), 131.1 (C-5), 137.6 (C-1'), 155.2 (C-3); ESI-MS: m/z. Calcd: 253.99. Found: 253.00 (M-1), 255.12 (M+2). Analysis: Calcd for $C_{10}H_7ClN_2O_2S$: C, 47.16; H, 2.77; N, 11.00. Found: C, 46.87; H, 2.88; N, 10.80.

***N*-(4-Bromophenyl)-3-nitrothiophen-2-amine (3c)**. Isolated as a pale yellow solid. Yield: 91%; mp = 173–174 °C. IR (KBr) ν_{max}: 3253, 3116, 3099, 1560,1508, 1483, 1340, 1199, 1085 cm^{-1}. ^{1}H NMR (300 MHz, $CDCl_3$) δ_H: 6.34 (d, *J* = 6.3 Hz, 1H, H-5), 7.26 (d, *J* = 8.7 Hz, 2H, H-3',5'), 7.37 (d, *J* = 6.0 Hz, 1H, H-4), 7.56 (d, *J* = 8.7 Hz, 2H, H-2',6'), 10.30 (br s, 1H, NH); ^{13}C NMR (75 MHz, $CDCl_3$) δ_c: 107.4 (C-4), 118.6 (C-2',6'), 121.9 (C-5), 122.0 (C-3',5'), 128.5 (C-4'), 132.9 (C-1'), 138.1 (C-2), 154.9 (C-3); ESI-MS: m/z. Calcd: 297.94. Found: 297.06 (M-1), 299.06 (M+2). Analysis: Calcd for $C_{10}H_7BrN_2O_2S$: C, 40.15; H, 2.36; N, 9.36. Found: C, 39.93; H, 2.88; N, 10.80.

***N*-(4-Iodophenyl)-3-nitrothiophen-2-amine (3d)**. Isolated as a yellow solid. Yield: 93%; mp = 185–186 °C. IR (KBr) ν_{max}: 3209, 3120, 3105, 1560, 1350, 1240, 1195 cm^{-1}. ^{1}H NMR (300 MHz, $CDCl_3$) δ_H: 6.35 (d, *J* = 6.3 Hz, 1H, H-5), 7.15 (d, *J* = 8.7 Hz, 2H, H-2',6'), 7.37 (d, *J* = 6.0 Hz, 1H, H-4), 7.75 (d, *J* = 8.7 Hz, 2H, H-3',5'), 10.32 (br s, 1H, NH); ^{13}C NMR (75 MHz, DMSO-d^6) δ_c: 90.7 (C-4'), 109.5 (C-4), 121.5 (C-5), 124.7 (C-3',5'), 127.9 (C-1'), 138.6 (C-2',6'), 140.1 (C-2), 156.5 (C-3); ESI-MS: m/z. Calcd: 345.93. Found: 344.91 (M-1). Analysis: Calcd for $C_{10}H_7IN_2O_2S$: C, 34.70; H, 2.04; N, 8.09. Found: C, 34.73; H, 2.17; N, 8.16.

3-Nitro-*N*-phenylthiophen-2-amine (3e). Isolated as a yellow solid. Yield: 94%; mp = 67–68 °C. IR (KBr) ν_{max}: 3190, 3118, 3099, 1571, 1483, 1348, 1230, 1184 cm^{-1}. ^{1}H NMR (300 MHz, $CDCl_3$) δ_H: 6.31 (d, *J* = 6.3 Hz, 1H, H-5), 7.22–7.26 (m, 1H, H-4'), 7.35–7.39 (m, 3H, H-4, H-3',5'), 7.42–4.47 (m, 2H, H-2',6'), 10.38 (br s, 1H, NH); ^{13}C NMR (75 MHz, $CDCl_3$) δ_c: 107.1 (C-4), 120.2 (C-2',6'), 121.5 (C-4'), 125.6 (C-5), 128.0 (C-1'), 129.7 (C-3',5'), 138.8 (C-1), 155.5 (C-3); ESI-MS: m/z. Calcd: 220.03. Found: 255.26 (M+Cl^{35}). Analysis: Calcd for $C_{10}H_8N_2O_2S$: C, 54.53; H, 3.66; N, 12.72. Found: 54.16; H, 3.67; N, 12.61.

3-Nitro-*N*-(*p*-tolyl)thiophen-2-amine (3f). Isolated as a yellow solid. Yield: 94%; mp = 96–97 °C. IR (KBr) ν_{max}: 3244, 3095, 2920, 2853, 1560,1508, 1490, 1390, 1369, 1236,1197 cm^{-1}. ^{1}H NMR (300 MHz, $CDCl_3$) δ_H: 2.38 (s, 3H, Me), 6.27 (d, *J* = 6.0 Hz, 1H, H-5), 7.19–7.25 (m, 4H, H-2',3',5',6'), 7.34 (d, *J* = 6.3 Hz, 1H, H-4), 10.29 (br s, 1H, NH); ^{13}C NMR (75 MHz, $CDCl_3$) δ_c: 20.8 (Me), 107.0 (C-4), 120.7 (C-2',6'), 121.5 (C-4'), 127.7 (C-2), 130.2 (C-3',5'), 135.8 (C-1'), 136.4 (C-5), 156.4 (C-3); ESI-MS: m/z. Calcd: 234.05. Found: 235.10 (M+1). Analysis: Calcd for $C_{11}H_{10}N_2O_2S$: C, 56.39; H, 4.30; N, 11.96. Found: C, 56.01; H, 4.33; N, 11.71.

***N*-(4-Ethylphenyl)-3-nitrothiophen-2-amine (3g)**. Isolated as a pale yellow solid. Yield: 95%; mp = 74–75 °C. IR (KBr) ν_{max}: 3231, 3105, 2962, 2926, 2868, 1544, 1475, 1369,1242,1180 cm^{-1}. ^{1}H NMR (300 MHz, $CDCl_3$) δ_H: 1.26 (t, *J* = 7.5 Hz, 3H, CH_2CH_3), 2.68 (q, *J* = 7.5 Hz, 2H, CH_2CH_3), 6.28 (d, *J* = 6.0 Hz, 1H, H-5), 7.26–7.32 (m, 4H, H-2',3',5',6'), 7.35 (d, *J* = 6.3 Hz, 1H, H-4), 10.32 (br s, 1H, NH); ^{13}C NMR (75 MHz, $CDCl_3$) δ_c: 15.3 ($\underline{CH_2CH_3}$), 28.3 ($\underline{CH_2CH_3}$), 107.0 (C-4), 121.0 (C-2',6'), 121.8 (C-5), 127.9 (C-1'), 129.2 (C-3',5'), 136.8 (C-4'), 142.4 (C-2), 156.5 (C-3); ESI-MS: m/z. Calcd:

248.06. Found: 247.12 (M-1). Analysis: Calcd for $C_{12}H_{12}N_2O_2S$: C, 58.05; H, 4.87; N, 11.28. Found: C, 57.84; H, 4.95; N, 11.08.

***N*-(4-Isopropylphenyl)-3-nitrothiophen-2-amine (3h)**. Isolated as a pale yellow solid. Yield: 93%. IR (KBr) ν_{max}: 3230, 2956, 1544,1510, 1465, 1369, 1177 cm^{-1}. mp = 90–91 °C; ^1H NMR (300 MHz, $CDCl_3$) δ_H: 1.27 (d, *J* = 6.9 Hz, 6H, $CH(CH_3)_2$), 2.94 (sept, *J* = 6.9 Hz, 1H, $CH(CH_3)_2$), 6.28 (dd, *J* = 0.7, 6.1 Hz, 1H, H-5), 7.26–7.34 (m, 4H, H-2',3',5',6'), 7.35 (d, *J* = 6.3 Hz, 1H, H-4), 10.33 (br s, 1H, NH);^{13}C NMR (75 MHz, $CDCl_3$) δ_c: 23.8 ($CH(CH_3)_2$), 33.6 ($CH(CH_3)_2$), 107.0 (C-4), 120.7 (C-2',6'), 121.6 (C-5), 127.7 (C-1' and C-3',5'), 136.6 (C-4'), 146.8 (C-2), 156.3 (C-3); ESI-MS: m/z. Calcd: 262.08. Found: 261.15 (M-1). Analysis: Calcd for $C_{13}H_{14}N_2O_2S$: C, 59.52; H, 5.38; N, 10.68. Found: C, 59.40; H, 5.53; N, 10.62.

***N*-(4-Methoxyphenyl)-3-nitrothiophen-2-amine (3i)**. Isolated as an orange solid. Yield: 96%; mp = 98–99 °C. IR (KBr) ν_{max}: 3228, 3097, 2839, 1560,1508, 1500, 1388, 1224, 1195 cm^{-1}. ^1H NMR (300 MHz, $CDCl_3$) δ_H: 3.85 (s, 3H, OMe), 6.24 (d, *J* = 6.0 Hz, 1H, H-5), 6.95–6.98 (m, 2H, H-3',5'), 7.26–7.34 (m, 3H, H-4 and H-2',6'), 10.11 (br s, 1H, NH); ^{13}C NMR (75 MHz, $CDCl_3$) δ_c: 55.5 (OMe), 106.9 (C-4), 115.0 (C-3',5'), 121.8 (C-5), 123.5 (C-2',6'), 127.4 (C-1'), 132.1 (C-2), 158.0 (C-4'), 158.1 (C-3); ESI-MS: m/z. Calcd: 250.04. Found: 249.12 (M-1). Analysis: Calcd for $C_{11}H_{10}N_2O_3S$: C, 52.79; H, 4.03; N, 11.19. Found: C, ; H, 3.95; N, 10.81.

3-Nitro-*N*-(*o*-tolyl)thiophen-2-amine (3j). Isolated as a pale yellow solid. Yield: 93%; mp = 169–170 °C. IR (KBr) ν_{max}: 3236, 3101, 3118,1544,1465, 1375, 1340, 1227, 1180 cm^{-1}. ^1H NMR (300 MHz, $CDCl_3$) δ_H: 2.38 (s, 3H, Me), 6.28 (d, *J* = 6.3 Hz, 1H, H-5), 7.18–7.37 (m, 4H, H-3',4',5',6'), 7.51 (d, *J* = 8.1 Hz, 1H, H-4), 10.22 (br s, 1H, NH); ^{13}C

NMR (75 MHz, $CDCl_3$) δ_c: 17.7 (Me), 107.2 (C-4), 120.9 (C-4'), 121.7 (C-6'), 126.5 (C-5'), 127.3 (C-3'), 127.8 (C-2'), 130.9 (C-1'), 131.4 (C-5), 137.7 (C-2), 157.2 (C-3); ESI-MS: m/z. Calcd: 234.05. Found: 234.99 (M+1). Analysis: Calcd for $C_{11}H_{10}N_2O_2S$: C, 56.40; H, 4.30; N, 11.96. Found: C, 55.95; H, 4.41; N, 11.75.

***N*-(2-Methoxyphenyl)-3-nitrothiophen-2-amine (3k)**. Isolated as a yellow solid. Yield: 91%; mp = 123–124 °C. IR (KBr) ν_{max}: 3228, 3103,1560,1490, 1363, 1388, 1221, 1170 cm^{-1}. ^1H NMR (300 MHz, $CDCl_3$) δ_H: 3.96 (s, 3H, OMe), 6.35 (d, J = 6.0 Hz, 1H, H-5), 6.99 (dd, J = 1.2, 8.1 Hz, 1H, H-3'), 7.05 (td, J = 1.2, 7.8 Hz, 1H, H-5'), 7.16 (td, J = 1.2, 7.8 Hz, 1H, H-4'), 7.40 (d, J = 6.0 Hz, 1H, H-4), 7.55 (dd, J = 1.3, 7.9 Hz, 1H, H-6'), 10.93 (br s, 1H, NH); ^{13}C NMR (75 MHz, $CDCl_3$) δ_c: 55.9 (OMe), 107.2 (C-4), 111.0 (C-3'), 117.1 (C-6'), 120.6 (C-5'), 120.8 (C-4'), 121.7, 125.1 (C-5 and C-1'), 128.5 (C-2), 149.4 (C-2'), 153.8 (C-3); ESI-MS: m/z. Calcd: 250.04. Found: 249.08 (M-1). Analysis: Calcd for $C_{11}H_{10}N_2O_3S$: C, 52.79; H, 4.03; N, 11.19. Found: C, 52.27; H, 4.09; N, 10.95.

***N*-(3-Fluorophenyl)-3-nitrothiophen-2-amine (3l)**. Isolated as a yellow solid. Yield: 94%; mp = 101–102 °C. IR (KBr) ν_{max}: 3327,1560,1508, 1458, 1365, 1149, 1240, 1180 cm^{-1}. ^1H NMR (300 MHz, $CDCl_3$) δ_H: 6.38 (d, J = 6.3 Hz, 1H, H-5), 6.93 (m, 1H, H-4'), 7.11–7.17 (m, 2H, H-4, H-6'), 7.37–7.44 (m, 2H, H-2', H-5'), 10.39 (br s, 1H, NH); ^{13}C NMR (75 MHz, $CDCl_3$) δ_c: 107.2 (d, $^2J_{C, F}$ = 25.2 Hz, C-2'), 107.6 (C-4), 112.2 (d, $^2J_{C, F}$ = 21.1 Hz, C-4'), 115.6 (d, $^4J_{C, F}$ = 3.0 Hz, C-6'), 121.7 (C-5), 128.6 (C-2), 131.1 (d, $^3J_{C, F}$ = 9.4 Hz, C-5'), 140.4 (d, $^3J_{C, F}$ =10.0 Hz, C-1'), 154.3 (C-3), 163.2 (d, $^1J_{C, F}$ = 246.4 Hz, C-3'); ESI-MS: m/z. Calcd: 238.02. Found: 251.04 (M+2+Na). Analysis: Calcd for $C_{10}H_7FN_2O_2S$: C, 50.42; H, 2.96; N, 11.76. Found: C, 50.43; H, 3.03; N, 11.71.

***N*-(3-Bromophenyl)-3-nitrothiophen-2-amine (3m)**. Isolated as a pale yellow solid. Yield: 90%; mp = 132–133 °C. IR (KBr) ν_{max}: 3253, 3116, 3099, 1560, 1467, 1340, 1259, 1165, 1080 cm^{-1}. ^1H NMR (300 MHz, $CDCl_3$) δ_H: 6.37 (d, *J* = 6.3 Hz, 1H, H-5), 7.30–7.36 (m, 3H, H-4',5',6'), 7.38 (d, *J* = 6.3 Hz, 1H, H-4), 7.55 (s, 1H, H-2'), 10.31 (br s, 1H, NH); ^{13}C NMR (75 MHz, $CDCl_3$) δ_c: 107.6 (C-4), 118.7 (C-2'), 121.8 (C-6'), 123.1 (C-3'), 123.4 (C-4'), 128.5 (C-2), 128.7 (C-5), 131.1 (C-5'), 140.2 (C-1'), 154.4 (C-3); ESI-MS: m/z. Calcd: 297.94. Found: 296.98 (M-1), 298.99 (M+2). Analysis: Calcd for $C_{10}H_7BrN_2O_2S$: C, 40.15; H, 2.36; N, 9.36. Found: 40.17; H, 2.37; N, 11.71.

3-Nitro-*N*-(3-(trifluoromethyl)phenyl)thiophen-2-amine (3n). Isolated as a pale yellow solid. Yield: 89%; mp = 106–107 °C. IR (KBr) ν_{max}: 3257, 3101, 1560,1508, 1491, 1327, 1234, 1205, 1170 cm^{-1}. ^1H NMR (300 MHz, $CDCl_3$) δ_H: 6.39 (d, *J* = 6.3 Hz, 1H, H-5), 7.41 (d, *J* = 6.0 Hz, 1H, H-4), 7.44–7.59 (m, 3H, H-4',5',6'), 7.65 (s, 1H, H-2'), 10.41 (br s, 1H, NH); ^{13}C NMR (75 MHz, $CDCl_3$) δ_c: 107.6 (C-4), 116.8 (q, $^3J_{C,F}$ = 3.8 Hz, C-2'), 121.6 (C-6'), 121.9, 122.0 (q, $^3J_{C,F}$ = 3.6 Hz, C-4'), 123.2 (C-1'), 127.1 (q, $^1J_{C,F}$ = 271.5 Hz, CF_3), 130.5 (C-5), 132.1(q, $^2J_{C,F}$ = 32.7 Hz, C-3'), 139.6 (C-2), 154.2 (C-3); ESI-MS: m/z. Calcd: 288.02. Found: 287.04 (M-1). Analysis: Calcd for $C_{11}H_7F_3N_2O_2S$: C, 45.84; H, 2.45; N, 9.72. Found: C, 45.53, H, 2.52; N, 9.88.

3-Nitro-*N*-(*m*-tolyl)thiophen-2-amine (3o). Isolated as a yellow solid. Yield: 94%; mp = 83–84 °C. IR (KBr) ν_{max}: 3103, 2918, 2852,1572, 1491, 1391, 1357, 1213, 1171 cm^{-1}. ^1H NMR (300 MHz, $CDCl_3$) δ_H: 6.30 (dd, *J* = 0.6, 6.0 Hz, 1H, H-5), 7.06 (m, 1H, H-4'), 7.19 (m, 2H, H-4, H-5'), 7.30–7.37 (m, 2H, H-2',5'), 10.35 (br s, 1H, NH); ^{13}C NMR (75 MHz, $CDCl_3$) δ_c: 21.4 (Me), 107.1 (C-4), 117.4 (C-6'), 121.2 (C-4'), 121.7 (C-2'), 126.6 (C-5'), 128.1 (C-2), 129.6 (C-5), 138.9 (C-1'), 140.0 (C-3'), 155.7 (C-3); ESI-MS: m/z.

Calcd: 234.05. Found: 233.13 (M-1). Analysis: Calcd for $C_{11}H_{10}N_2O_2S$: C, 56.40; H, 4.30; N, 11.96. Found: 56.25; H, 4.42; N, 11.67.

***N*-(3-Methoxyphenyl)-3-nitrothiophen-2-amine (3p)**. Isolated as an orange solid. Yield: 95%; mp = 111–112 °C. IR (KBr) ν_{max}: 3157, 3116, 3101,1562, 1508, 1481, 1369, 1355, 1215, 1161 cm^{-1}. ^1H NMR (300 MHz, $CDCl_3$) δ_H: 3.84 (s, 3H, OMe), 6.32, (d, *J* = 6.0 Hz, 1H, H-5), 6.78 (dd, *J* = 2.4, 8.4 Hz, 1H, H-4'), 6.91 (t, *J* = 2.1 Hz, 1H, H-2'), 6.96 (dd, *J* = 2.2, 7.9 Hz, 1H, H-6'), 7.33 (t, *J* = 8.1 Hz, 1H, H-5'), 7.36 (d, *J* = 6.0 Hz, 1H, H-4), 10.37 (br s, 1H, NH); ^{13}C NMR (75 MHz, $CDCl_3$) δ_c: 55.3 (OMe), 106.0 (C-2'), 107.4 (C-4), 111.1 (C-6'), 112.3 (C-4'), 121.5 (C-5), 128.1 (C-1'), 130.5 (C-5'), 139.9 (C-2), 155.2 (C-3), 160.6 (C-3'); ESI-MS: m/z. Calcd: 250.04. Found: 249.10 (M-1). Analysis: Calcd for $C_{11}H_{10}N_2O_3S$: C, 52.79; H, 4.03; N, 11.19. Found: C, 52.63; H, 4.10; N, 11.09.

***N*-(2,4-Dimethylphenyl)-3-nitrothiophen-2-amine (3q)**. Isolated as a yellow solid. Yield: 92%; mp = 114–115 °C. IR (KBr) ν_{max}: 3186, 3122, 3105,1560, 1491, 1377, 1354, 1223, 1167 cm^{-1}. ^1H NMR (300 MHz, $CDCl_3$) δ_H: 2.33 (s, 3H, Me), 2.36 (s, 3H, Me), 6.24 (dd, *J* = 0.9, 6.3 Hz, 1H, H-5), 7.09–7.14 (m, 2H, H-3' and H-6'), 7.32–7.37 (m, 2H, H-4 and H-5'), 10.08 (br s, 1H, NH); ^{13}C NMR (75 MHz, $CDCl_3$) δ_c: 17.6 (Me), 20.9 (Me), 107.1 (C-4), 121.8 (C-6'), 127.4 (C-1'), 127.8 (C-5), 131.3 (C-3'), 132.1 (C-2'), 135.2 (C-4'), 136.9 (C-2), 158.3 (C-3); ESI-MS: m/z. Calcd: 248.06. Found: 249.06 (M+1). Analysis: Calcd for $C_{12}H_{12}N_2O_2S$: C, 58.05; H, 4.87; N, 11.28. Found: C, 58.39; H, 4.63; N, 10.90.

***N*-(Naphthalen-1-yl)-3-nitrothiophen-2-amine (3r)**. Isolated as a yellow solid. Yield: 91%; mp = 153–154 °C. IR (KBr) ν_{max}: 3290, 3109, 3091,1558, 1508, 1458, 1390, 1211, 1182 cm^{-1}. ^1H NMR (300 MHz, $CDCl_3$) δ_H: 6.28 (d, *J* = 6.3 Hz, 1H, H-

5), 7.38 (d, J = 6.0 Hz, 1H, H-4), 7.50–7.56 (m, 1H, H-7’), 7.59–7.64 (m, 2H, H-2’,6’), 7.71 (d, J = 7.5 Hz, 1H, H-4’), 7.83 (d, J = 8.4 Hz, 1H, H-3’), 7.92–7.95 (m, 1H, H-5’), 8.05–8.06 (m, 1H, H-8’), 10.67 (br s, 1H, NH); ^{13}C NMR (75 MHz, $CDCl_3$) δ_c: 107.4 (C-4), 118.8 (C-2’), 121.0 (C-4’), 121.7 (C-8’), 125.0 (C-8a’), 125.4 (C-7’), 127.0 (C-1’), 127.2 (C-6’), 127.26 (C-3’), 127.28 (C-5), 128.6 (C-5’), 134.4 (C-4a’), 135.1 (C-2), 158.1 (C-3); ESI-MS: m/z. Calcd: 270.05. Found: 271.07 (M+1). Analysis: Calcd for $C_{14}H_{10}N_2O_2S$: C, 62.21; H, 3.73; N, 10.36. Found: 62.48; H, 4.05; N, 9.97.

***N*-Methyl-3-nitrothiophen-2-amine (3s)**. Isolated as a yellow solid. Yield: 93%; mp = 69–70 °C. IR (KBr) ν_{max}: 3327, 2922, 2852, 1560, 1344, 1261, 1195, 1026 cm^{-1}. ^{1}H NMR (300 MHz, $CDCl_3$) δ_H: 3.14 (d, J = 5.4 Hz, 3H, Me), 6.24 (d, J = 6.0 Hz, 1H, H-5), 7.27 (d, J = 6.3 Hz, 1H, H-4), 8.41 (br s, 1H, NH); ^{13}C NMR (75 MHz, $CDCl_3$) δ_c: 33.7 (Me), 106.5 (C-4), 122.1 (C-5), 125.5 (C-2), 162.5 (C-3); ESI-MS: m/z. Calcd: 158.01. Found: 158.98 (M+1). Analysis: Calcd for $C_5H_6N_2O_2S$: C, 37.97; H, 3.82; N, 17.71. Found: C, 38.45; H, 3.92; N, 17.37.

3-Nitro-*N*-propylthiophen-2-amine (3t). Isolated as a yellow liquid. Yield: 95%. IR (KBr) ν_{max}: 3308, 3101, 2964, 2931,2874,1572, 1516, 1400, 1336, 1263, 1186, 1084 cm^{-1}. ^{1}H NMR (300 MHz, $CDCl_3$) δ_H: 1.05 (t, J = 7.3 Hz, 3H, $CH_2CH_2CH_3$), 1.80 (sext, J = 7.2 Hz, 2H, $CH_2CH_2CH_3$), 3.32 (q, J = 6.9 Hz, 2H, $CH_2CH_2CH_3$), 6.21 (dd, J = 0.9, 6.0 Hz, 1H, H-5), 7.25 (d, J = 6.0 Hz, 1H, H-4), 8.47 (br s, 1H, NH); ^{13}C NMR (75 MHz, $CDCl_3$) δ_c: 11.2 ($CH_2CH_2CH_3$), 22.0 ($CH_2CH_2CH_3$), 49.7 ($CH_2CH_2CH_3$), 106.4 (C-4), 122.1 (C-5), 125.7 (C-2), 161.5 (C-3); ESI-MS: m/z.

Calcd: 186.05. Found: 187.00 (M+1). Analysis: Calcd for $C_7H_{10}N_2O_2S$: C, 45.15; H, 5.41; N, 15.04. Found: C, 45.50; H, 5.55; N, 14.86.

***N*-Butyl-3-nitrothiophen-2-amine (3u)**. Isolated as a yellow solid. Yield: 92%; mp = 50–51 °C. IR (KBr) ν_{max}: 3331, 2931, 2868, 1570, 1381, 1259, 1055 cm^{-1}. ^1H NMR (300 MHz, $CDCl_3$) δ_H: 0.98 (t, *J* = 7.3 Hz, 3H, $CH_2CH_2CH_2CH_3$), 1.47 (sext, *J* = 7.4 Hz, 2H, $CH_2CH_2CH_2CH_3$), 1.70–1.80 (m, 2H, $CH_2CH_2CH_2CH_3$), 3.35 (q, *J* = 6.7 Hz, 2H, $CH_2CH_2CH_2CH_3$), 6.21 (dd, *J* = 1.0, 6.1 Hz, 1H, H-5), 7.25 (d, *J* = 6.3 Hz, 1H, H-4), 8.45 (br s, 1H, NH); ^{13}C NMR (75 MHz, $CDCl_3$) δ_c: 13.5 ($CH_2CH_2CH_2CH_3$), 19.9 ($CH_2CH_2CH_2CH_3$), 30.6 ($CH_2CH_2CH_2CH_3$), 47.7 ($CH_2CH_2CH_2CH_3$), 106.4 (C-4), 122.1 (C-5), 125.6 (C-2), 161.4 (C-3); ESI-MS: m/z. Calcd: 200.06. Found: 201.02 (M+1). Analysis: Calcd for $C_8H_{12}N_2O_2S$: C, 47.98; H, 6.04; N, 13.99. Found: 48.35; H, 6.01; N, 13.66.

***N*-Isopropyl-3-nitrothiophen-2-amine (3v)**. Isolated as a yellow liquid. Yield: 93%. IR (KBr) ν_{max}: 3302, 3097, 2974, 2928, 1562, 1400, 1334, 1265, 1153 cm^{-1}. ^1H NMR (300 MHz, $CDCl_3$) δ_H: 1.39 (d, *J* = 6.3 Hz, 6H, $CH(CH_3)_2$), 3.58–3.67 (m, 1H, $CH(CH_3)_2$), 6.22 (d, *J* = 6.3 Hz, 1H, H-5), 7.25 (d, *J* = 6.0 Hz, 1H, H-4), 8.40 (br s, 1H, NH); ^{13}C NMR (75 MHz, $CDCl_3$) δ_c: 22.3 ($CH(CH_3)_2$), 50.6 ($CH(CH_3)_2$), 106.4 (C-4), 122.1 (C-5), 125.7 (C-2), 160.1 (C-3); ESI-MS: m/z. Calcd: 186.05. Found: 187.08 (M+1). Analysis: Calcd for $C_7H_{10}N_2O_2S$: C, 45.15; H, 5.41; N, 15.04. Found: C, 45.54; H, 5.56; N, 14.83.

***N*-Cyclopropyl-3-nitrothiophen-2-amine (3w)**. Isolated as a yellow liquid. Yield: 94%. IR (KBr) ν_{max}: 3321, 3100, 3009, 2872, 1562, 1516, 1400, 1332, 1259, 1188, 1084 cm^{-1}. ^1H NMR (300 MHz, $CDCl_3$) δ_H: 0.79–1.02 (m, 4H, H-2’,3’), 2.69–2.76 (m, 1H, H-1’), 6.27 (d, *J* = 6.0 Hz, 1H, H-5), 7.27 (d, *J* = 6.0 Hz, 1H, H-4), 8.38 (br s, 1H, NH); ^{13}C NMR (75

MHz, $CDCl_3$) δ_c: 7.7 (C-2',3'), 28.3 (C-1'), 107.6 (C-4), 121.9 (C-5), 125.9 (C-2), 162.3 (C-3); ESI-MS: m/z. Calcd: 184.03. Found: 183.06 (M-1). Analysis: Calcd for $C_7H_8N_2O_2S$: C, 45.64; H, 4.38; N, 15.21. Found: C, 45.29; H, 4.49; N, 14.85.

***N*-Cyclohexyl-3-nitrothiophen-2-amine (3x)**. Isolated as a yellow solid. Yield: 95%; mp = 130–131 °C. IR (KBr) ν_{max}: 3285, 3115, 3096, 2931, 2852, 1570, 1389, 1240, 1076 cm^{-1}. ^1H NMR (300 MHz, $CDCl_3$) δ_H: 1.29–1.50 (m, 5H, H-3',5',6'), 1.65–1.68 (m, 1H, H-4'), 1.80–1.83 (m, 2H, H-2',6'), 2.09–2.14 (m, 2H, H2',6'), 3.23–3.31 (m, 1H, H-1'), 6.20 (d, *J* = 6.3 Hz, 1H, H-5), 7.24 (d, *J* = 6.0 Hz, 1H, H-4), 8.52 (br s, 1H, NH); ^{13}C NMR (75 MHz, $CDCl_3$) δ_c: 24.3 (C-3',5'), 25.1 (C-4'), 32.1 (C-2',6'), 57.4 (C-1'), 106.3 (C-4), 122.0 (C-5), 125.6 (C-2), 160.0 (C-3); ESI-MS: m/z. Calcd: 226.08. Found: 227.10 (M+1). Analysis: Calcd for $C_{10}H_{14}N_2O_2S$: C, 53.08; H, 6.24; N, 12.38. Found: C, 53.33; H, 6.24; N, 12.30.

***N*-Benzyl-3-nitrothiophen-2-amine (3y)**. Isolated as a yellow solid. Yield: 92 %; mp = 55–56 °C; IR (KBr) ν_{max}: 3287, 3115, 3093, 2922, 2954, 1560, 1386, 1219, 1066 cm $^{-1}$. ^1H NMR (300 MHz, $CDCl_3$) δ_H: 4.53 (d, *J* = 5.7 Hz, 2H, CH_2Ph), 6.22 (d, *J* = 6.0 Hz, 1H, H-5), 7.28 (d, *J* = 6.0 Hz, 1H, H-4), 7.37–7.42 (m, 5H, CH_2*Ph*), 8.72 (br s, 1H, NH); ^{13}C NMR (75 MHz, $CDCl_3$) δ_c: 51.7 (*C*H_2Ph), 106.7 (C-4), 122.3 (C-2 and C-5), 127.6 (C-2',6'), 128.4 (C-4'), 129.0 (C-3',5'), 135.2 (C-1'), 160.8 (C-3); ESI-MS: m/z. Calcd: 234.05. Found: 235.07 (M+1). Analysis: Calcd for $C_{11}H_{10}N_2O_2S$: C, 56.40; H, 4.30; N, 11.96. Found: C, 56.89; H, 4.79; N, 11.83.

(*R*)-3-Nitro-*N*-(1-phenylethyl)thiophen-2-amine (3z). Isolated as a yellow solid. Yield: 96 %; mp = 71–72 °C. IR (KBr) ν_{max}: 3319, 3120, 3100, 2974, 2926, 1545, 1342, 1254, 1068 cm^{-1}. ^1H NMR (300 MHz, $CDCl_3$) δ_H: 1.70 (d, *J* = 6.9 Hz, 3H, Me), 4.52 (q, *J* = 6.7

Hz, 1H, C*H*Me), 6.13 (dd, J = 0.9, 6.0 Hz, 1H, H-5), 7.23 (d, J = 6.0 Hz, 1H, H-4), 8.80 (br s, 1H, NH); ^{13}C NMR (75 MHz, $CDCl_3$) δ_c: 23.9 (Me), 57.9 (*C*HMe), 107.2 (C-4), 121.7 (C-2 and C-5), 126.1 (C-2',6'), 128.1 (C-4'), 128.9 (C-3',5'), 140.9 (C-1'), 159.8 (C-3); ESI-MS: m/z. Calcd: 248.06. Found: 249.04 (M+1). Analysis: Calcd for $C_{12}H_{12}N_2O_2S$: C, 58.05; H, 4.87; N, 11.28. Found: C, 58.19; H, 4.91; N, 11.29.

2. Structure determination of 3c using NMR spectroscopic data

The structure of *N*-(4-bromophenyl)-3-nitrothiophen-2-amine (**3c**) was deduced from one- and two-dimensional NMR spectroscopic data. The structural elucidation of **3c** using NMR spectroscopy is discussed below.

The H-4 appears as a doublet at 7.37 ppm (*J* = 6.0 Hz), which shows HMBCs with C-2, C-3 and C-5 at 154.9, 128.5 and 107.4 ppm, respectively. Likewise, the H-5 appears as a doublet at 6.34 ppm (*J* = 6.3 Hz), which shows HMBCs with C-2, C-3, C-4 at 154.9, 128.5 and 122.0 ppm, respectively. The NH peak appears as a broad singlet at 10.30 ppm.

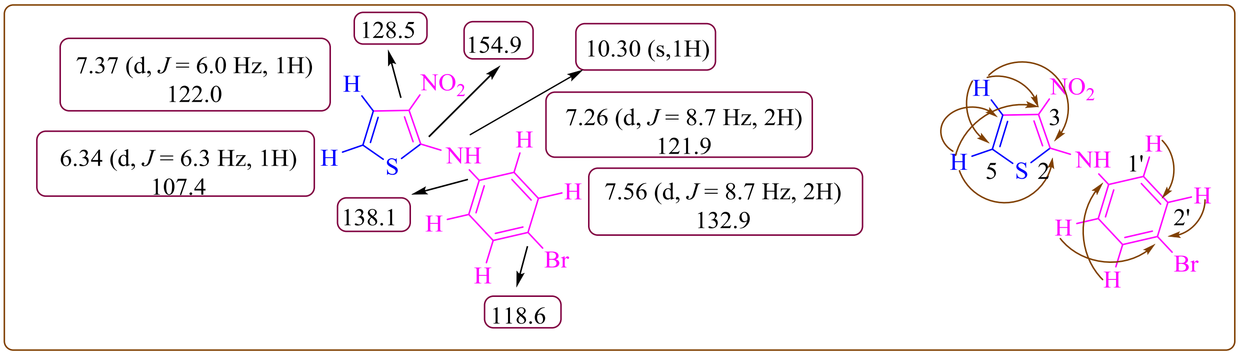

3. Copies of spectra

No.	List of Figures	Page
1	^{1}H NMR Spectrum **1d**	S20
2	^{13}C NMR Spectrum of **1d**	S20
3	ESI mass spectrum of Spectrum of **1d**	S21
4	^{1}H NMR Spectrum **1g**	S21
5	^{13}C NMR Spectrum of **1g**	S22
6	ESI mass spectrum of Spectrum of **1g**	S22
7	^{1}H NMR Spectrum **1m**	S23
8	^{13}C NMR Spectrum of **1m**	S23
9	ESI mass spectrum of Spectrum of **1m**	S24
10	^{1}H NMR Spectrum **1q**	S24
11	^{13}C NMR Spectrum of **1q**	S25
12	ESI mass spectrum of Spectrum of **1q**	S25
13	^{1}H NMR Spectrum **1w**	S26
14	^{13}C NMR Spectrum of **1w**	S26
15	ESI mass spectrum of Spectrum of **1w**	S27
16	^{1}H NMR Spectrum **1z**	S27
17	^{13}C NMR Spectrum of **1z**	S28
18	ESI mass spectrum of Spectrum of **1z**	S28
19	^{1}H NMR Spectrum **3a**	S29
20	^{13}C NMR Spectrum of **3a**	S29
21	ESI mass spectrum of Spectrum of **3a**	S30
22	^{1}H NMR Spectrum **3b**	S30
23	^{13}C NMR Spectrum of **3b**	S31
24	ESI mass spectrum of Spectrum of **3b**	S31
25	^{1}H NMR Spectrum **3c**	S32
26	^{13}C NMR Spectrum of **3c**	S32
27	DEPT Spectrum of **3c**	S33
28	H, H-COSY Spectrum of **3c**	S33
29	HMBC Spectrum of **3c**	S34
30	ESI mass spectrum of Spectrum of **3c**	S34
31	^{1}H NMR Spectrum **3d**	S35
32	^{13}C NMR Spectrum of **3d**	S35
33	ESI mass spectrum of Spectrum of **3d**	S36

34	^{1}H NMR Spectrum **3e**	S36
35	^{13}C NMR Spectrum of **3e**	S37
36	ESI mass spectrum of Spectrum of **3e**	S37
37	^{1}H NMR Spectrum **3f**	S38
38	^{13}C NMR Spectrum of **3f**	S38
39	ESI mass spectrum of Spectrum of **3f**	S39
40	^{1}H NMR Spectrum **3g**	S39
41	^{13}C NMR Spectrum of **3g**	S40
42	ESI mass spectrum of Spectrum of **3g**	S40
43	^{1}H NMR Spectrum **3h**	S41
44	^{13}C NMR Spectrum of **3h**	S41
45	ESI mass spectrum of Spectrum of **3h**	S42
46	^{1}H NMR Spectrum **3i**	S42
47	^{13}C NMR Spectrum of **3i**	S43
48	ESI mass spectrum of Spectrum of **3i**	S43
49	^{1}H NMR Spectrum **3j**	S44
50	^{13}C NMR Spectrum of **3j**	S44
51	ESI mass spectrum of Spectrum of **3j**	S45
52	^{1}H NMR Spectrum **3k**	S45
53	^{13}C NMR Spectrum of **3k**	S46
54	ESI mass spectrum of Spectrum of **3k**	S46
55	^{1}H NMR Spectrum **3l**	S47
56	^{13}C NMR Spectrum of **3l**	S47
57	ESI mass spectrum of Spectrum of **3l**	S48
58	^{1}H NMR Spectrum **3m**	S48

59	^{13}C NMR Spectrum of **3m**	S49
60	ESI mass spectrum of Spectrum of **3m**	S49
61	^{1}H NMR Spectrum **3n**	S50
62	^{13}C NMR Spectrum of **3n**	S50
63	ESI mass spectrum of Spectrum of **3n**	S51
64	^{1}H NMR Spectrum **3o**	S51
65	^{13}C NMR Spectrum of **3o**	S52
66	ESI mass spectrum of Spectrum of **3o**	S52
67	^{1}H NMR Spectrum **3p**	S53
68	^{13}C NMR Spectrum of **3p**	S53
69	ESI mass spectrum of Spectrum of **3p**	S54
70	^{1}H NMR Spectrum **3q**	S54
71	^{13}C NMR Spectrum of **3q**	S55
72	ESI mass spectrum of Spectrum of **3q**	S55
73	^{1}H NMR Spectrum **3r**	S56
74	^{13}C NMR Spectrum of **3r**	S56
75	ESI mass spectrum of Spectrum of **3r**	S57
76	^{1}H NMR Spectrum **3s**	S57
77	^{13}C NMR Spectrum of **3s**	S58
78	ESI mass spectrum of Spectrum of **3s**	S58
79	^{1}H NMR Spectrum **3t**	S59
80	^{13}C NMR Spectrum of **3t**	S59
81	ESI mass spectrum of Spectrum of **3t**	S60
82	^{1}H NMR Spectrum **3u**	S60
83	^{13}C NMR Spectrum of **3u**	S61

84	ESI mass spectrum of Spectrum of **3u**	S61
85	^{1}H NMR Spectrum **3v**	S62
86	^{13}C NMR Spectrum of **3v**	S62
87	ESI mass spectrum of Spectrum of **3v**	S63
88	^{1}H NMR Spectrum **3w**	S63
89	^{13}C NMR Spectrum of **3w**	S64
90	ESI mass spectrum of Spectrum of **3w**	S64
91	^{1}H NMR Spectrum **3x**	S65
92	^{13}C NMR Spectrum of **3x**	S65
93	ESI mass spectrum of Spectrum of **3x**	S66
94	^{1}H NMR Spectrum **3y**	S66
95	^{13}C NMR Spectrum of **3y**	S67
96	ESI mass spectrum of Spectrum of **3y**	S67
97	^{1}H NMR Spectrum **3z**	S68
98	^{13}C NMR Spectrum of **3z**	S68
99	ESI mass spectrum of Spectrum of **3z**	S69

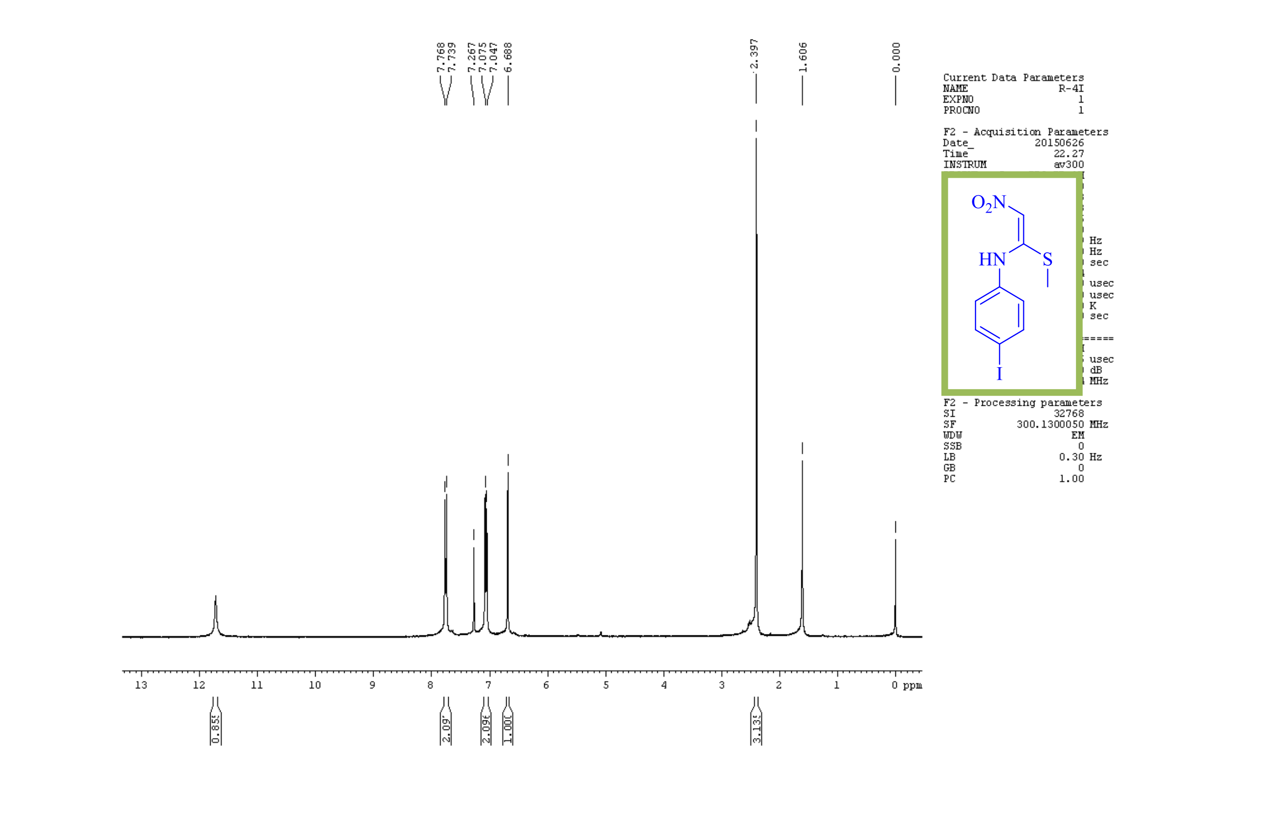

Figure 1 ^{1}H NMR Spectrum **1d** ($CDCl_3$)

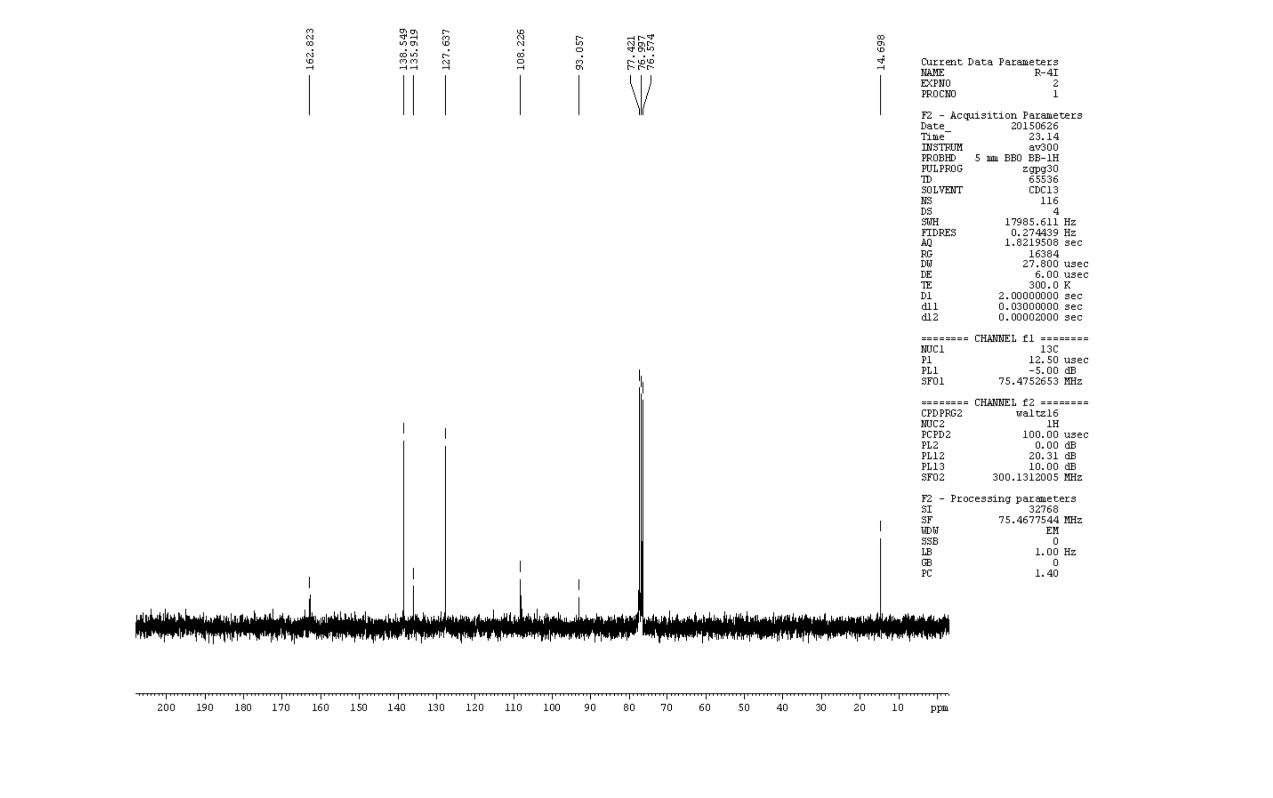

Figure 2 ^{13}C NMR Spectrum of **1d** ($CDCl_3$)

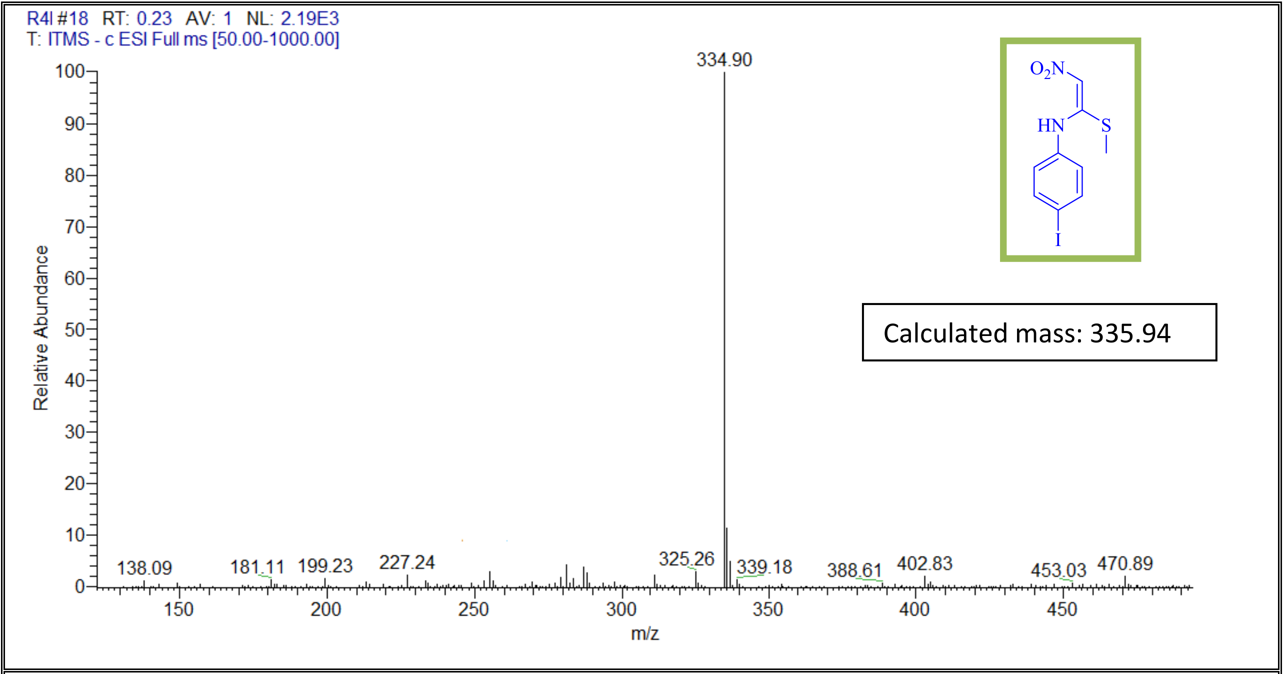

Figure 3 ESI mass spectrum of **1d**

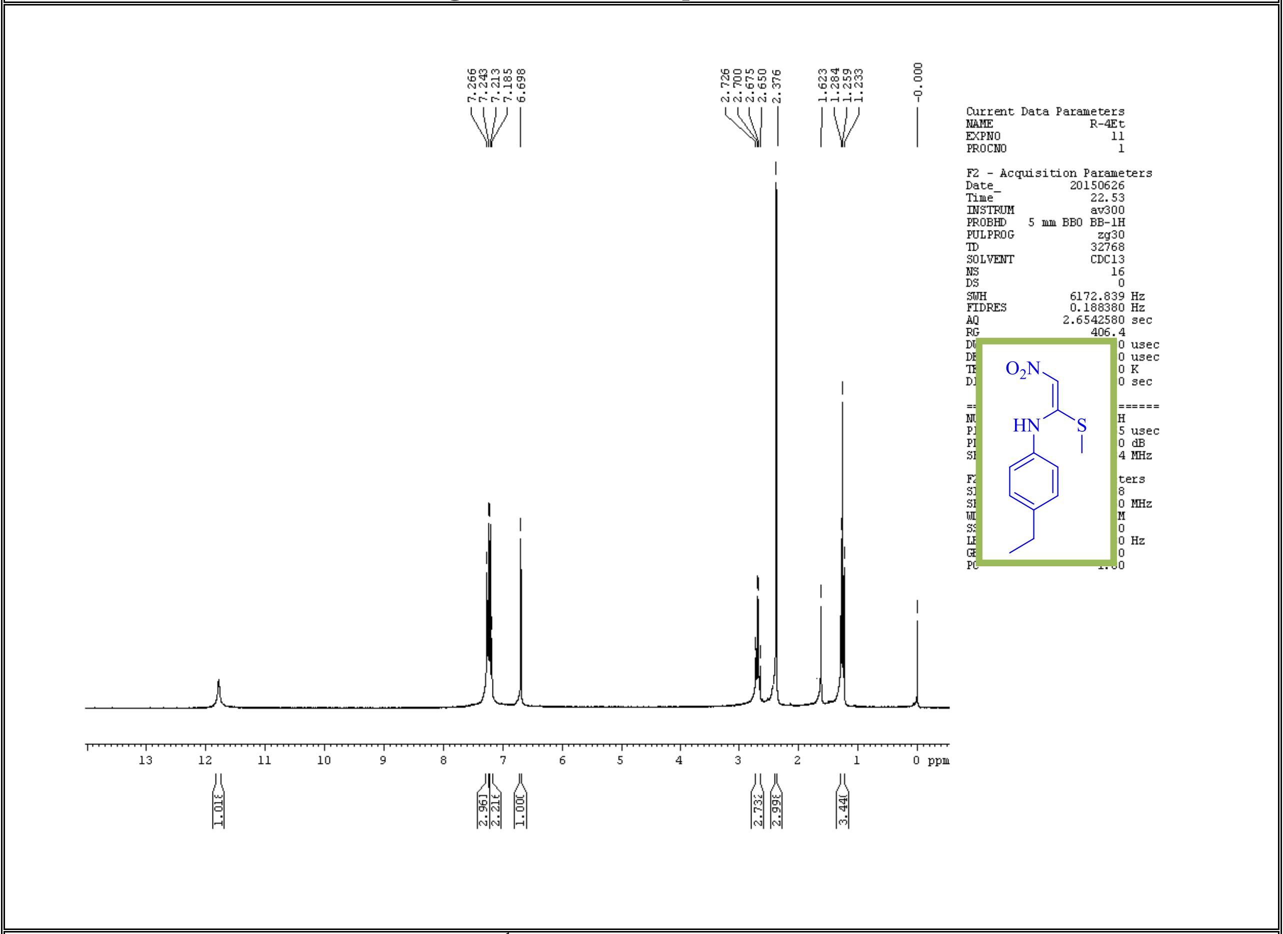

Figure 4 ^{1}H NMR Spectrum **1g** ($CDCl_3$)

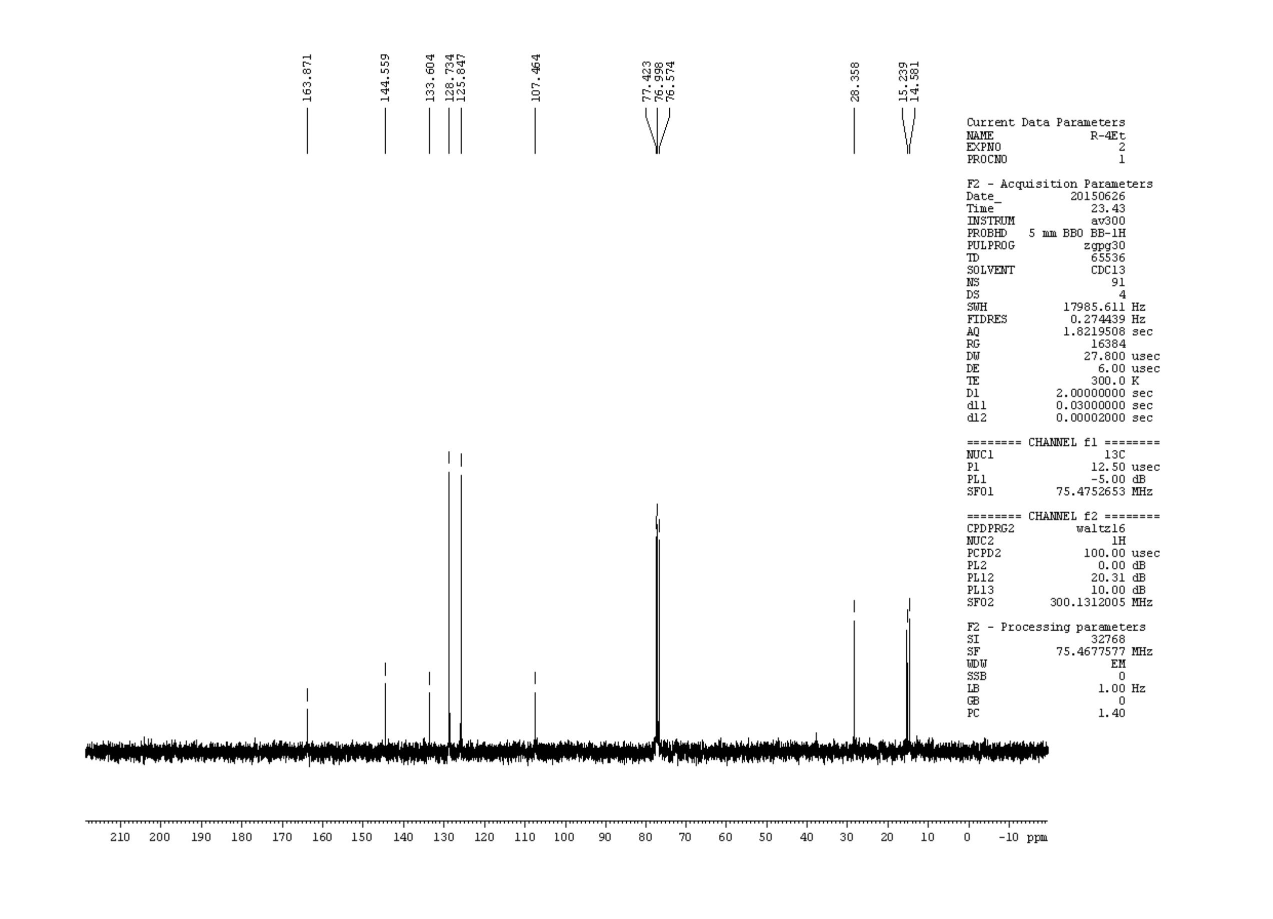

Figure 5 ^{13}C NMR Spectrum of **1g** ($CDCl_3$)

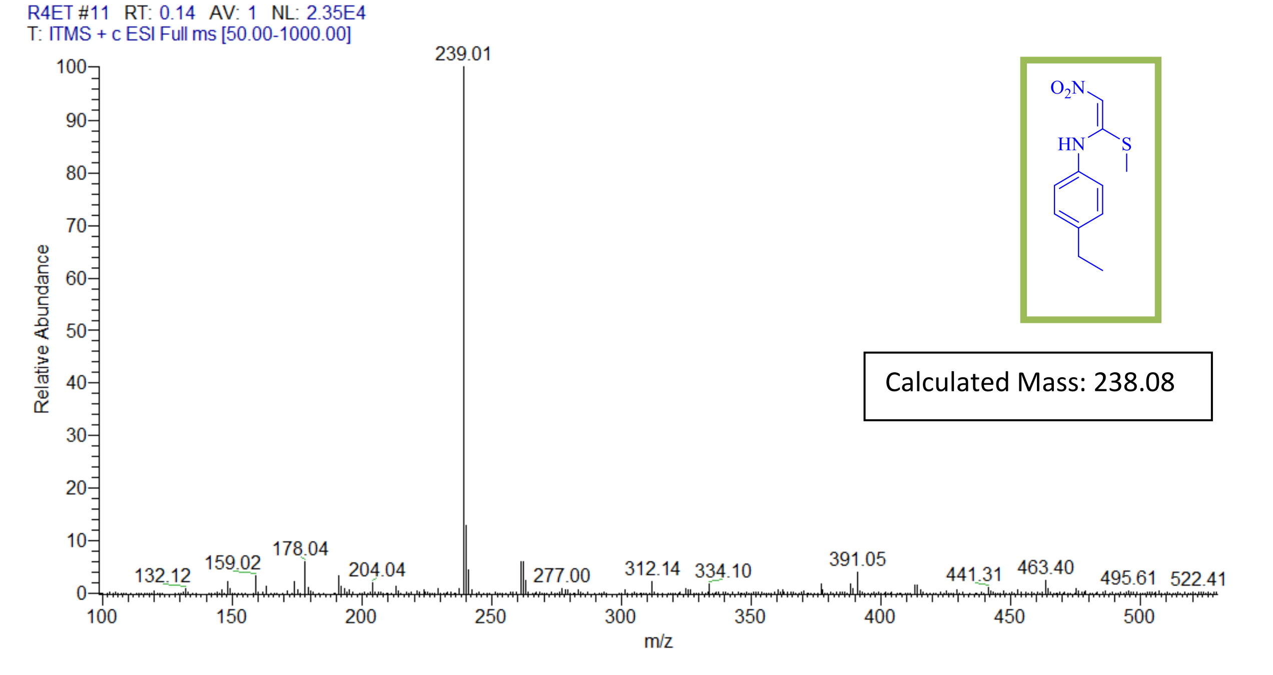

Figure 6 ESI mass spectrum of **1g**

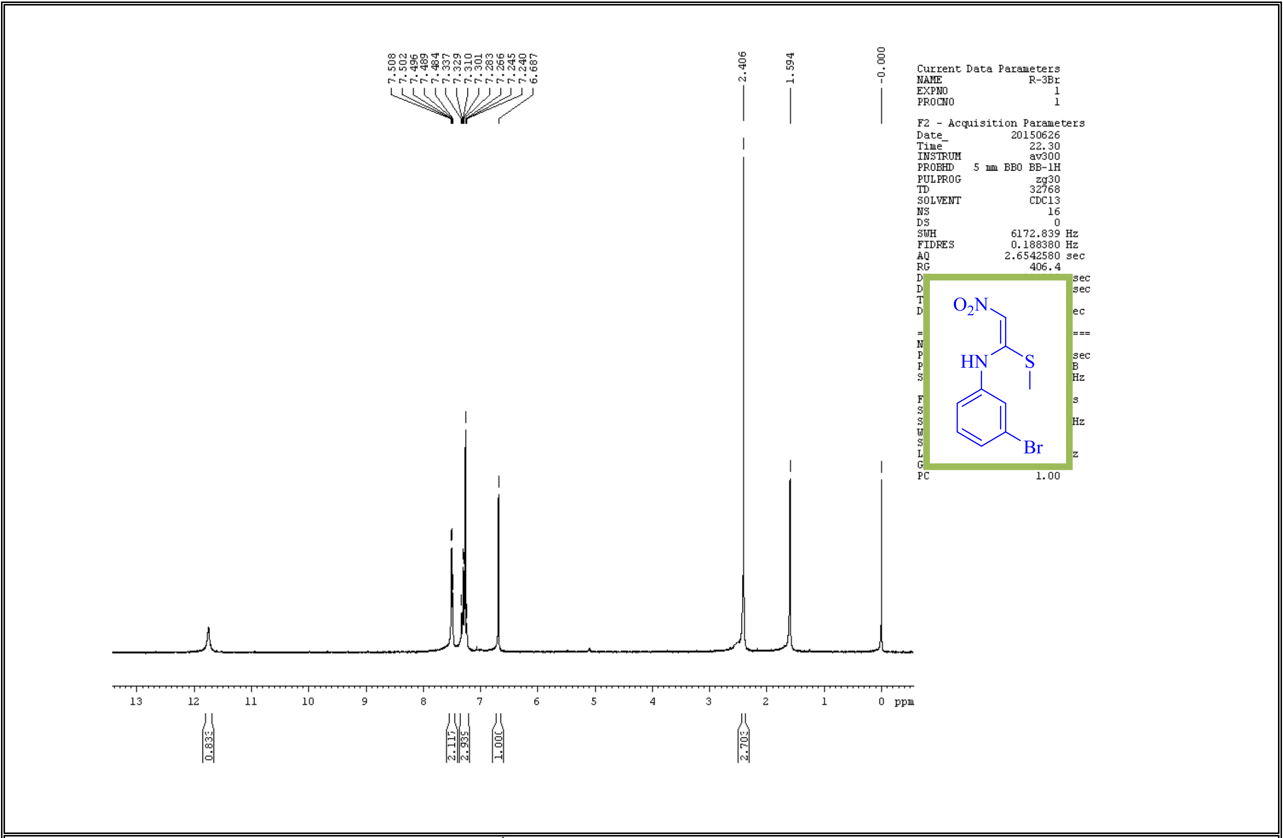

Figure 7 ^{1}H NMR Spectrum **1m** ($CDCl_3$)

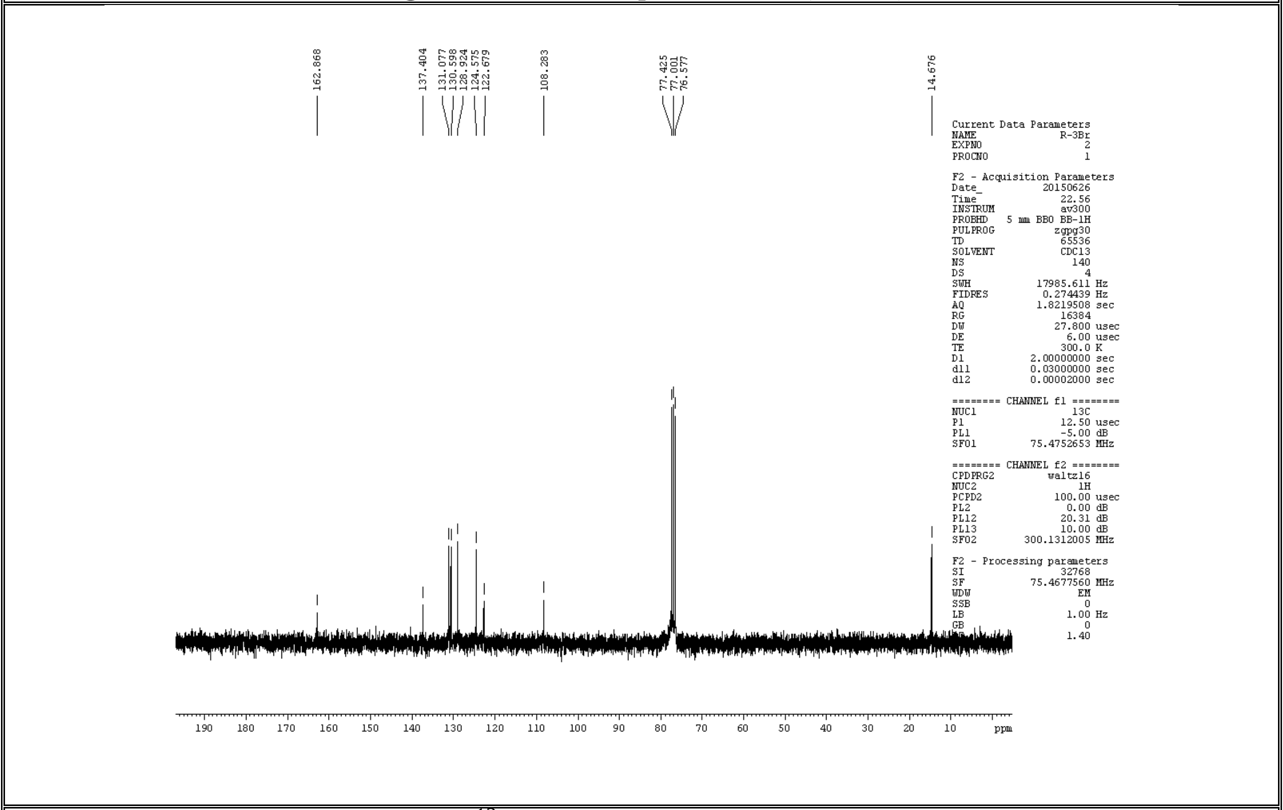

Figure 8 ^{13}C NMR Spectrum of **1m** ($CDCl_3$)

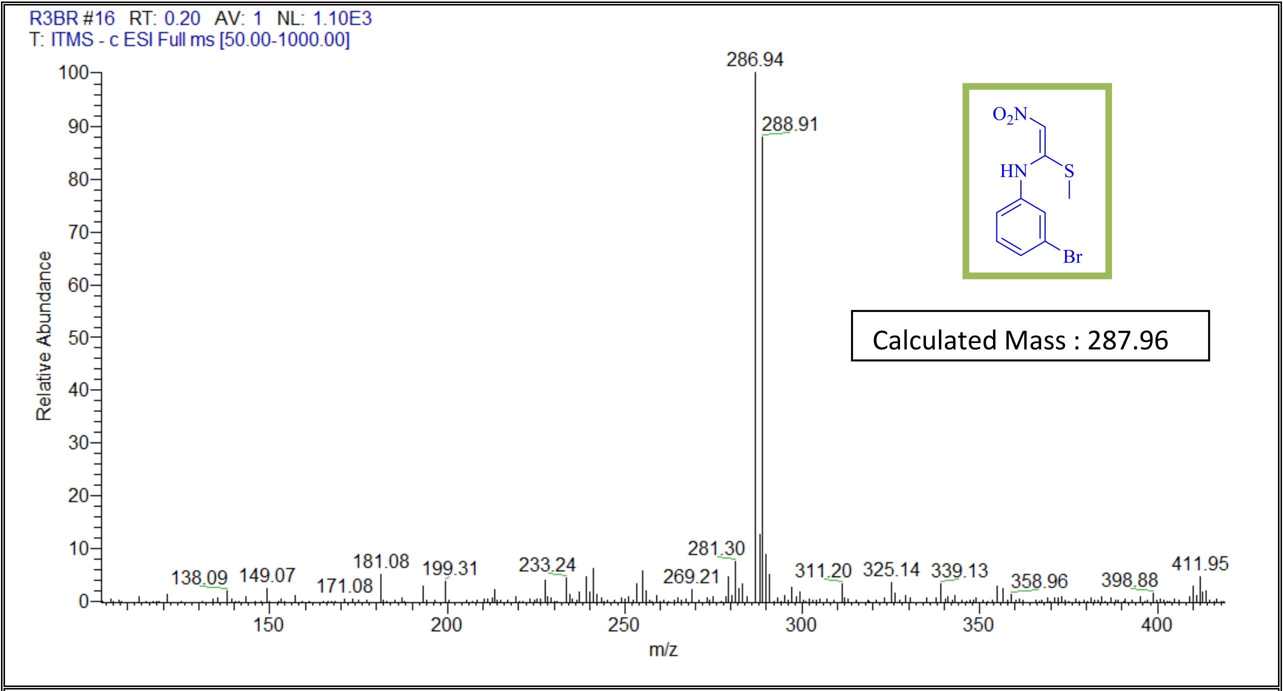

Figure 9 ESI mass spectrum of Spectrum of **1m**

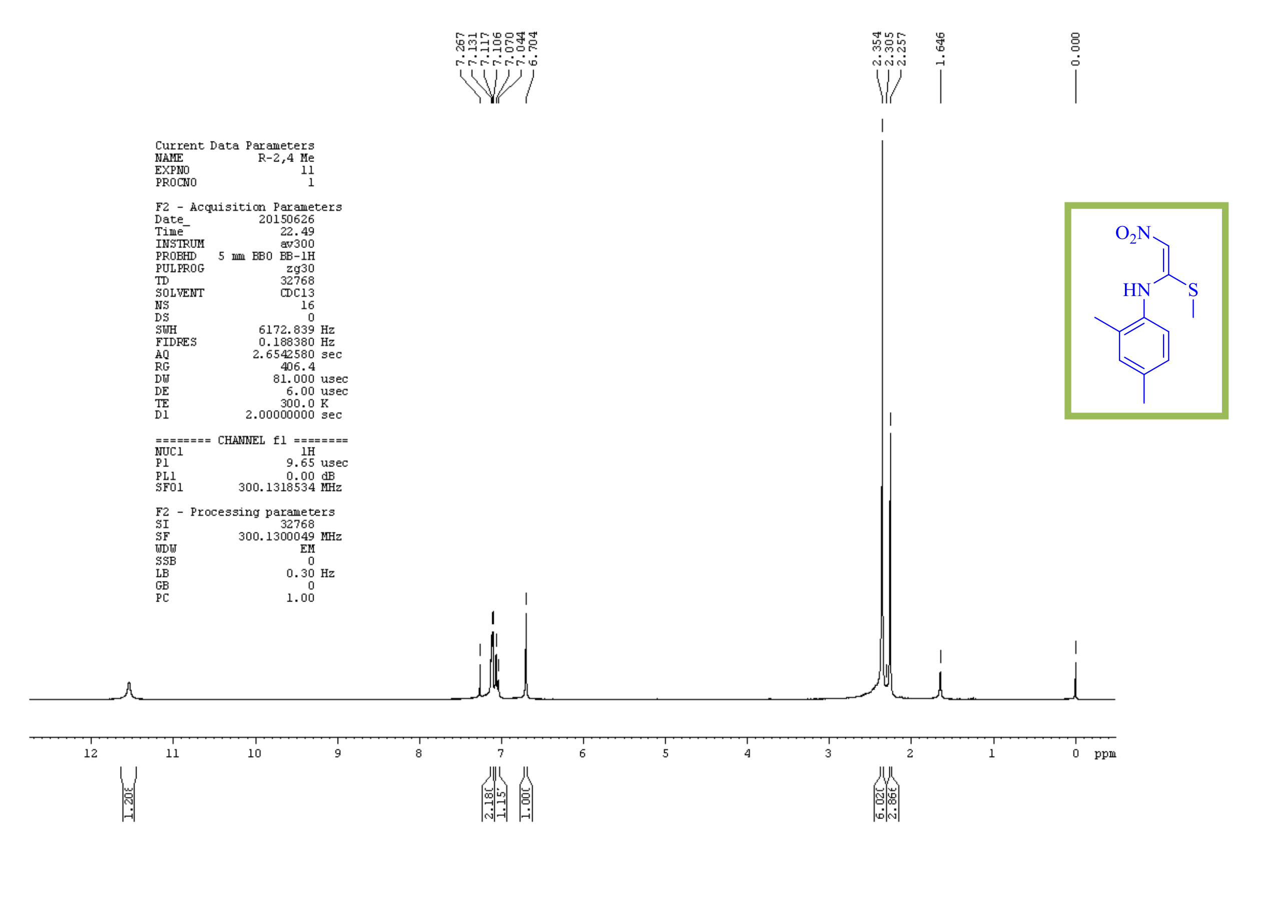

Figure 10 ^{1}H NMR Spectrum of **1q** ($CDCl_3$)

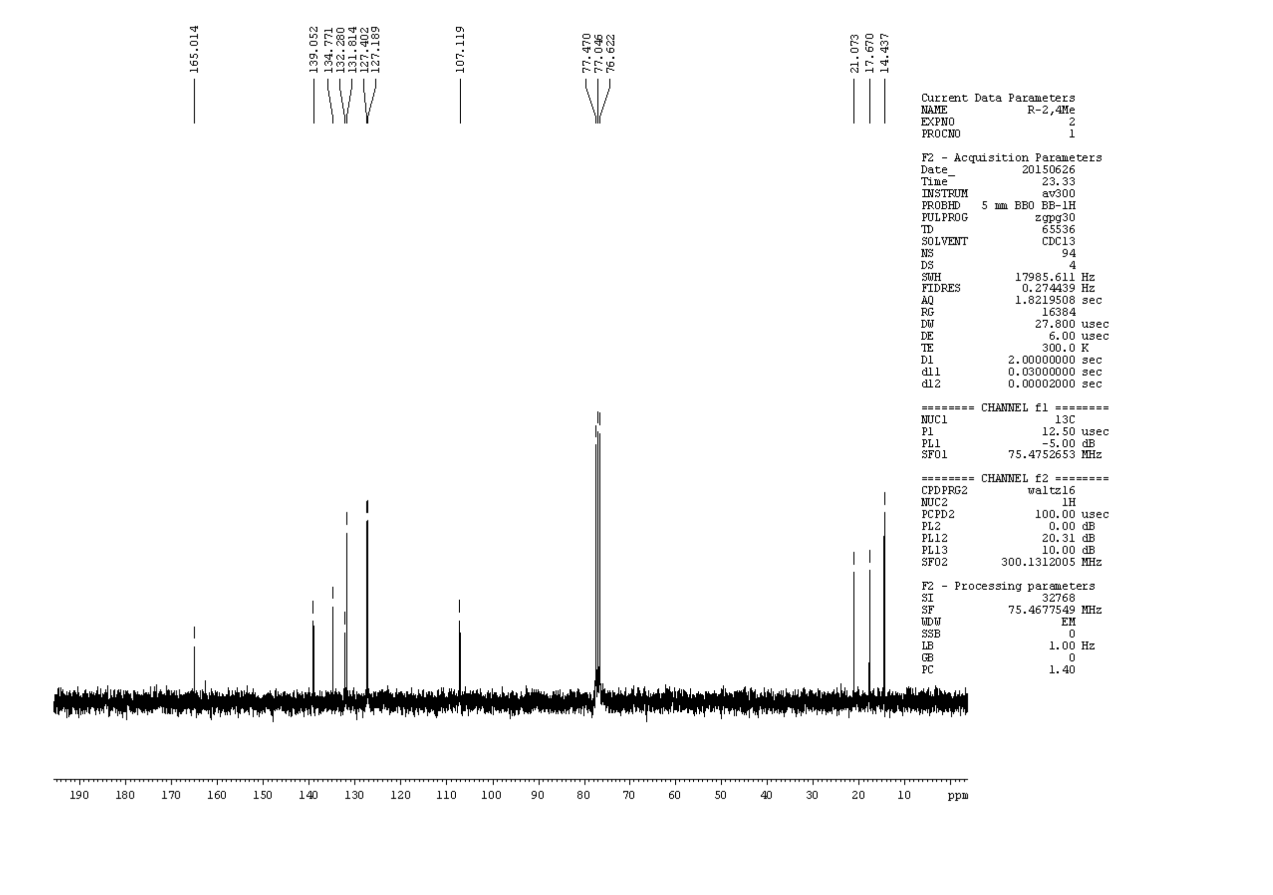

Figure 11 ^{13}C NMR Spectrum of **1q** ($CDCl_3$)

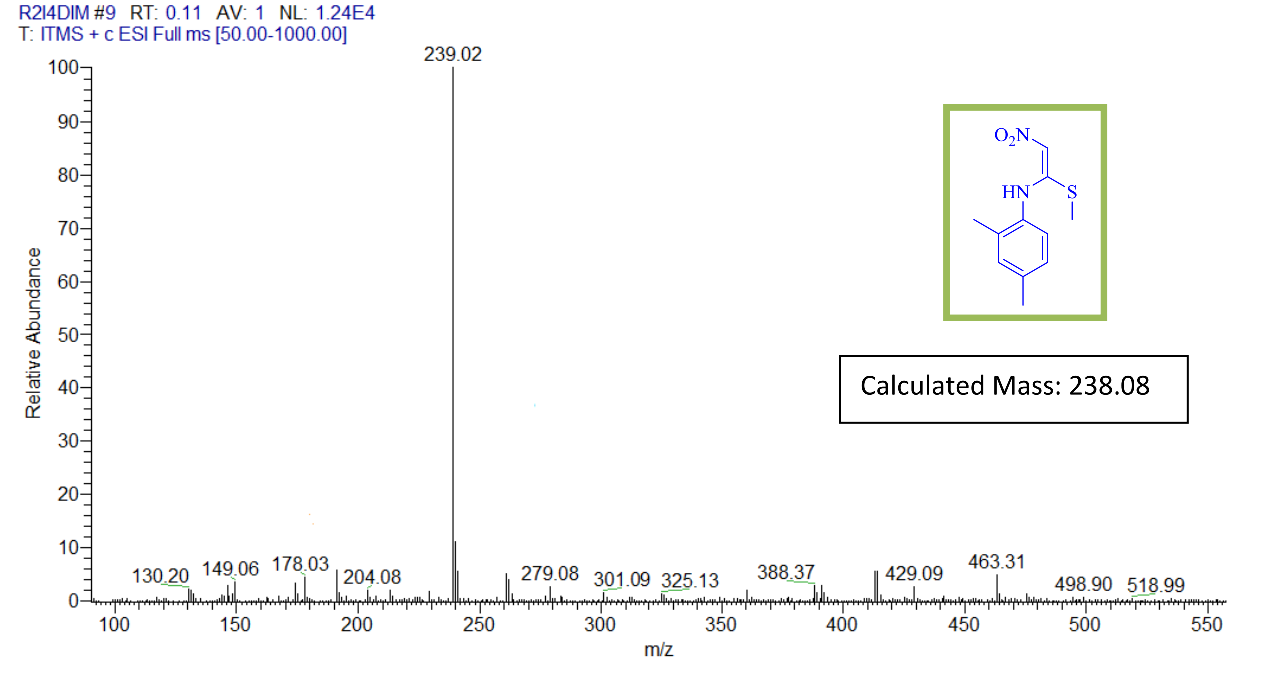

Figure 12 ESI mass spectrum of **1q**

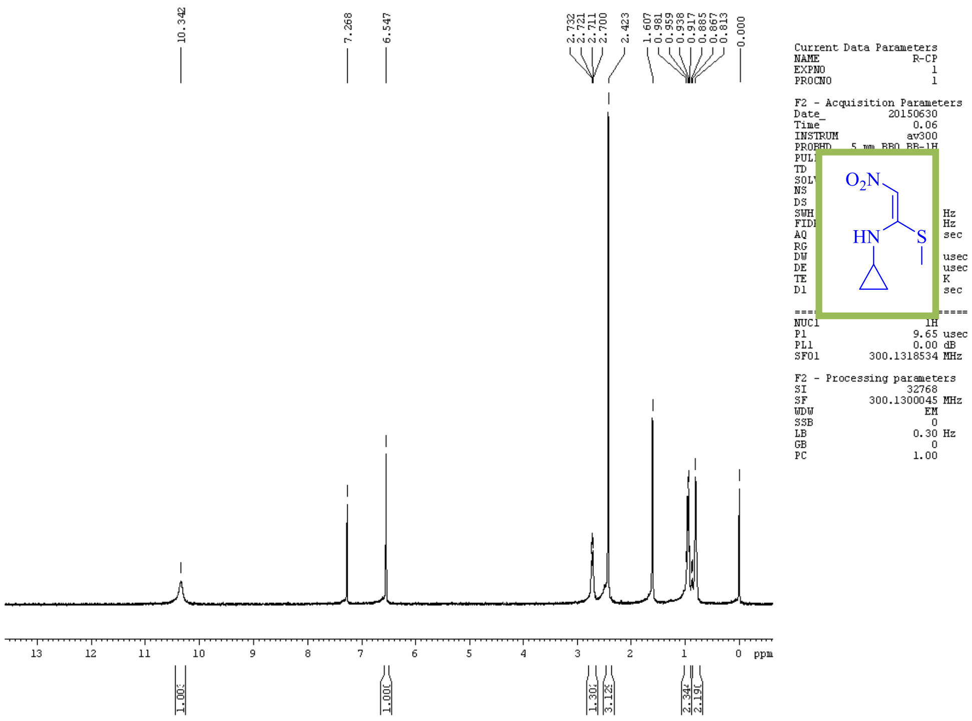

Figure 13 ^{1}H NMR Spectrum of **1w** ($CDCl_3$)

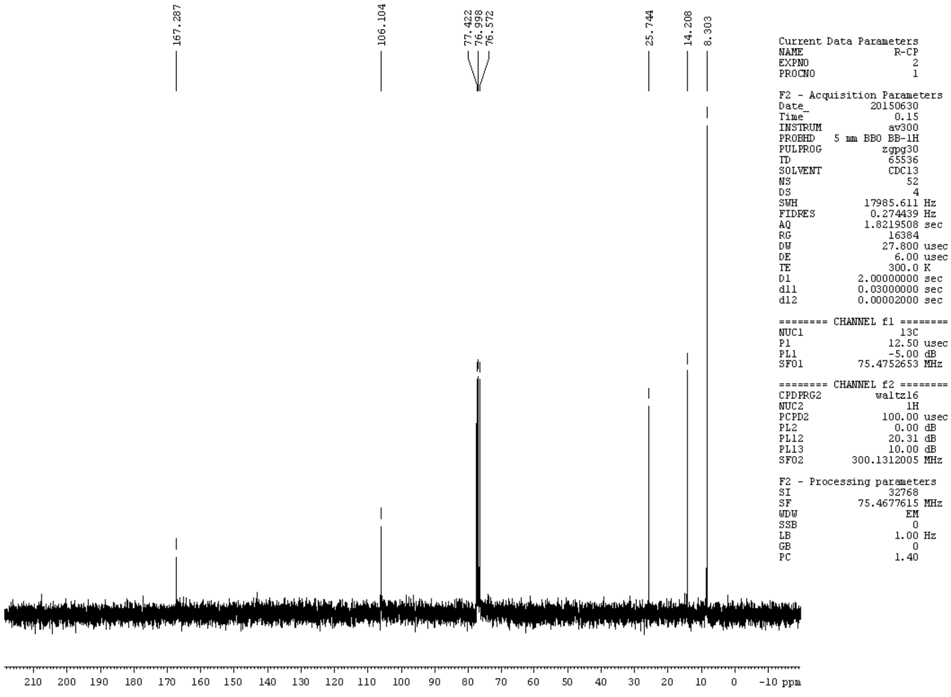

Figure 14 ^{13}C NMR Spectrum of **1w** ($CDCl_3$)

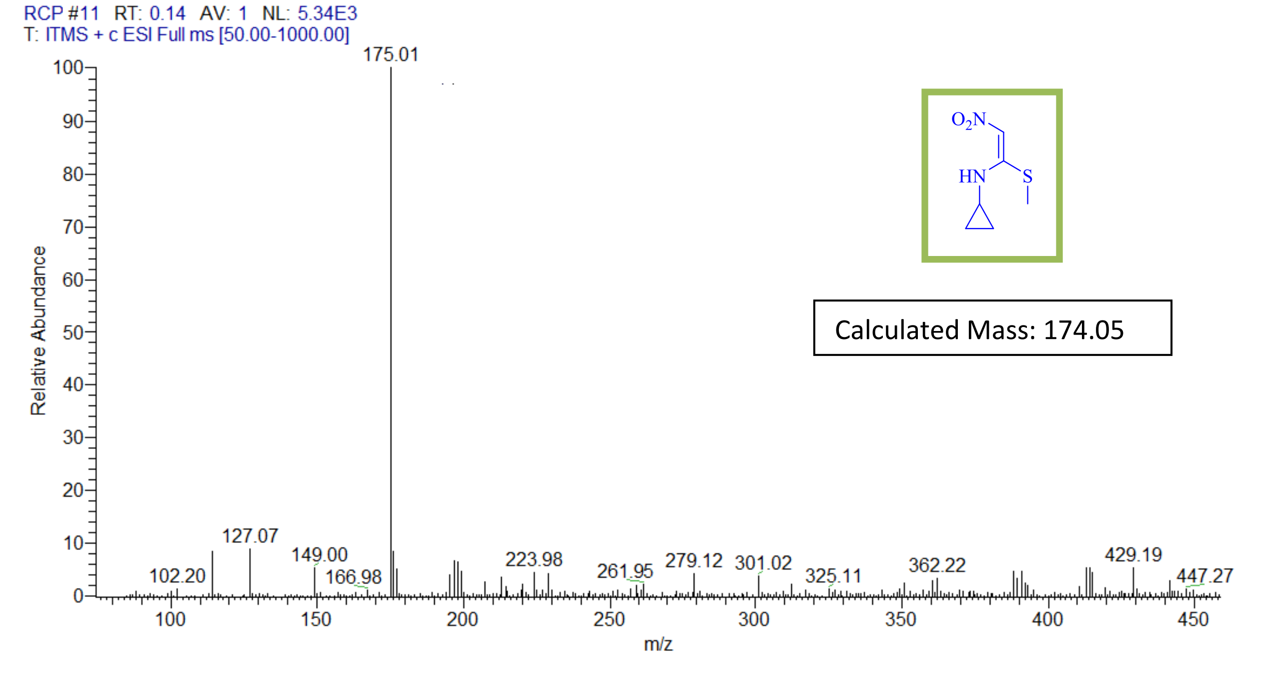

Figure 15 ESI mass spectrum of **1w**

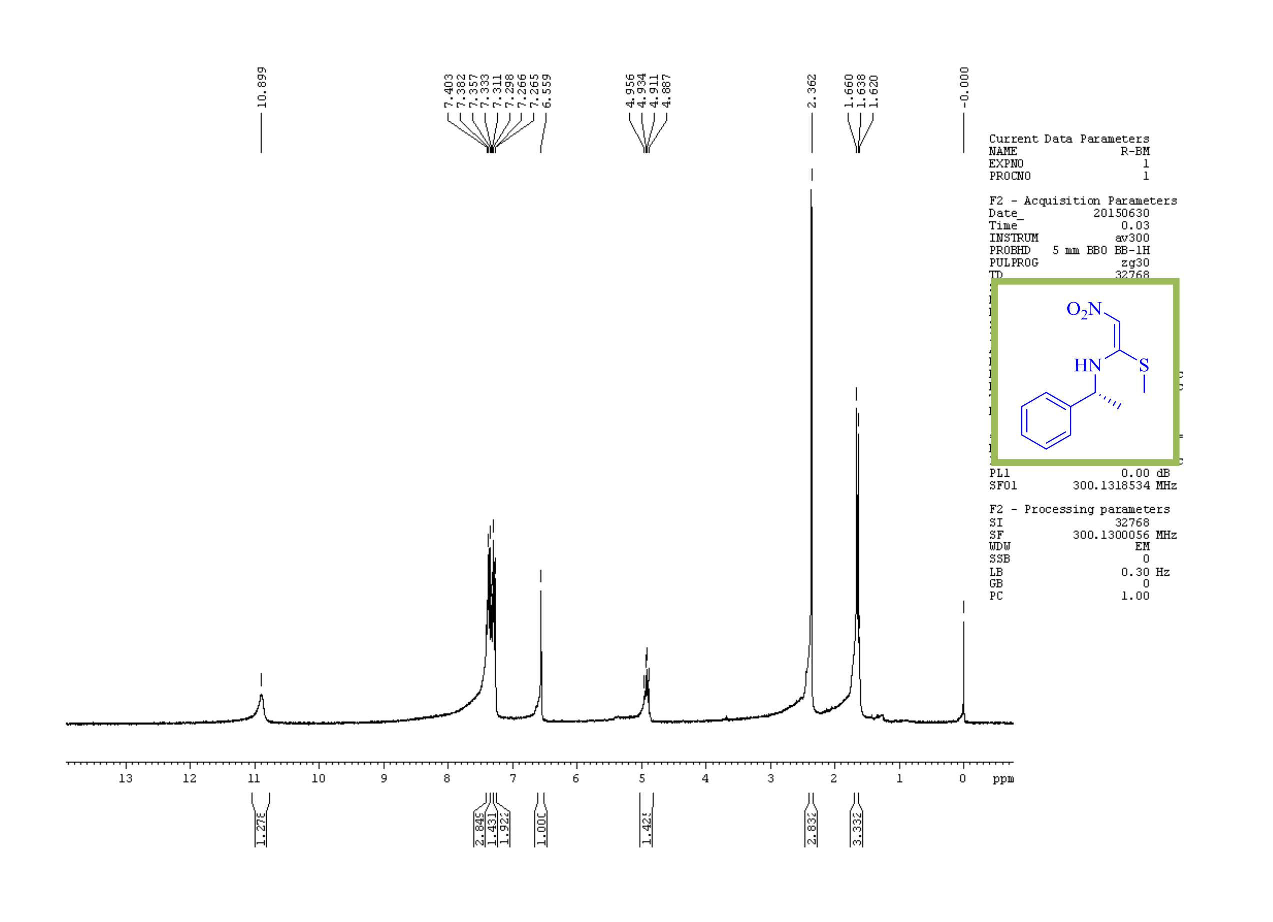

Figure 16 ^{1}H NMR Spectrum of **1z** ($CDCl_3$)

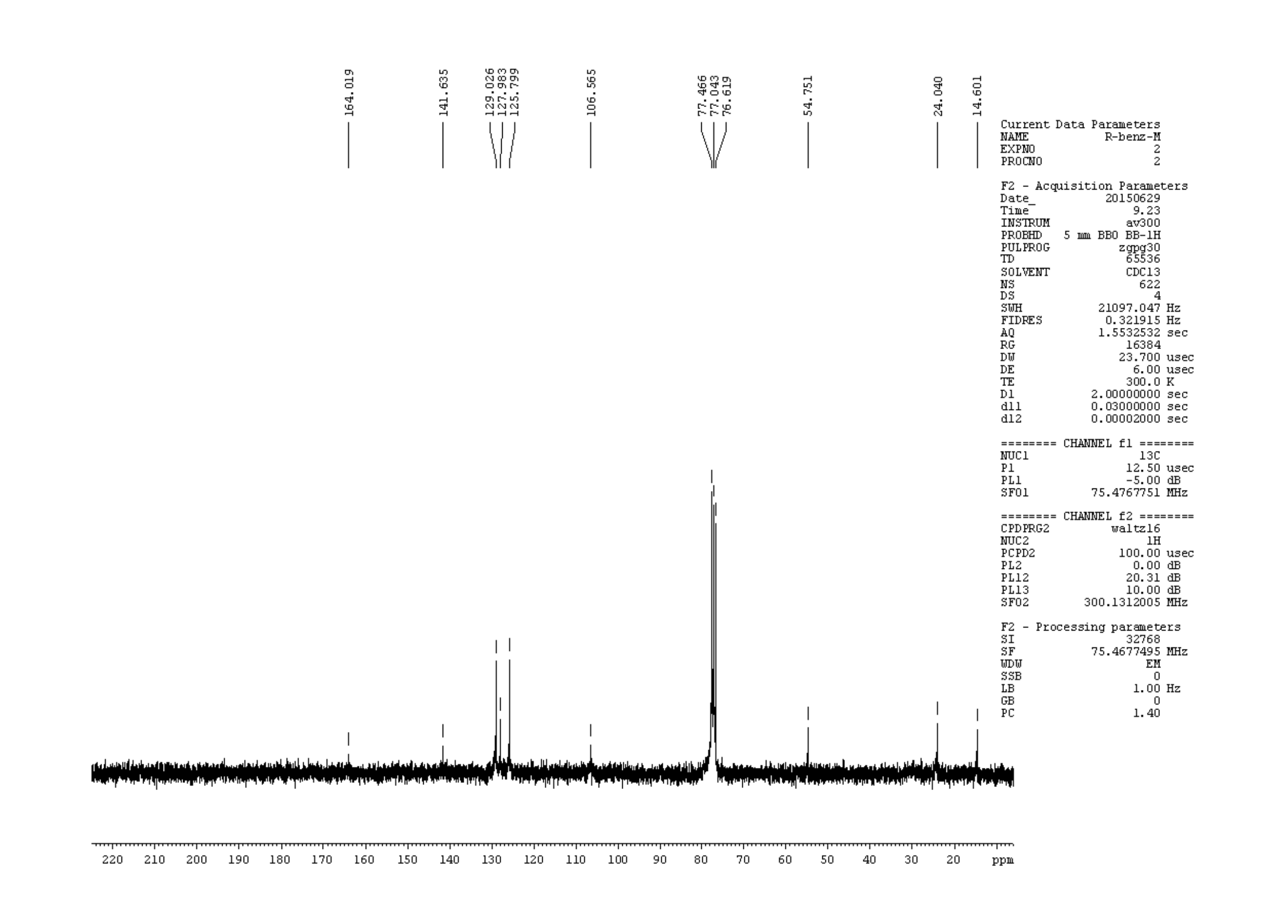

Figure 17 ^{13}C NMR Spectrum of **1z** ($CDCl_3$)

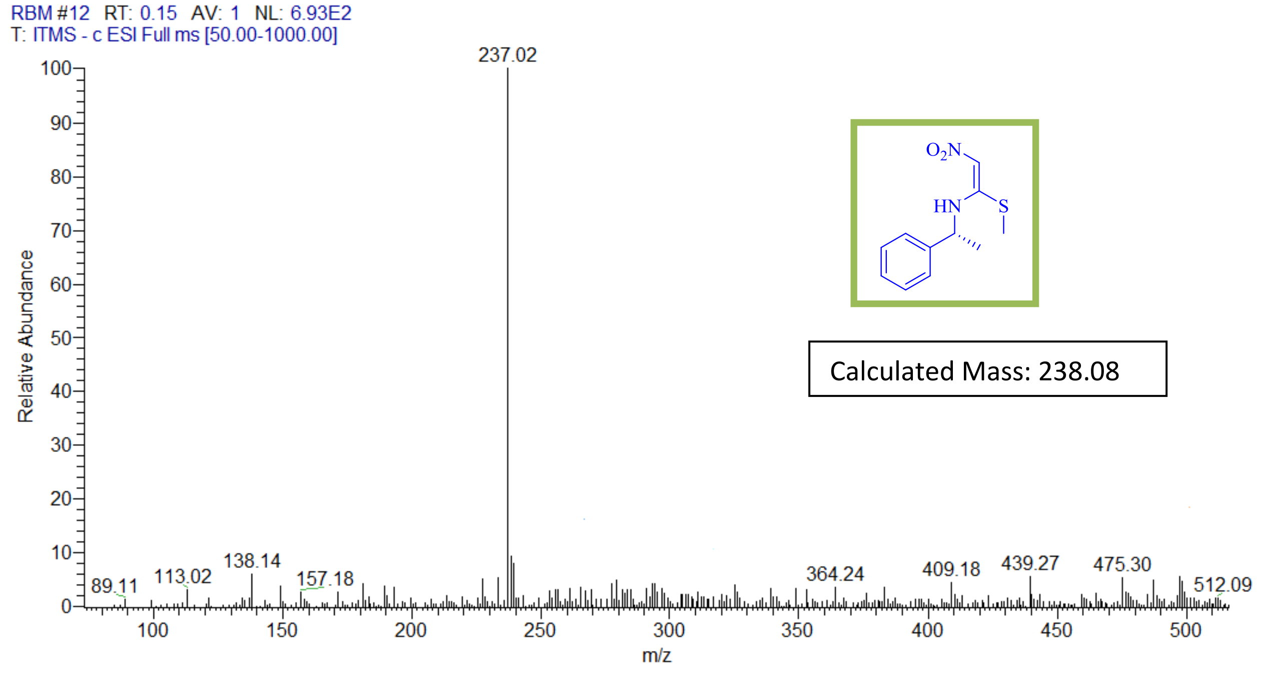

Figure 18 ESI mass spectrum of Spectrum of **1z**

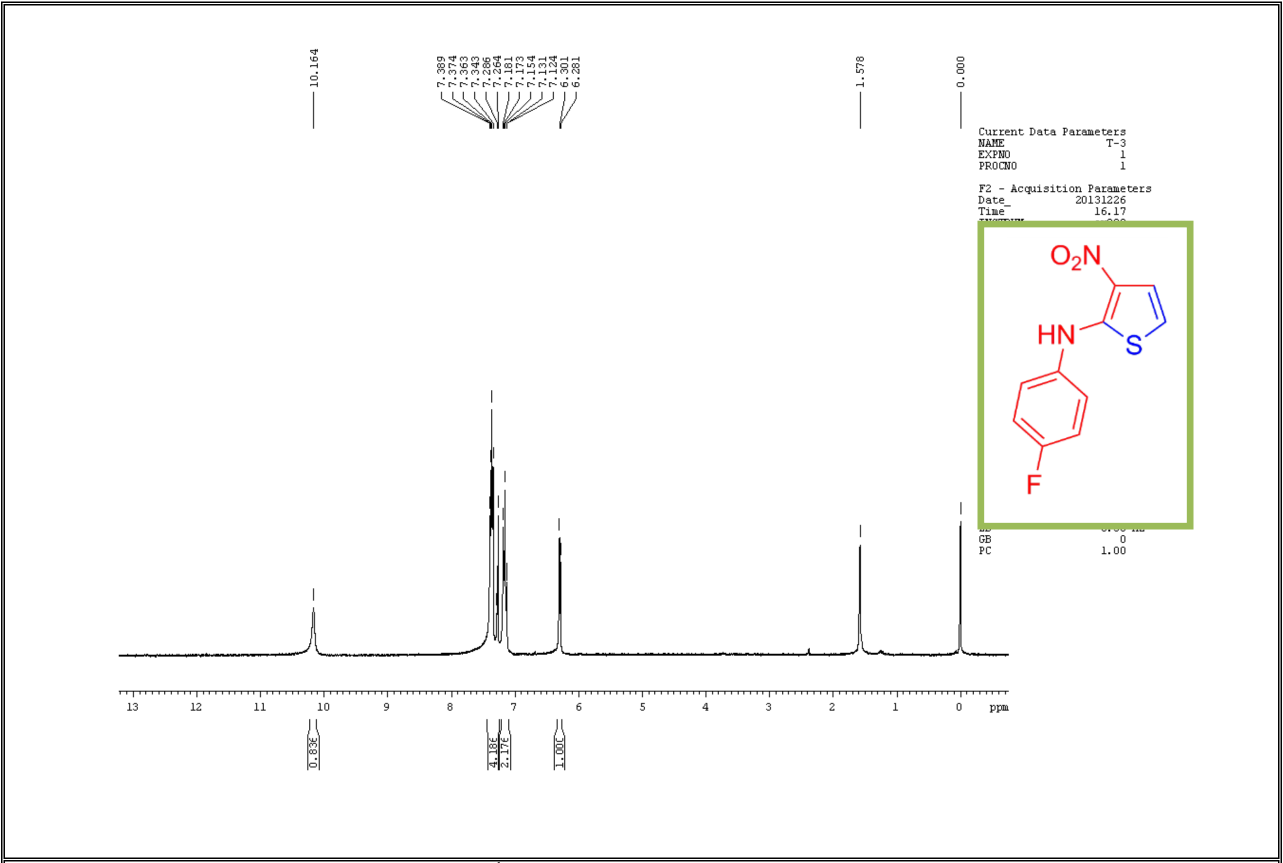

Figure 19 ^{1}H NMR Spectrum of **3a** ($CDCl_3$)

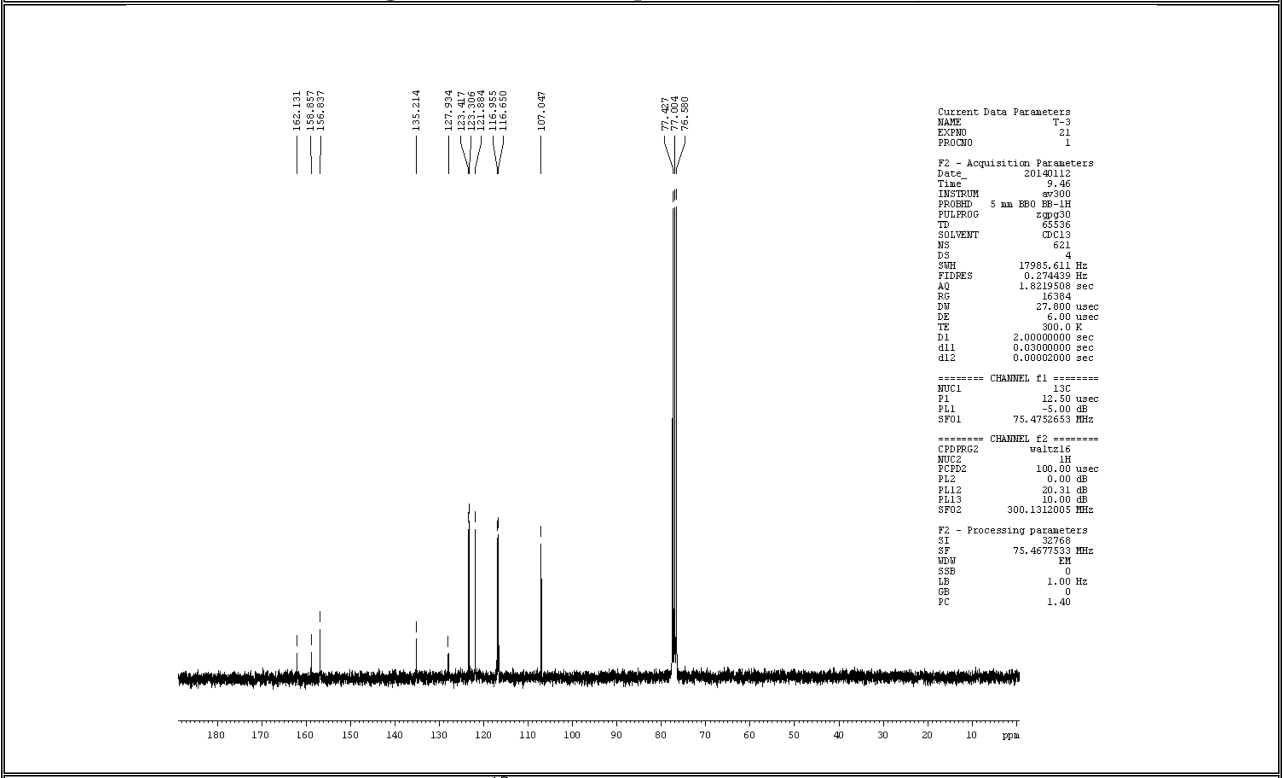

Figure 20 ^{13}C NMR Spectrum of **3a** ($CDCl_3$)

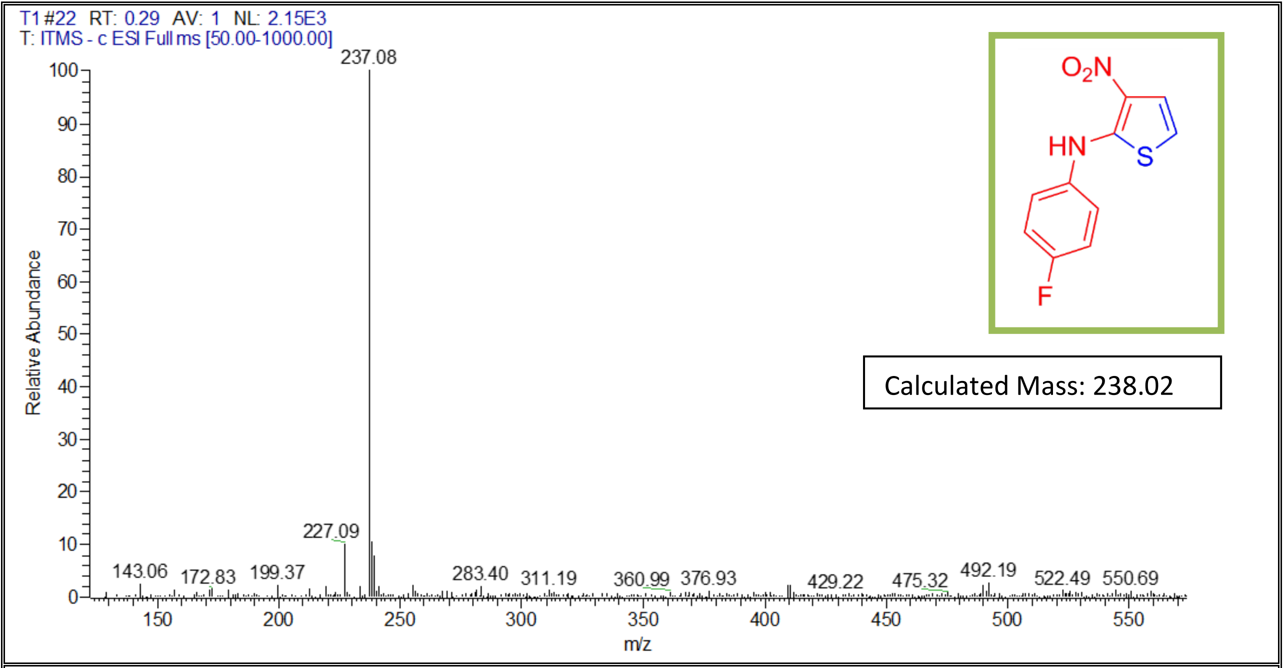

Figure 21 ESI mass spectrum of Spectrum of **3a**

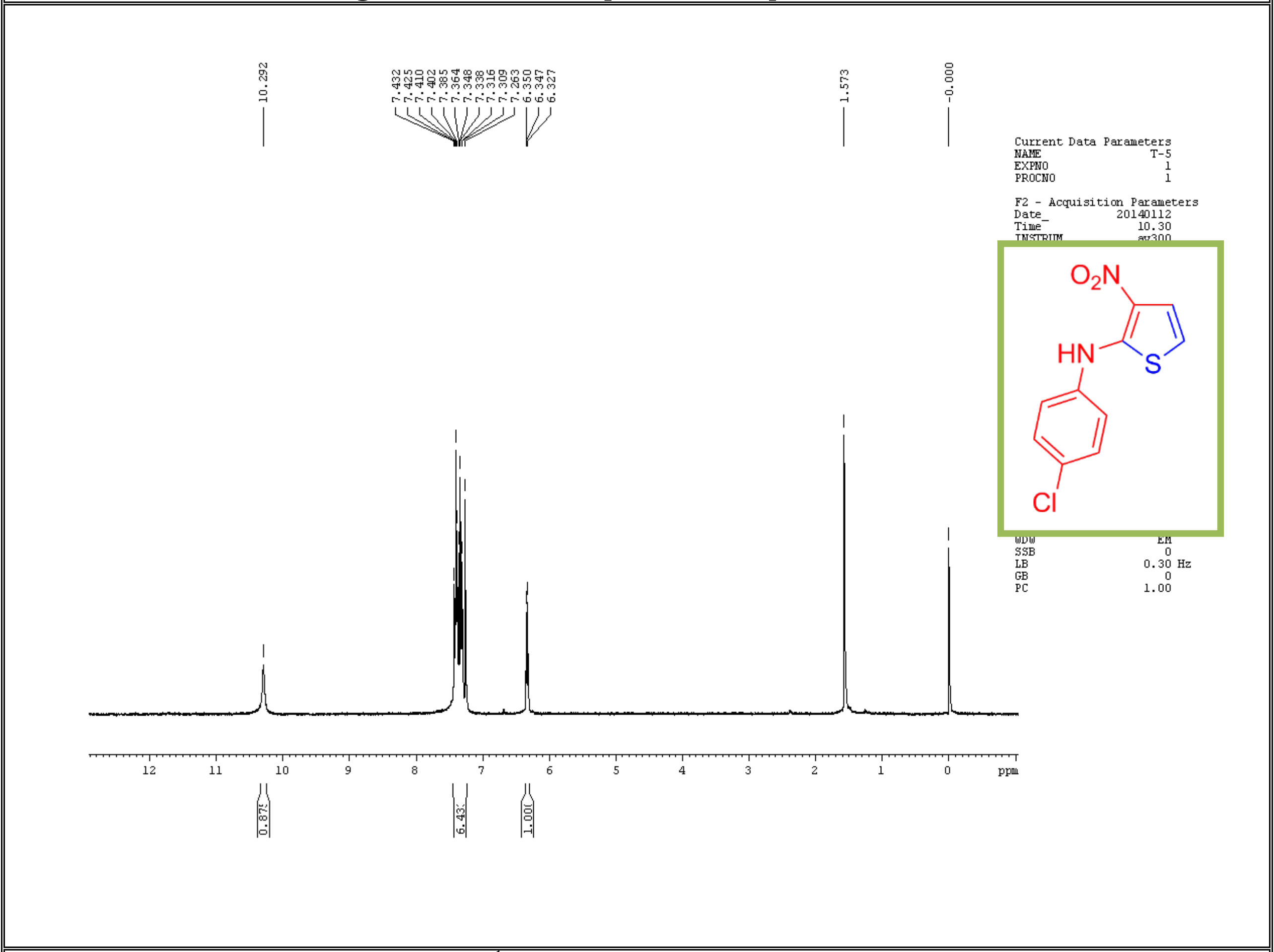

Figure 22 ^{1}H NMR Spectrum of **3b**($CDCl_3$)

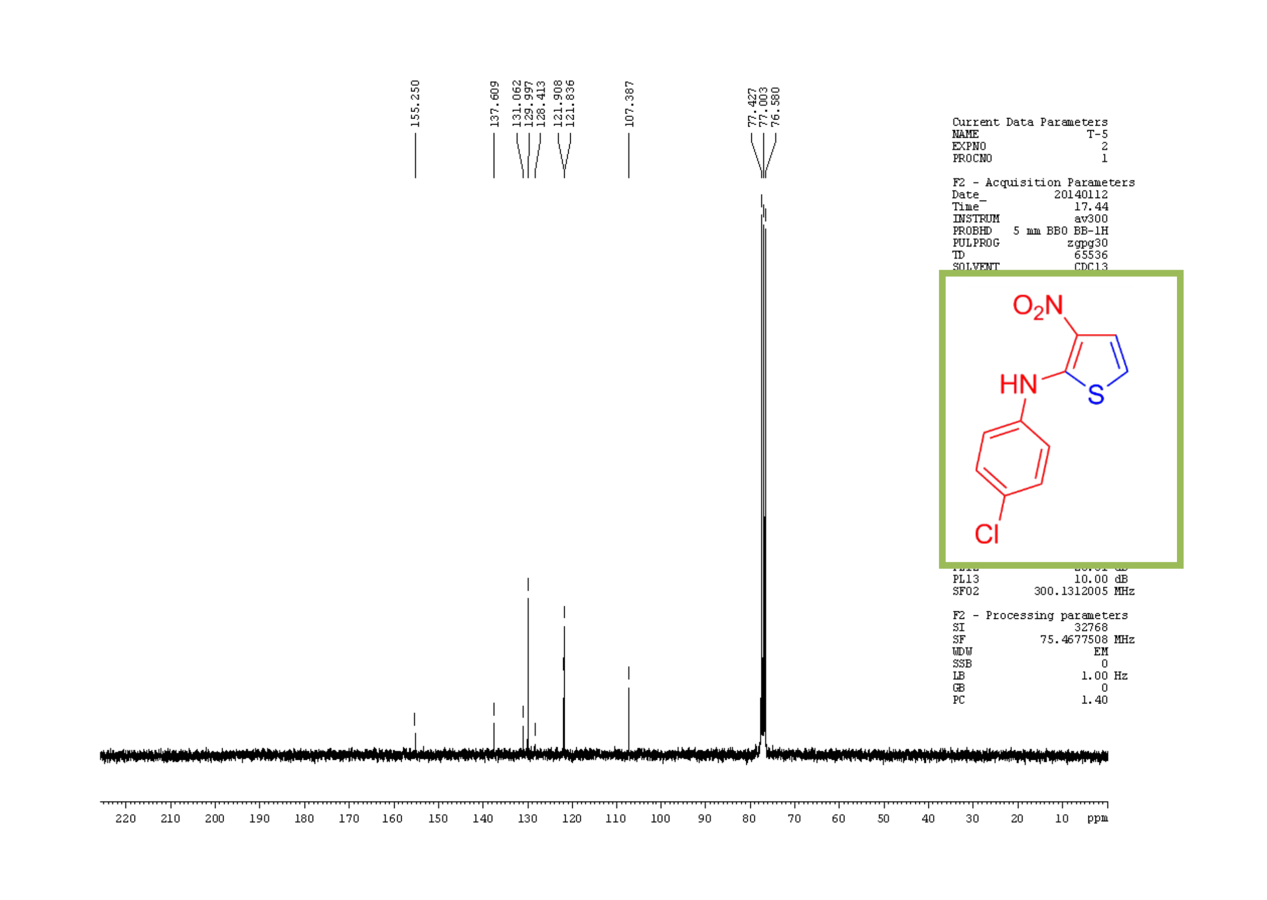

Figure 23 ^{13}C NMR Spectrum of **3b**($CDCl_3$)

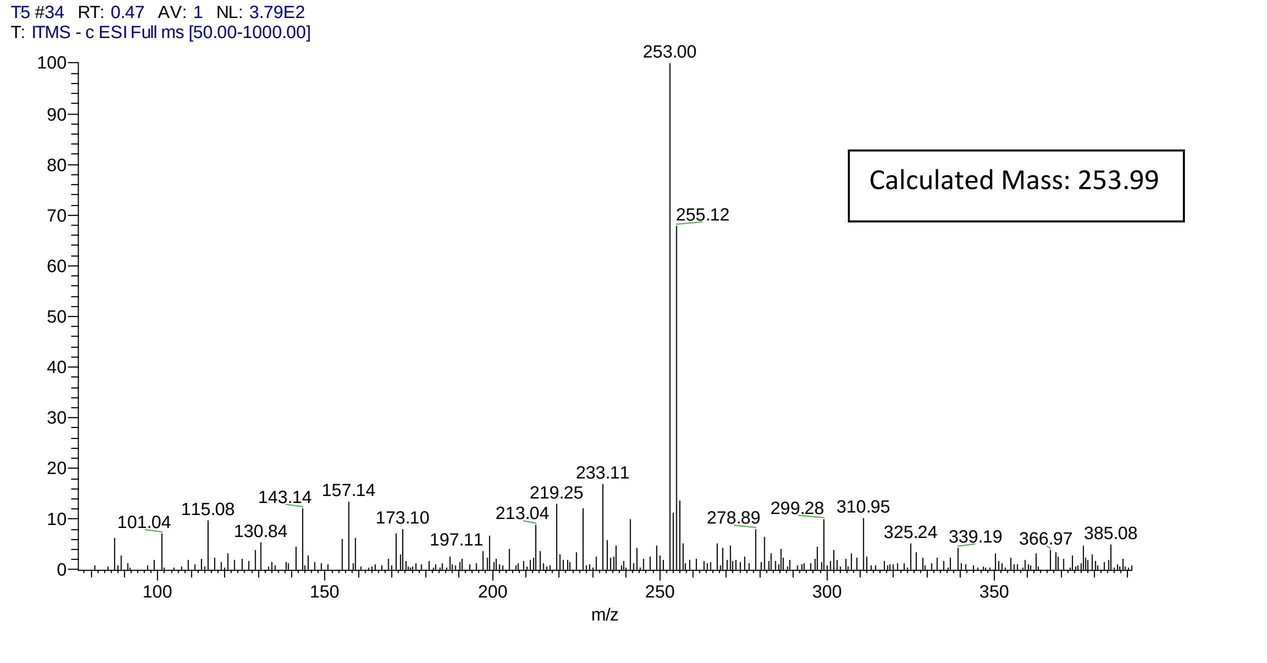

Figure 24 ESI mass spectrum of Spectrum of **3b**

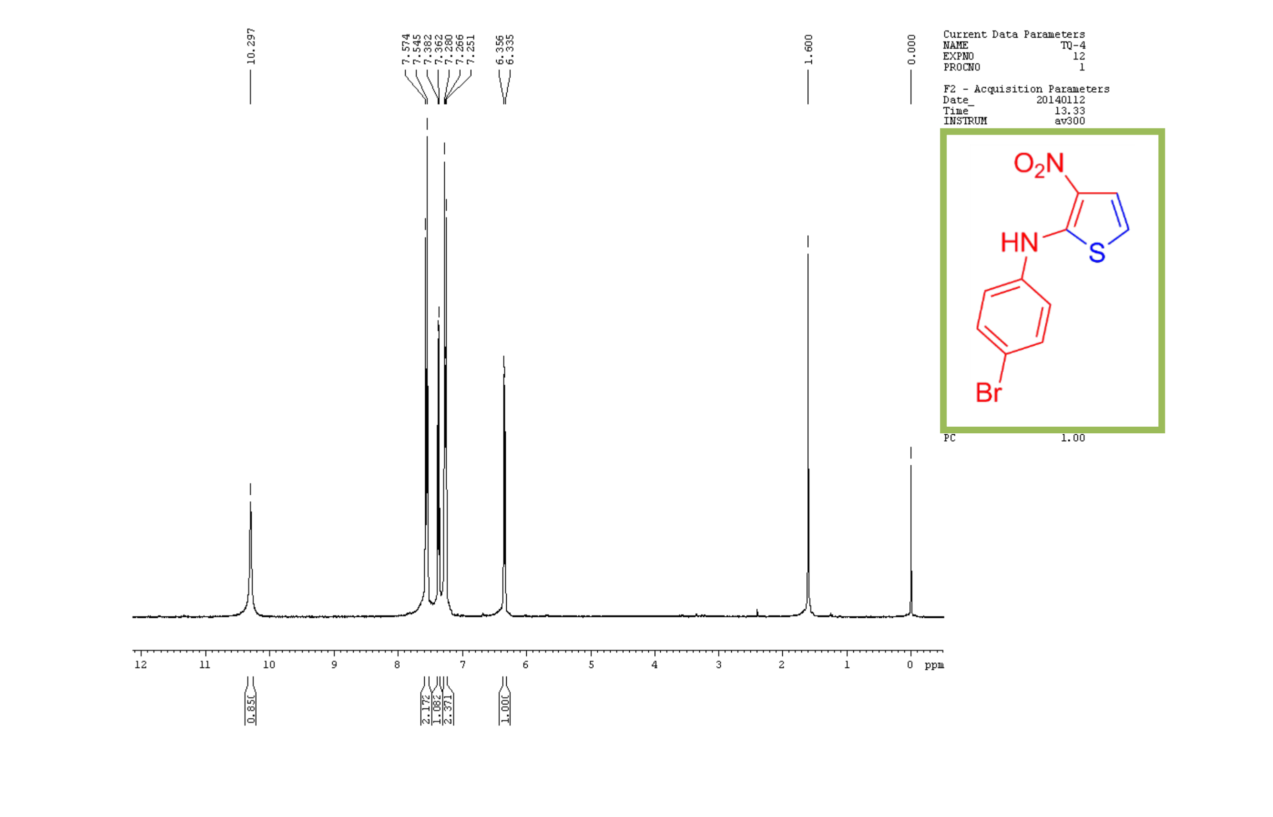

Figure 25 ^{1}H NMR Spectrum of **3c**($CDCl_3$)

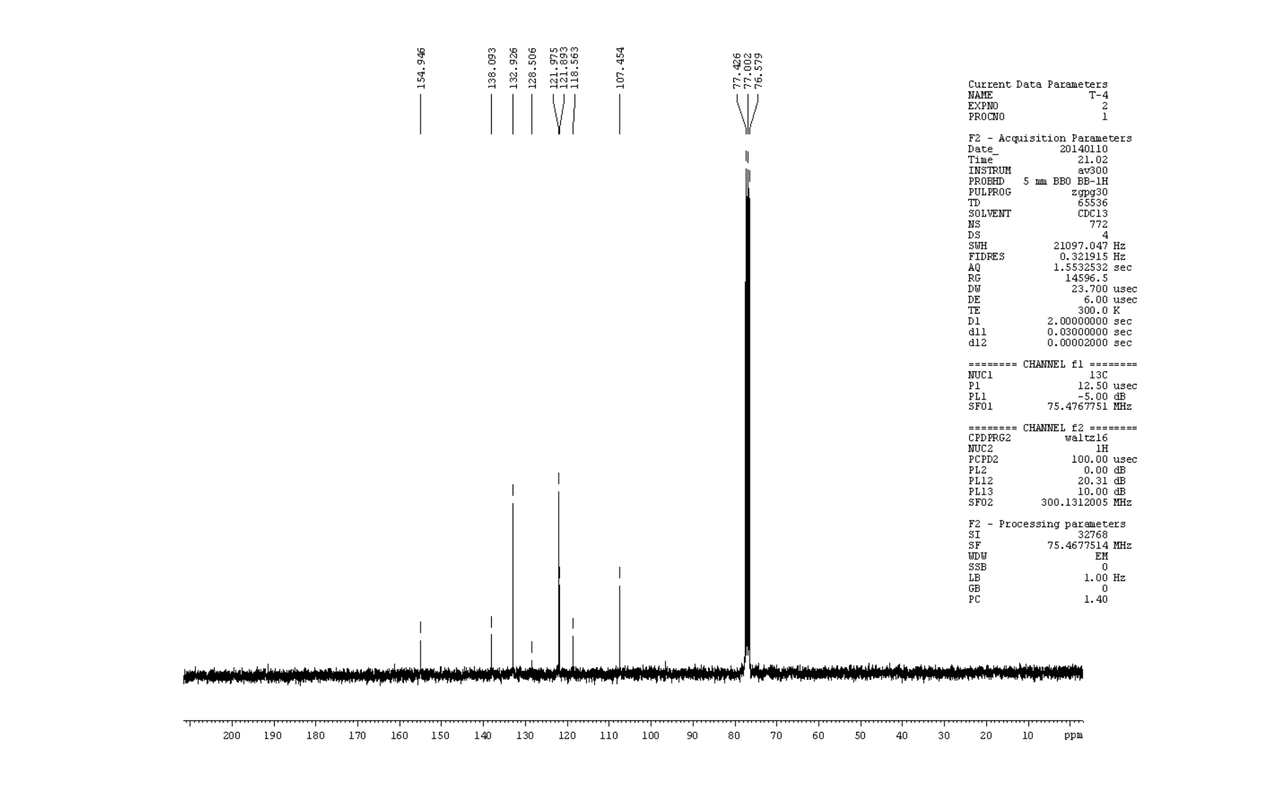

Figure 26 ^{13}C NMR Spectrum of **3c**($CDCl_3$)

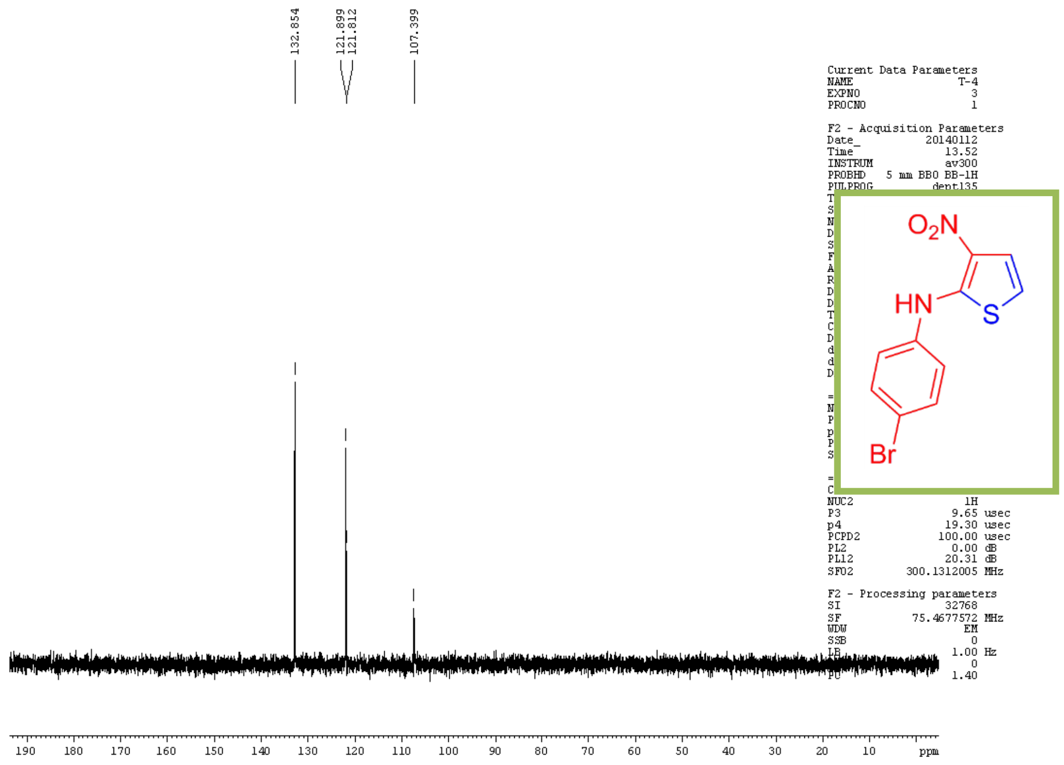

Figure 27 DEPT Spectrum of **3c** ($CDCl_3$)

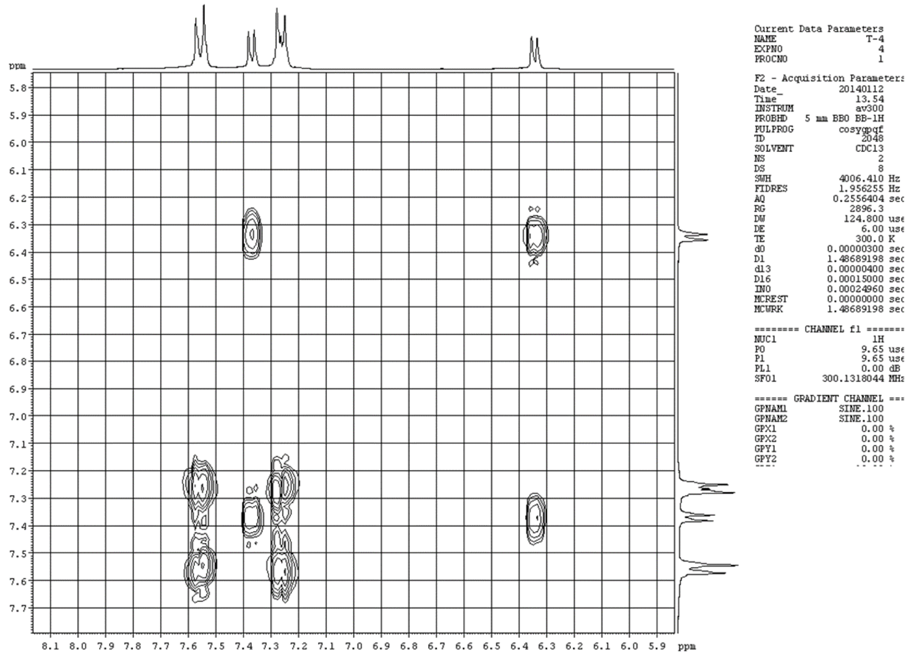

Figure 28 H, H-COSY Spectrum of **3c** ($CDCl_3$)

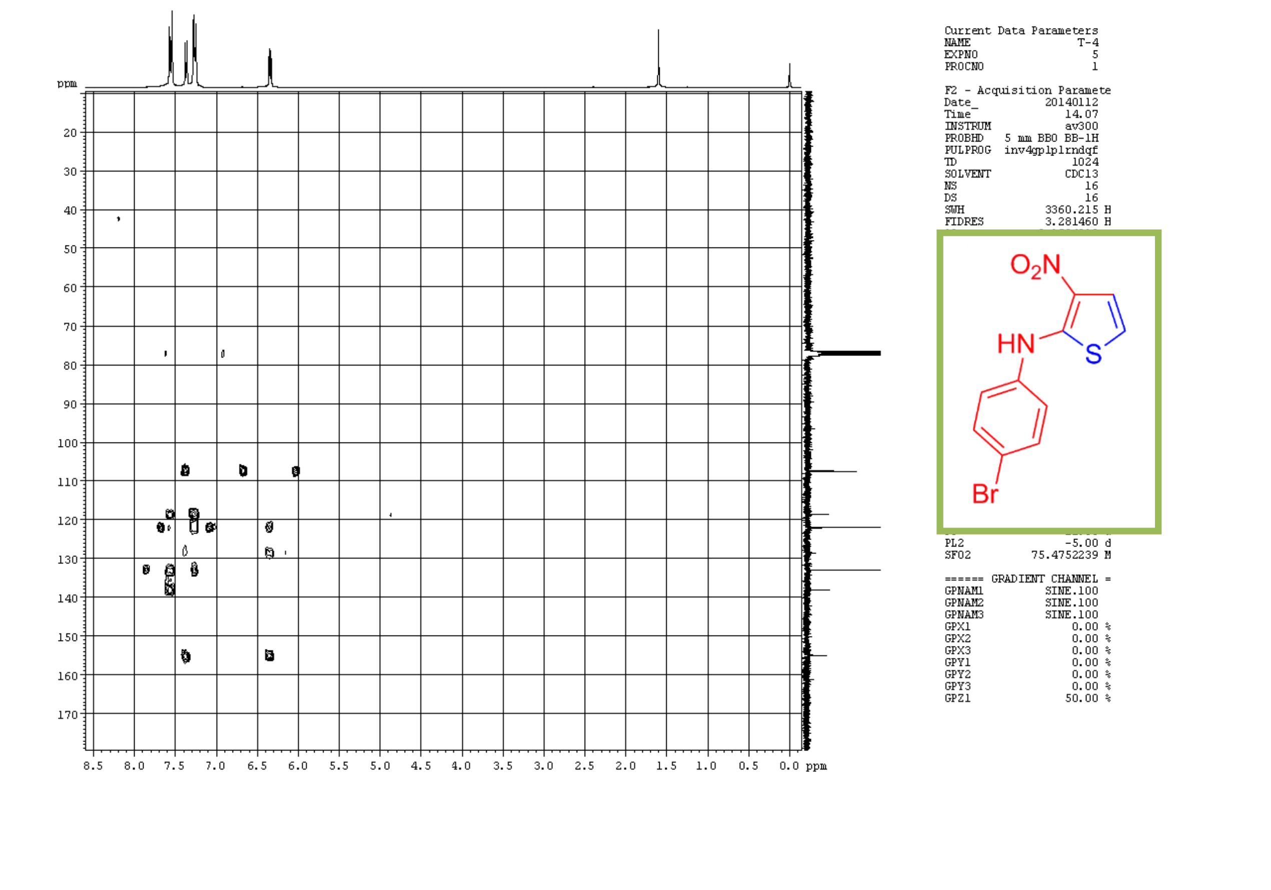

Figure 29 HMBC Spectrum of **3c** ($CDCl_3$)

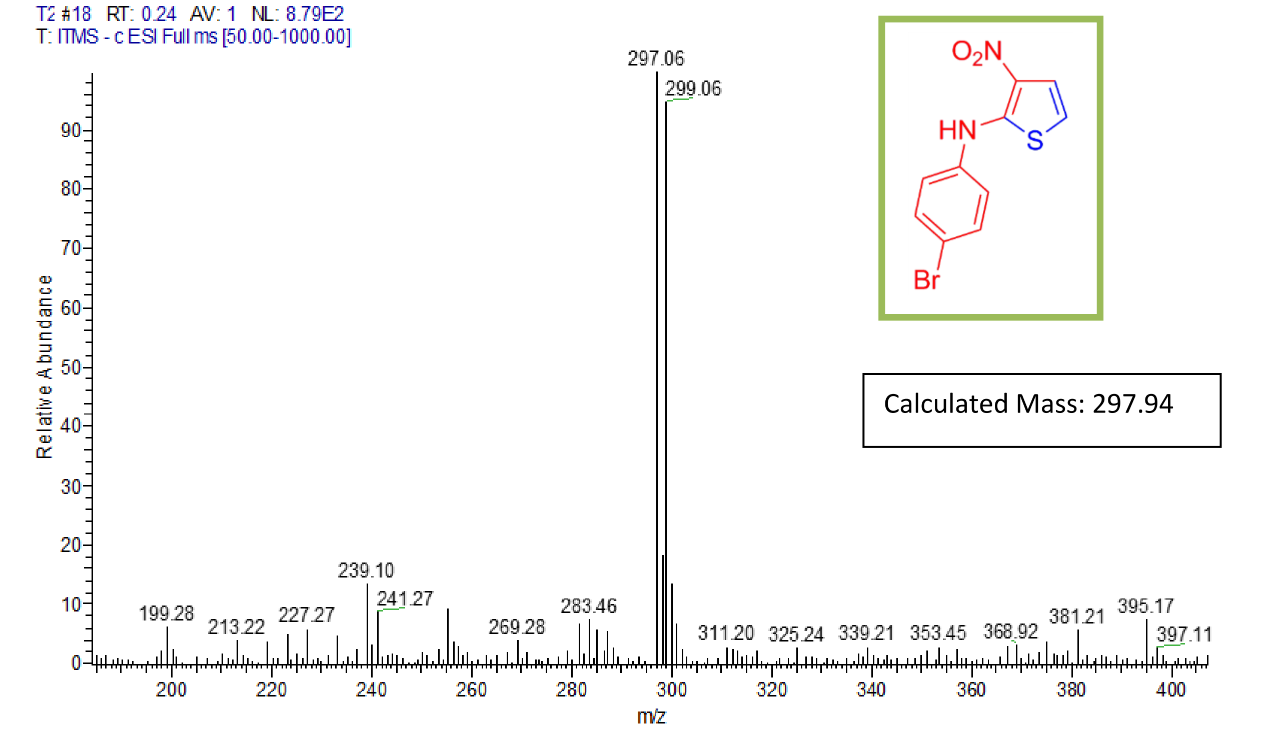

Figure 30 ESI mass spectrum of Spectrum of **3c**

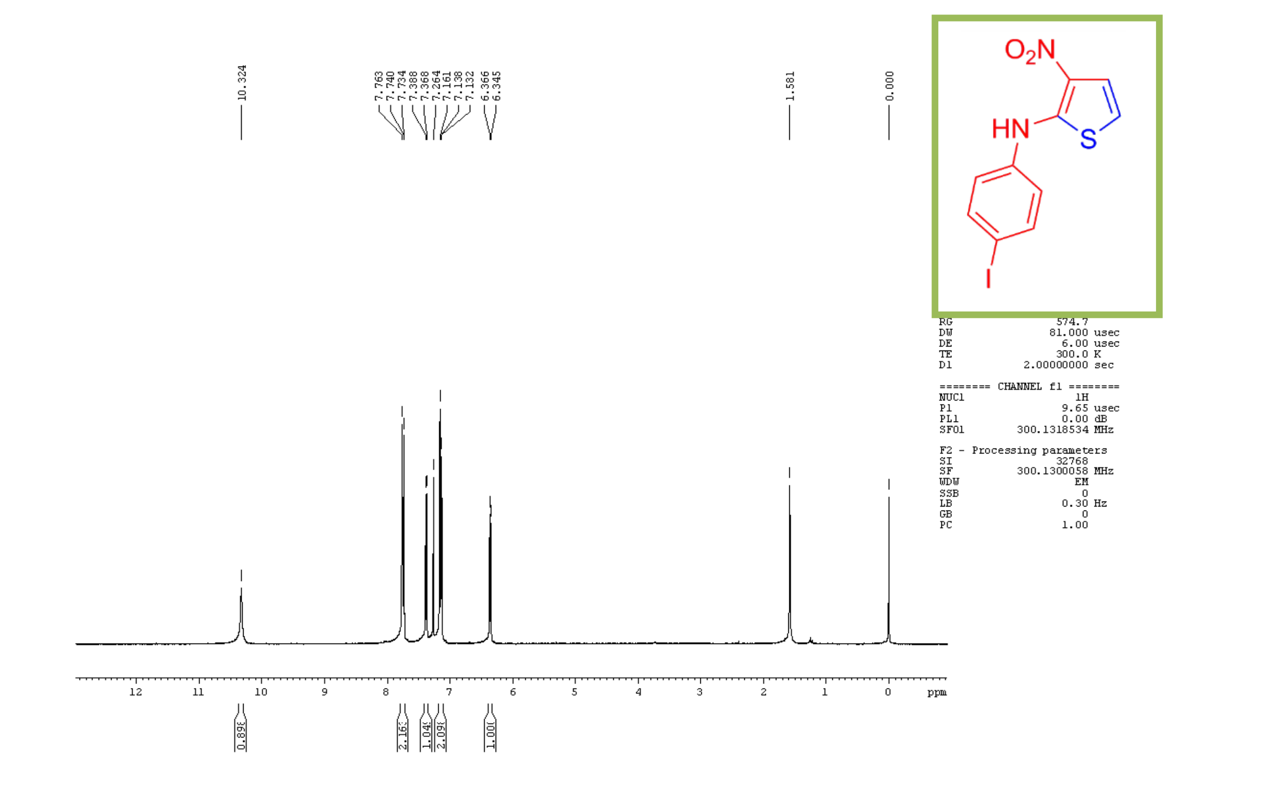

Figure 31 ¹H NMR Spectrum **3d**($CDCl_3$)

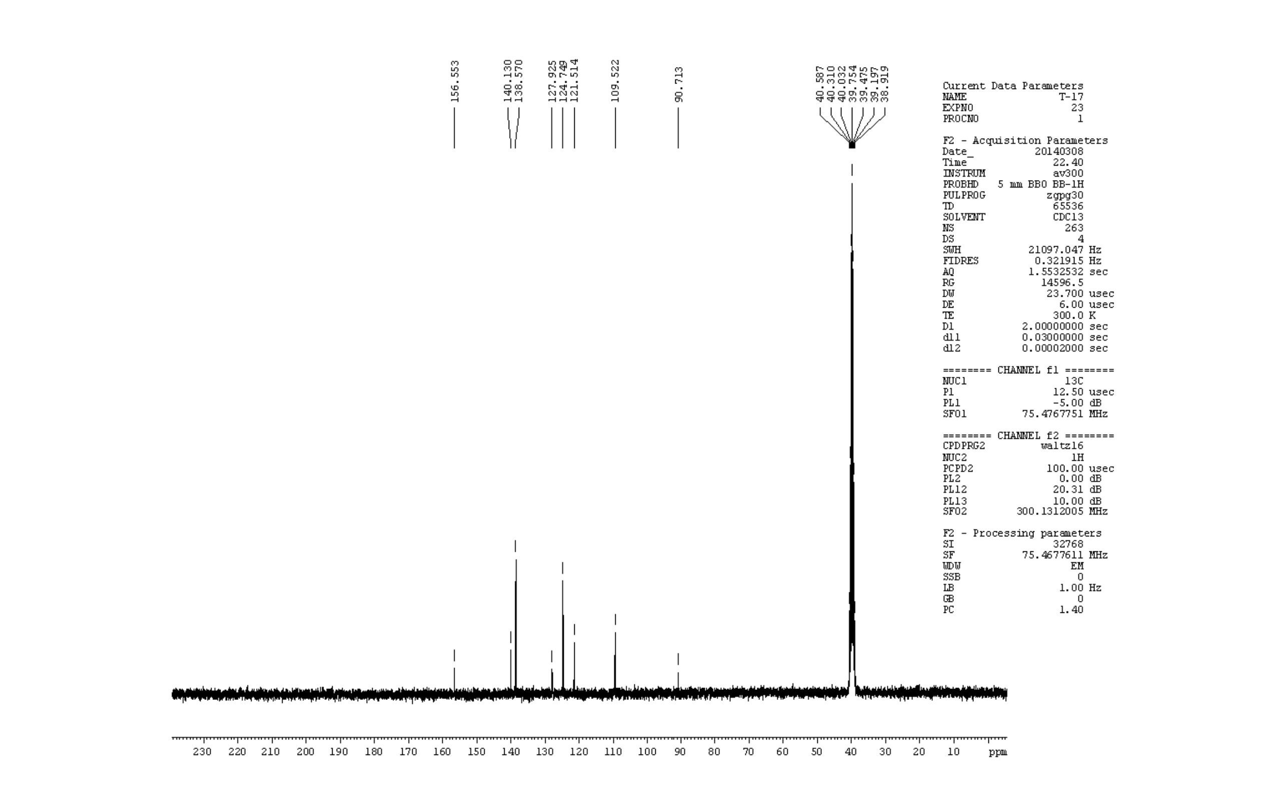

Figure 32 ¹³C NMR Spectrum of **3d** (DMSO-d⁶)

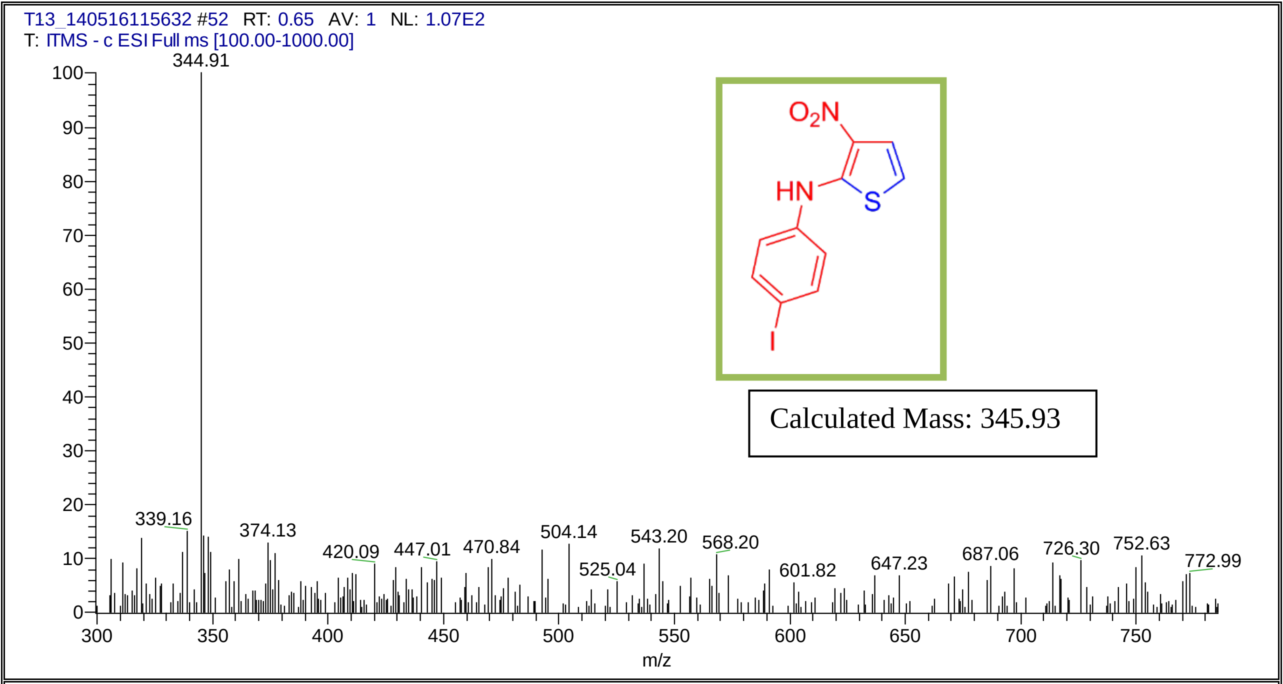

Figure 33 ESI mass spectrum of **3d**

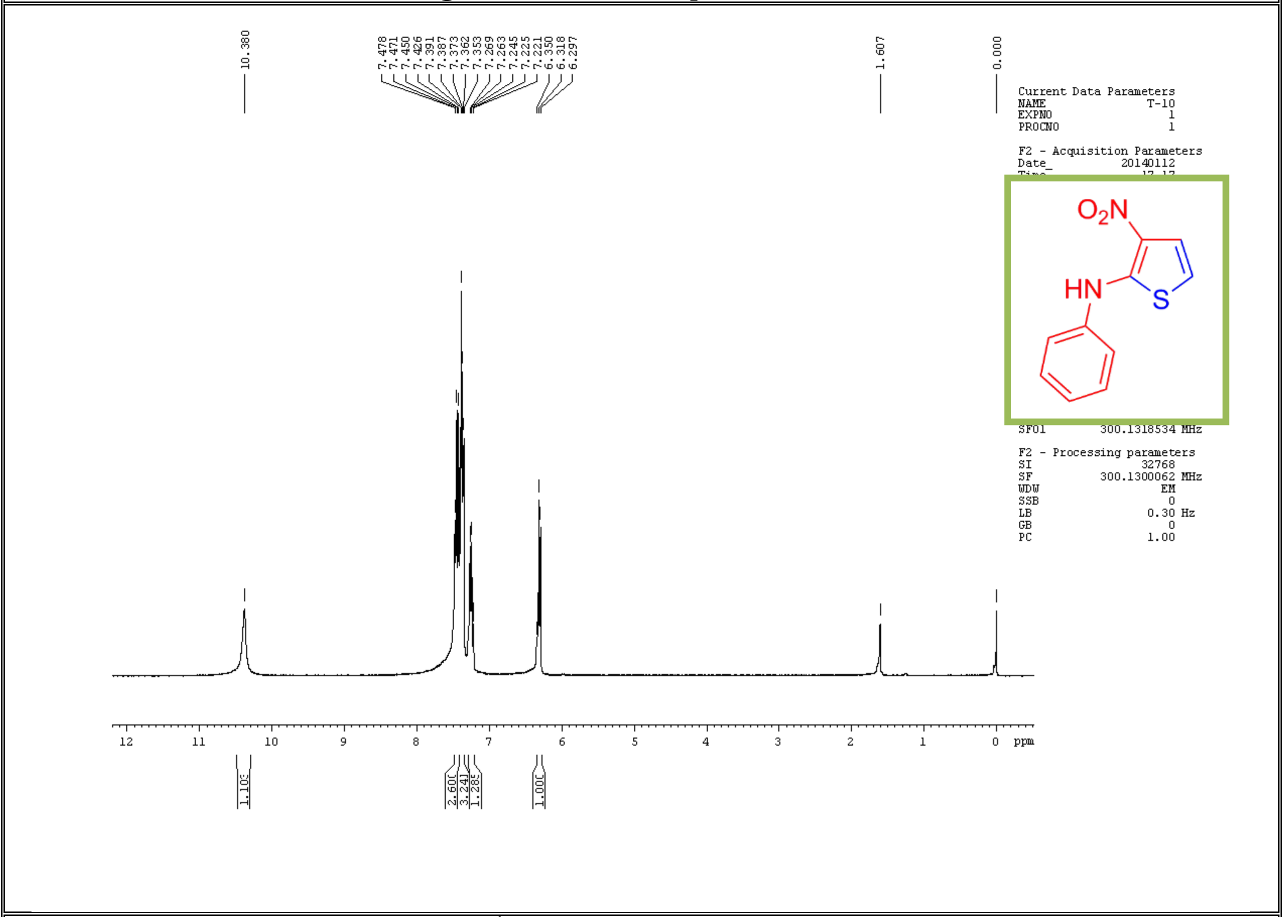

Figure 34 ¹H NMR Spectrum of **3e** ($CDCl_3$)

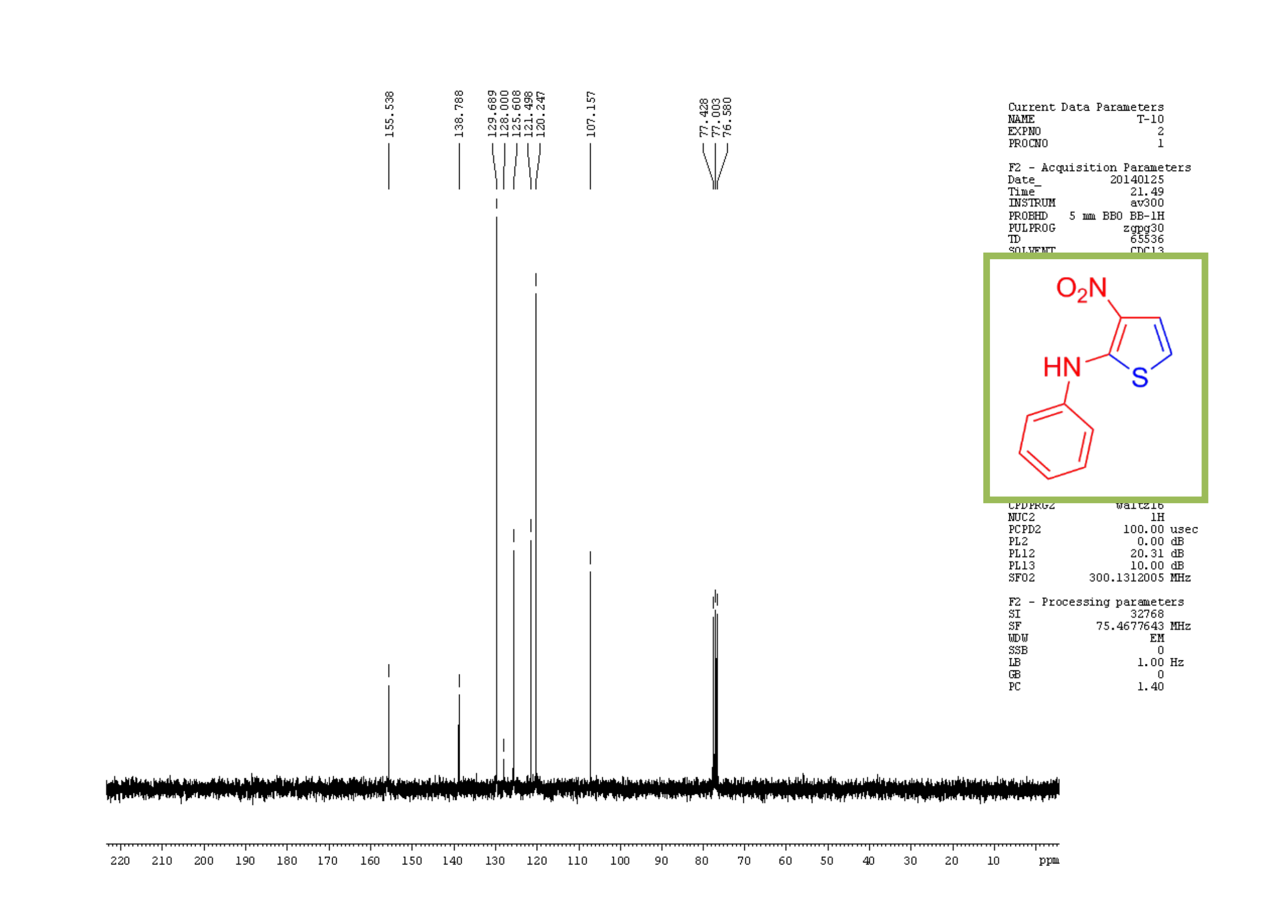

Figure 35 ^{13}C NMR Spectrum of **3e**($CDCl_3$)

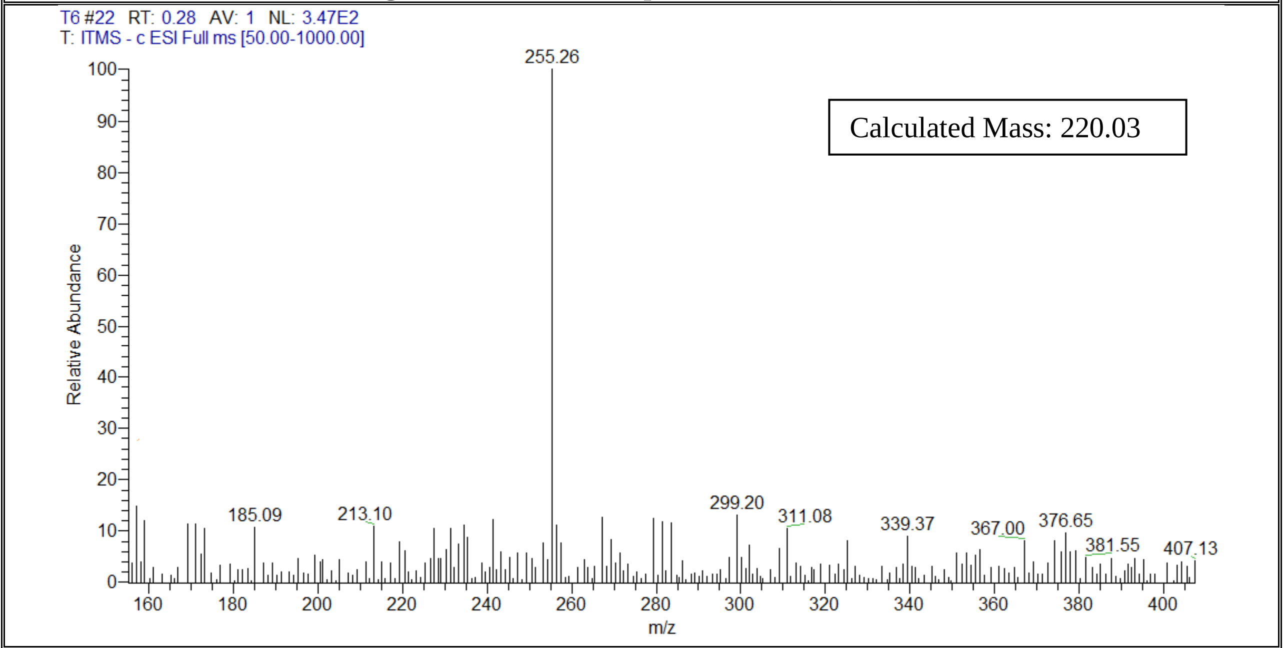

Figure 36 ESI mass spectrum of Spectrum of **3e**

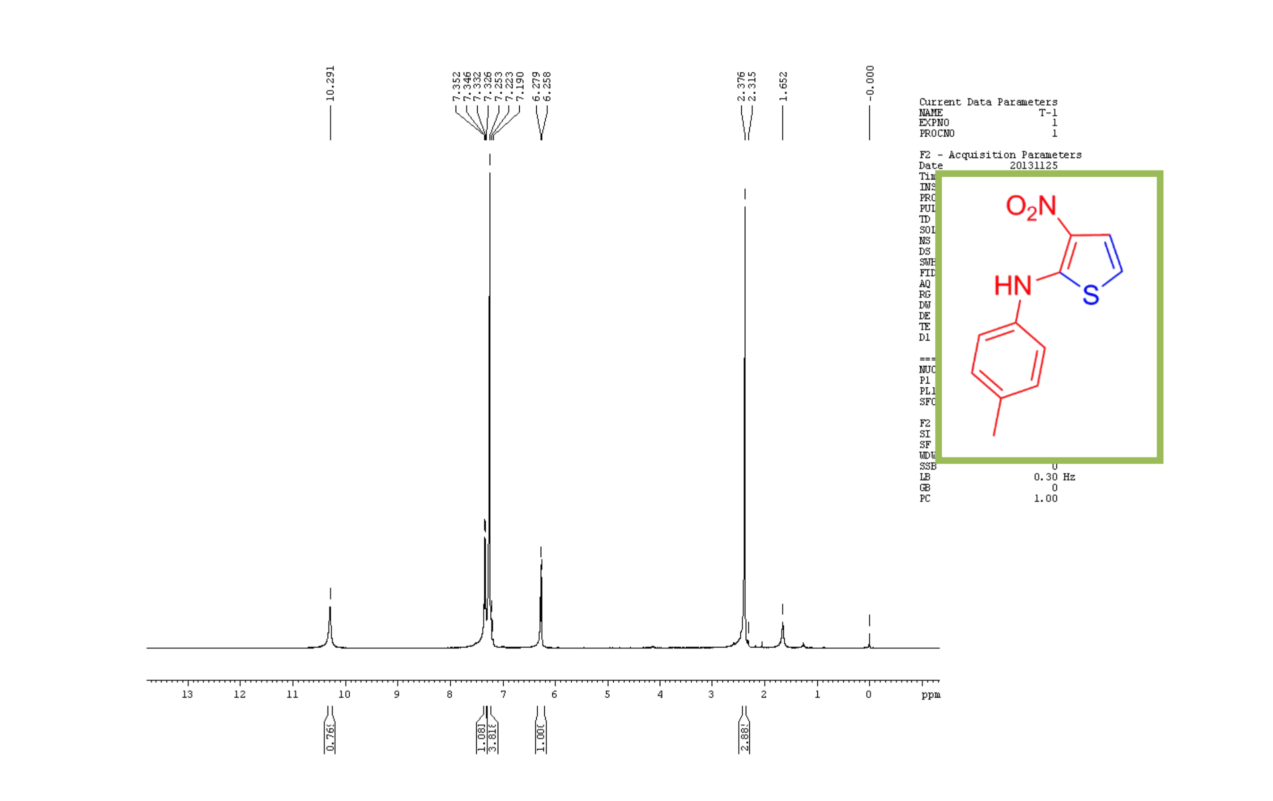

Figure 37 ^{1}H NMR Spectrum of **3f** ($CDCl_3$)

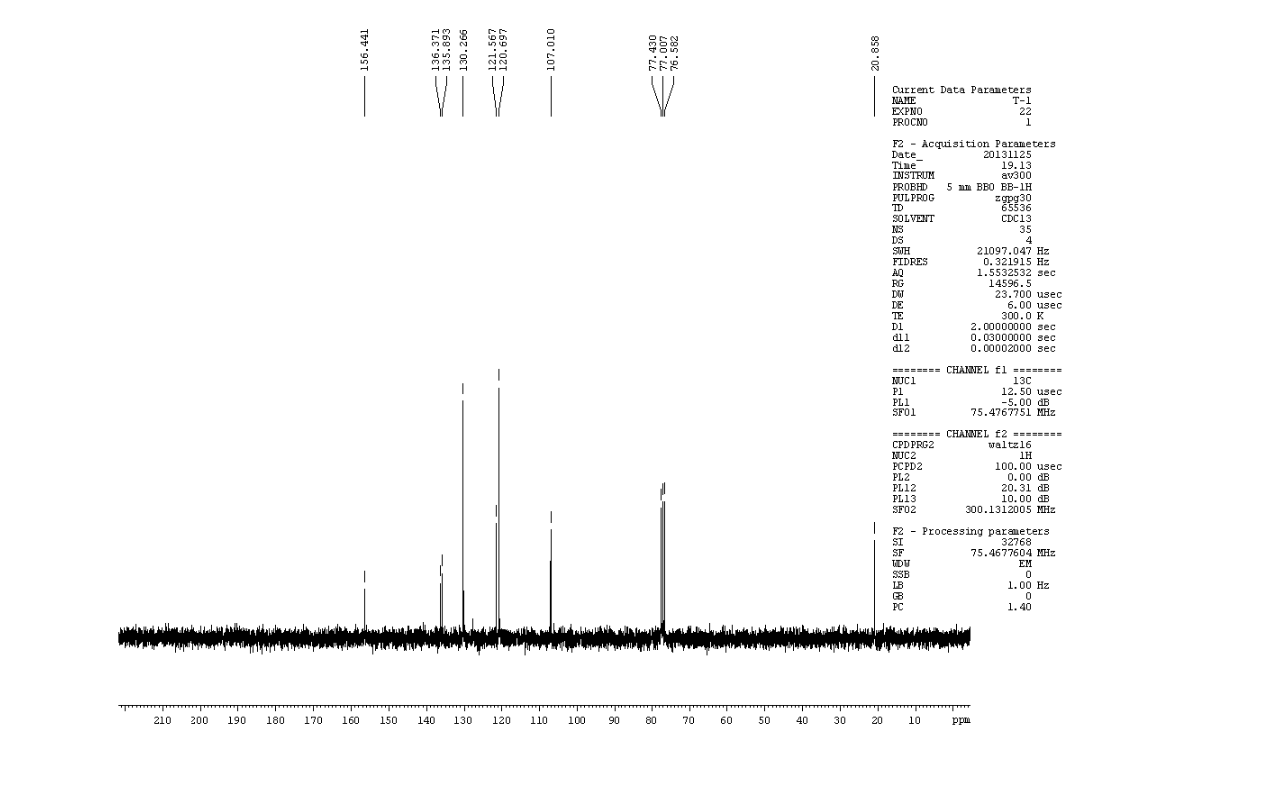

Figure 38 ^{13}C NMR Spectrum of **3f** ($CDCl_3$)

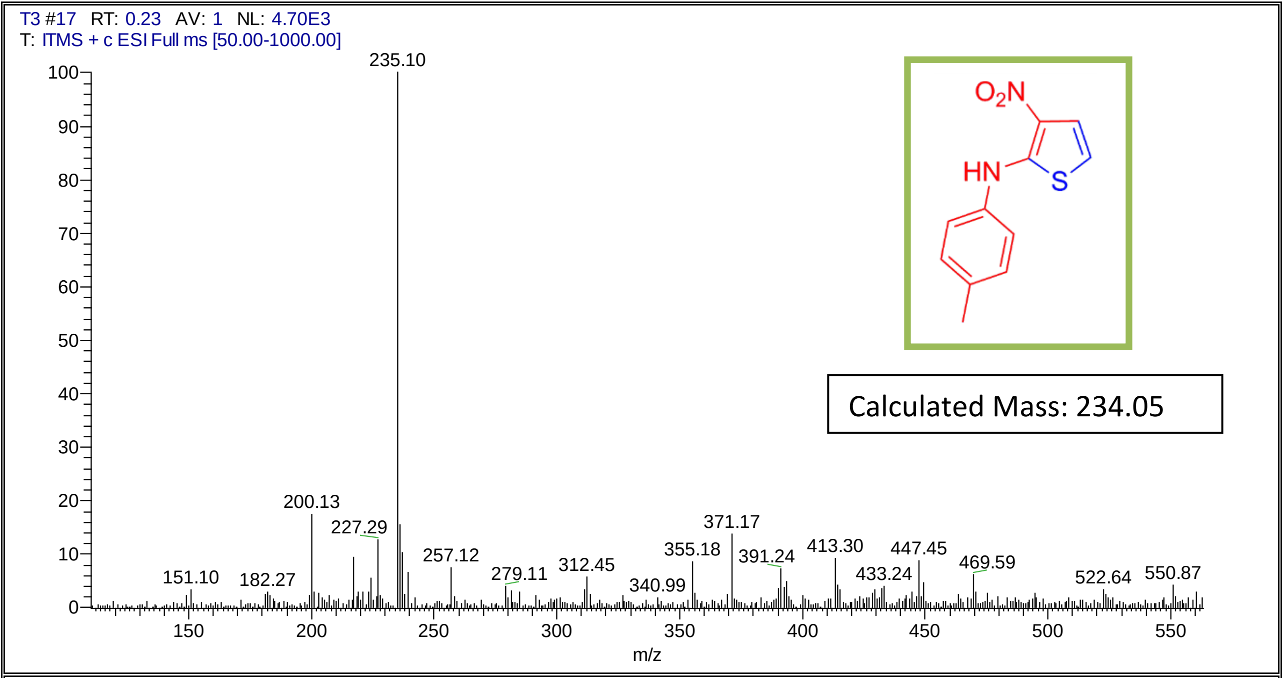

Figure 39 ESI mass spectrum of Spectrum of **3f**

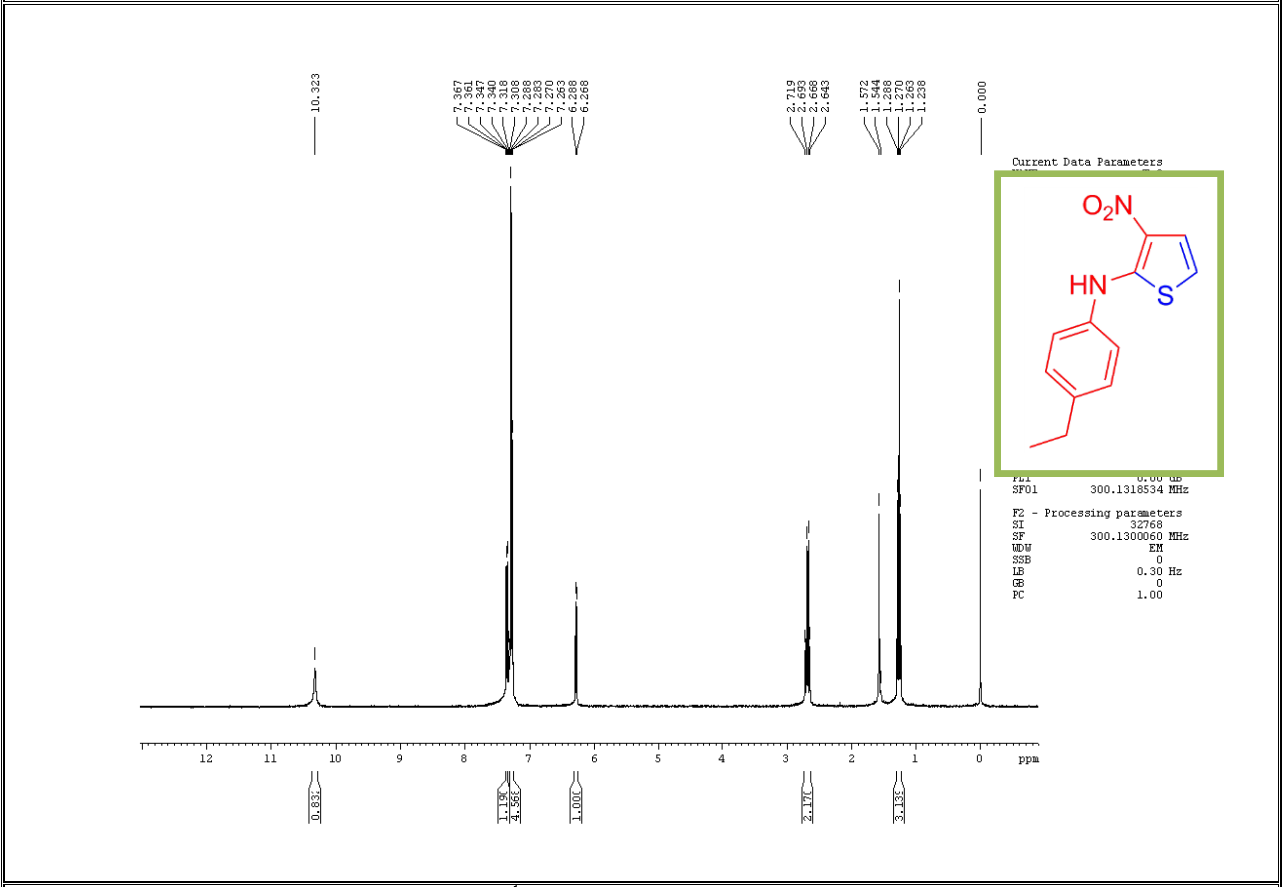

Figure 40 ^{1}H NMR Spectrum **3g** ($CDCl_3$)

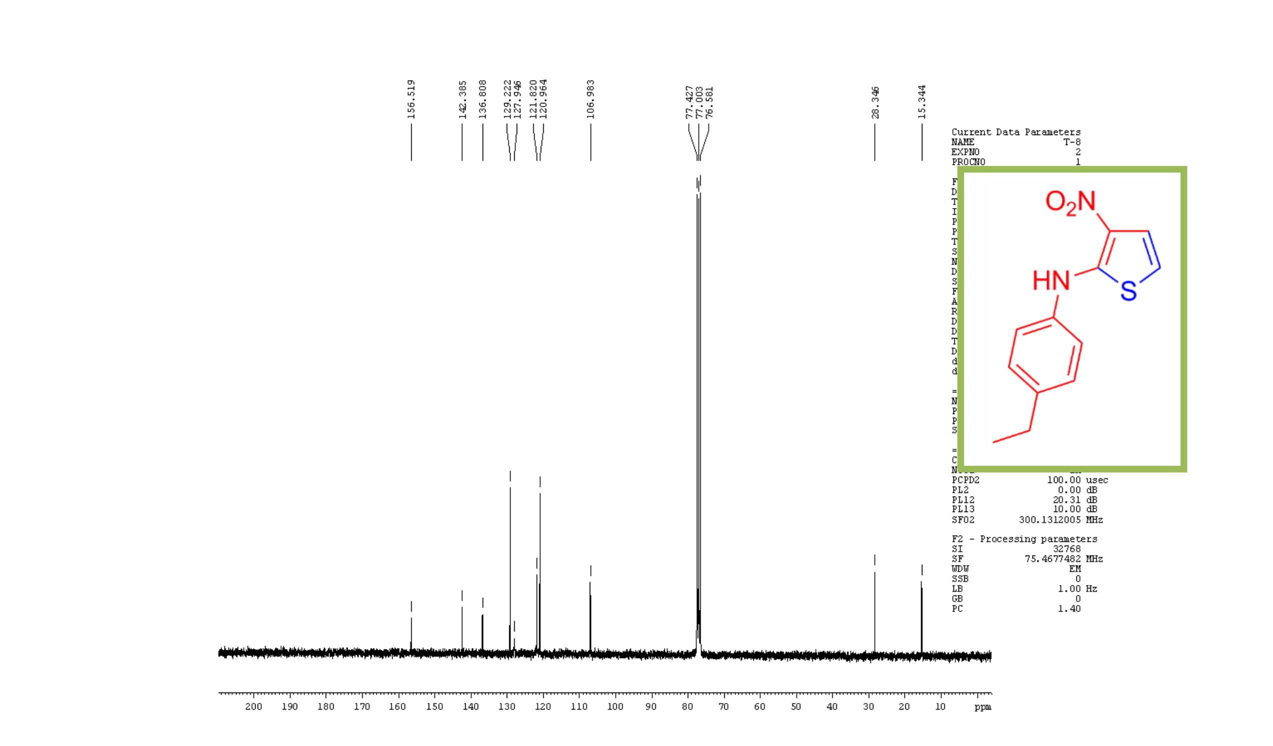

Figure 41 ^{13}C NMR Spectrum of **3g** ($CDCl_3$)

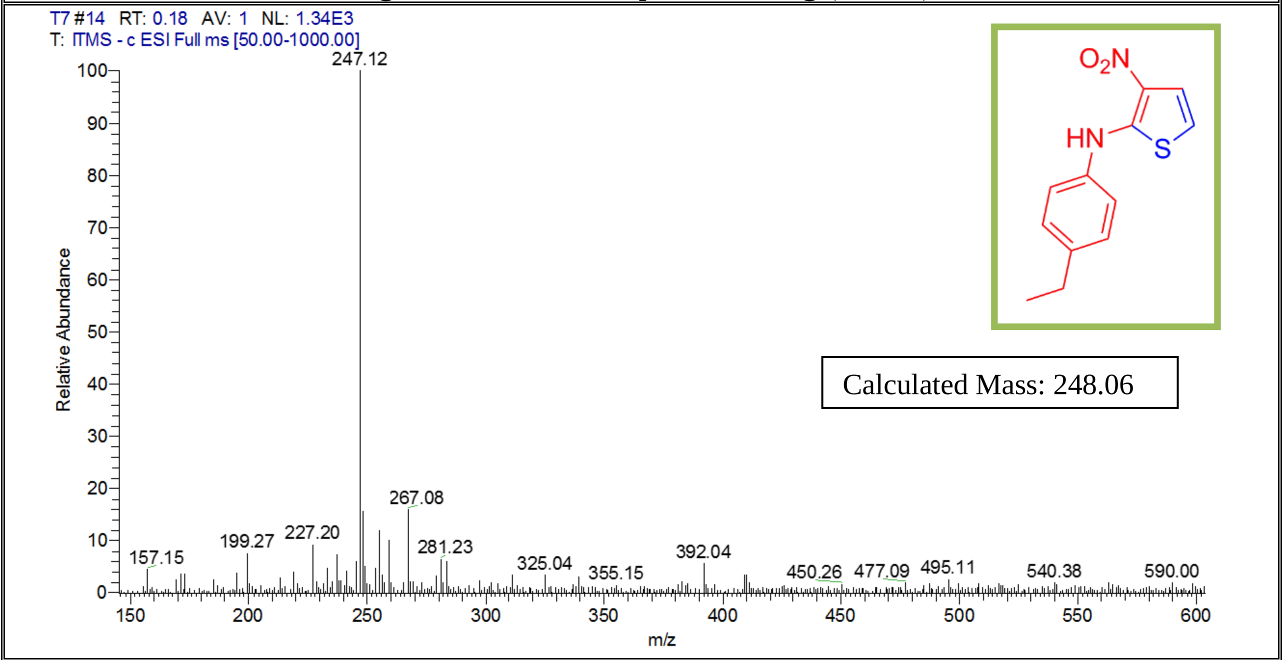

Figure 42 ESI mass spectrum of Spectrum of **3g**

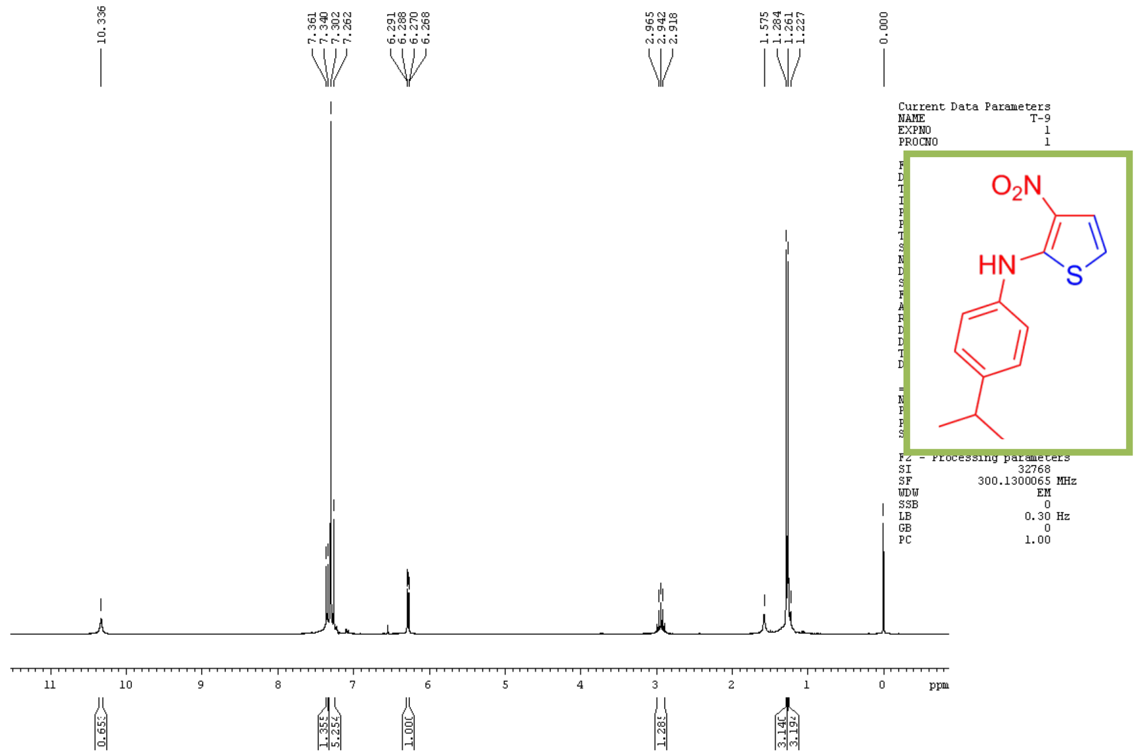

Figure 43 ^{1}H NMR Spectrum **3h** ($CDCl_3$)

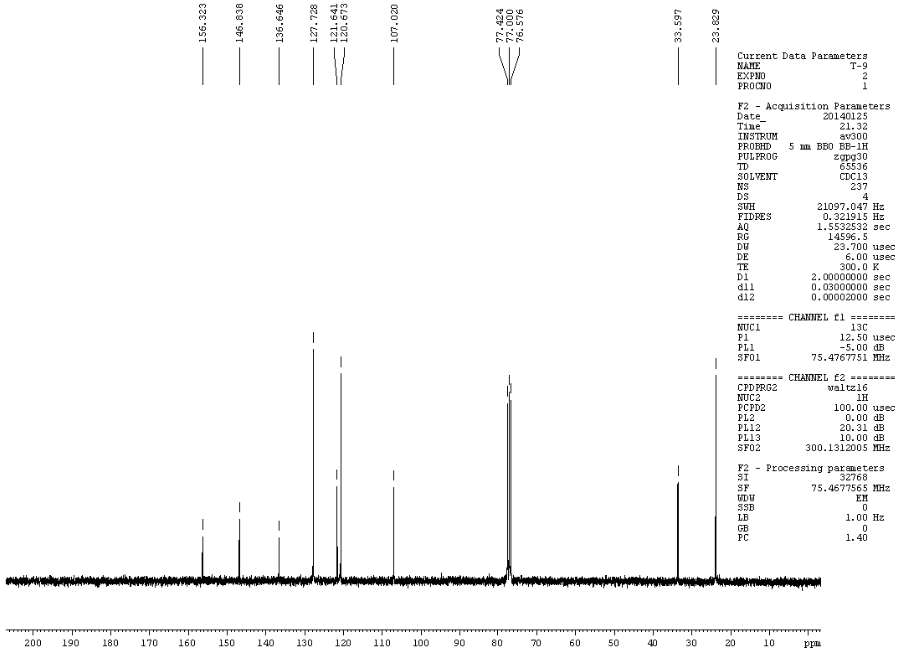

Figure 44 ^{13}C NMR Spectrum of **3h** ($CDCl_3$)

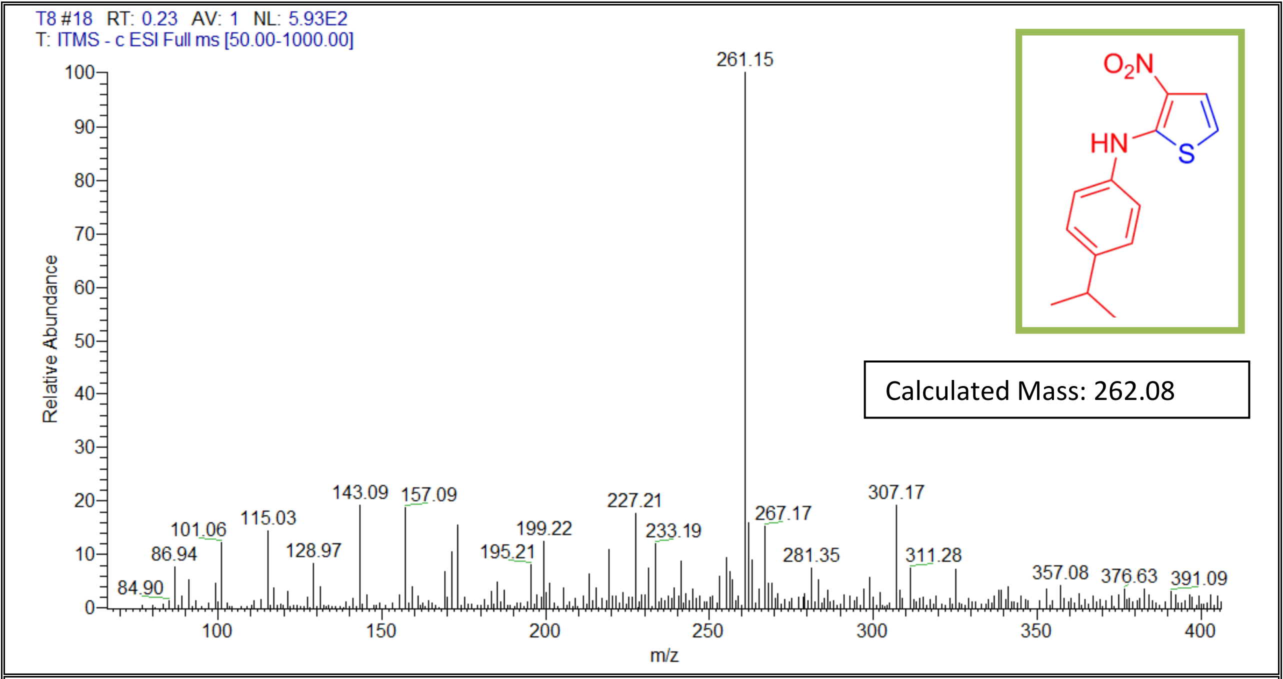

Figure 45 ESI mass spectrum of Spectrum of **3h**

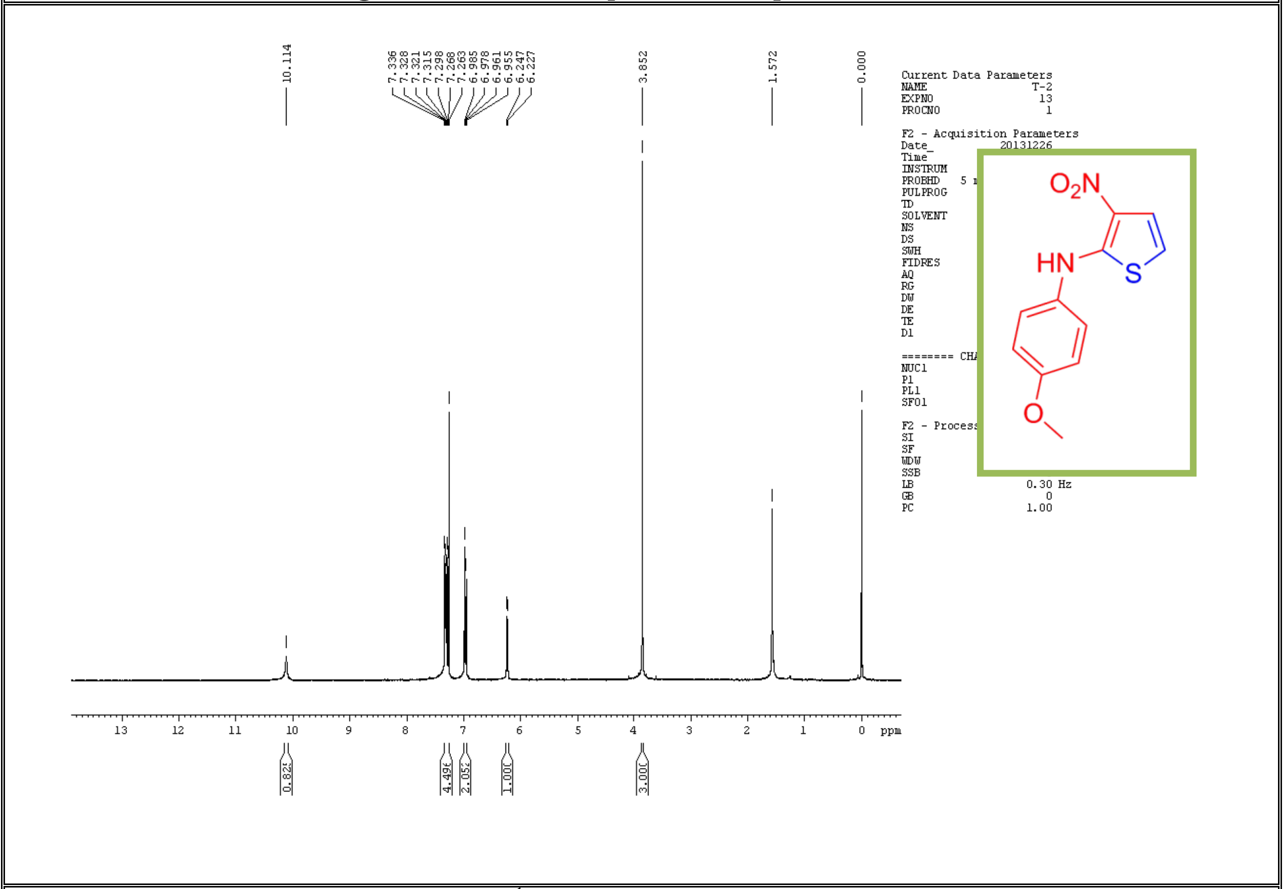

Figure 46 ^{1}H NMR Spectrum **3i** (CDCl$_3$)

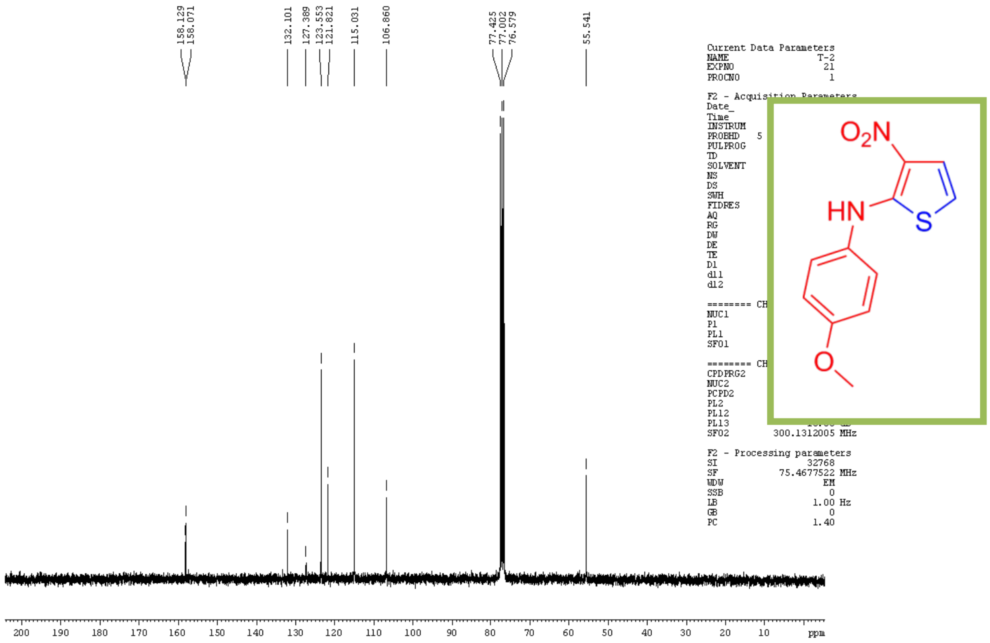

Figure 47 ^{13}C NMR Spectrum of **3i** ($CDCl_3$)

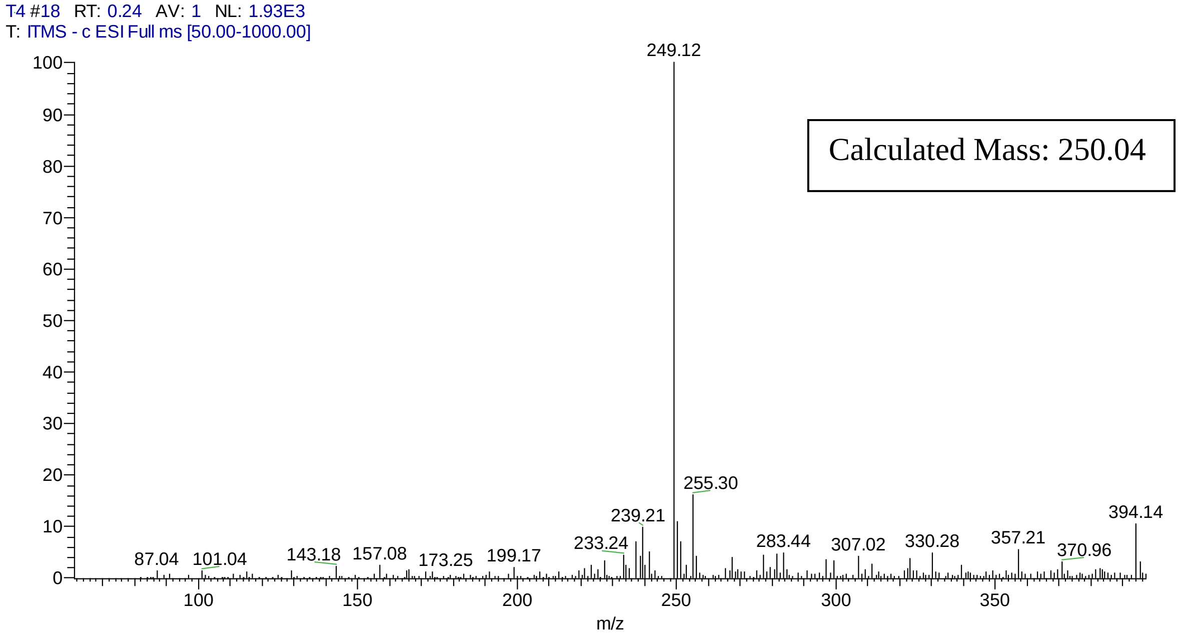

Figure 48 ESI mass spectrum of Spectrum of **3i**

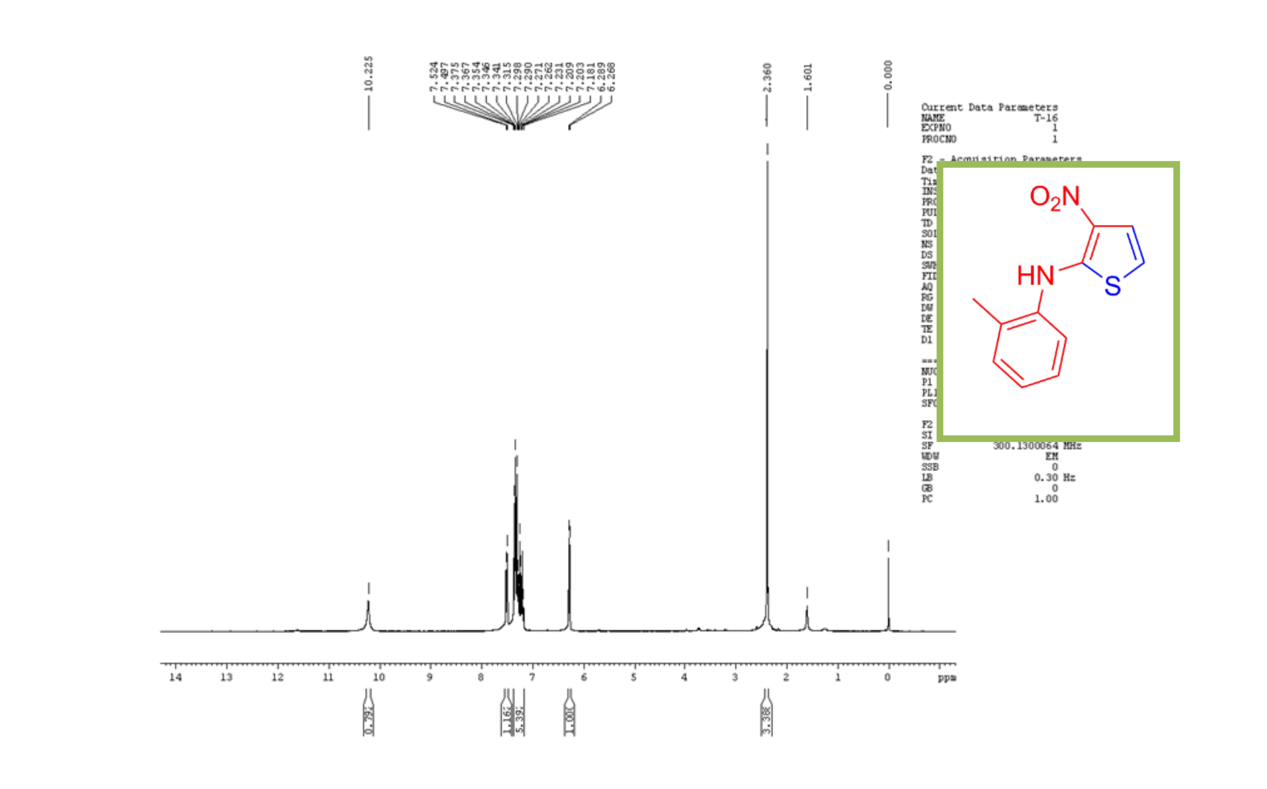

Figure 49 ^{1}H NMR Spectrum **3j** ($CDCl_3$)

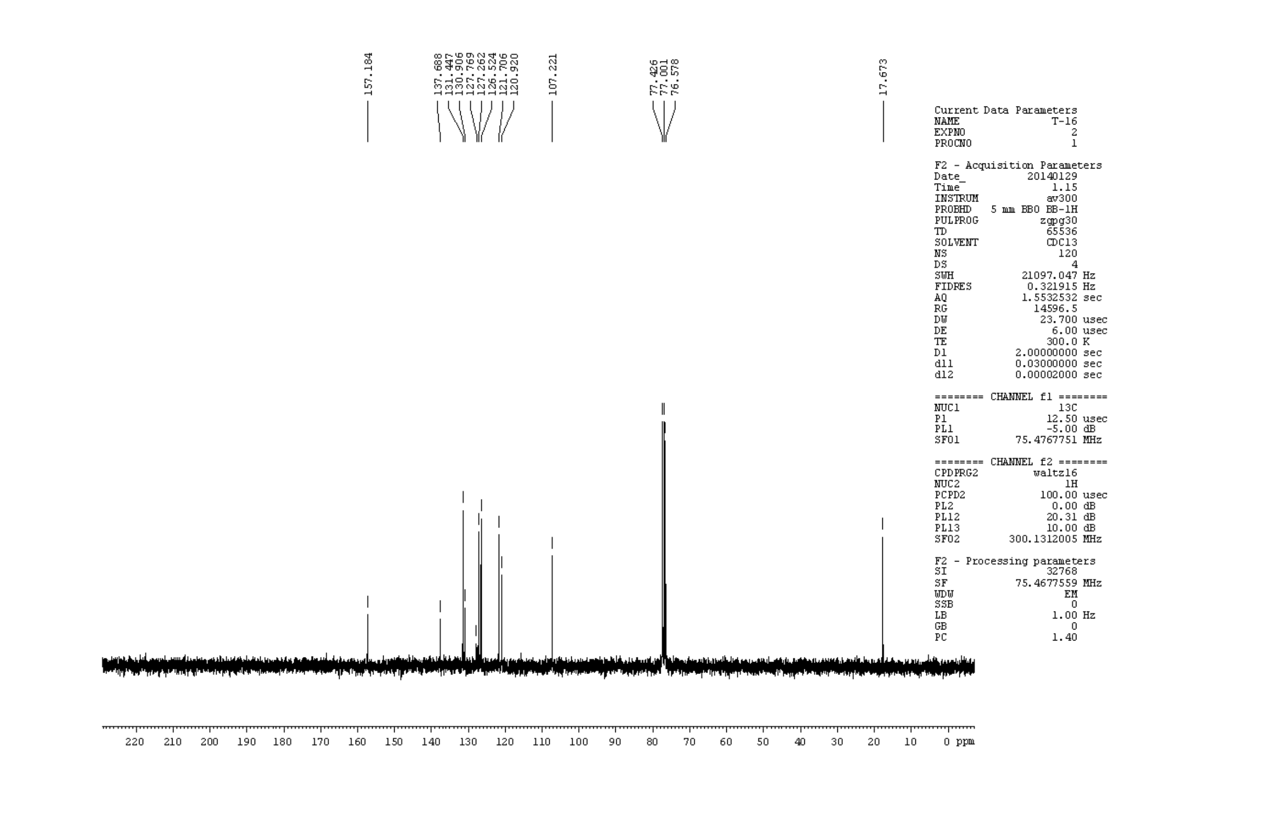

Figure 50 ^{13}C NMR Spectrum of **3j** ($CDCl_3$)

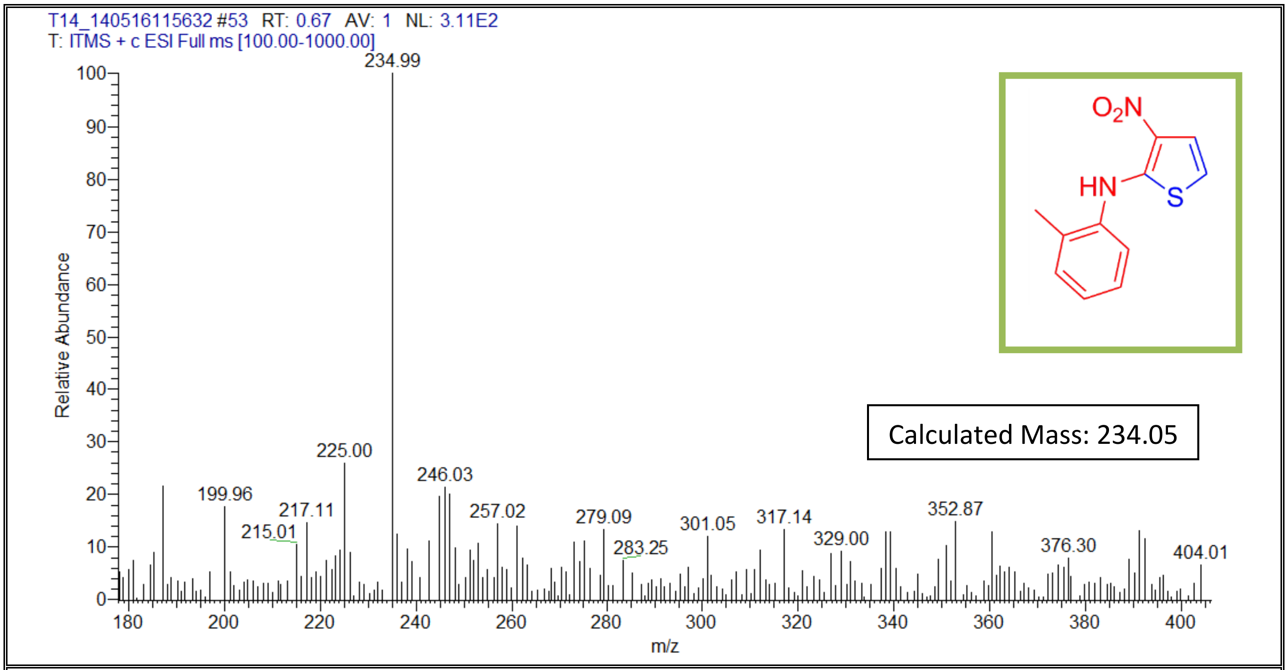

Figure 51 ESI mass spectrum of Spectrum of **3j**

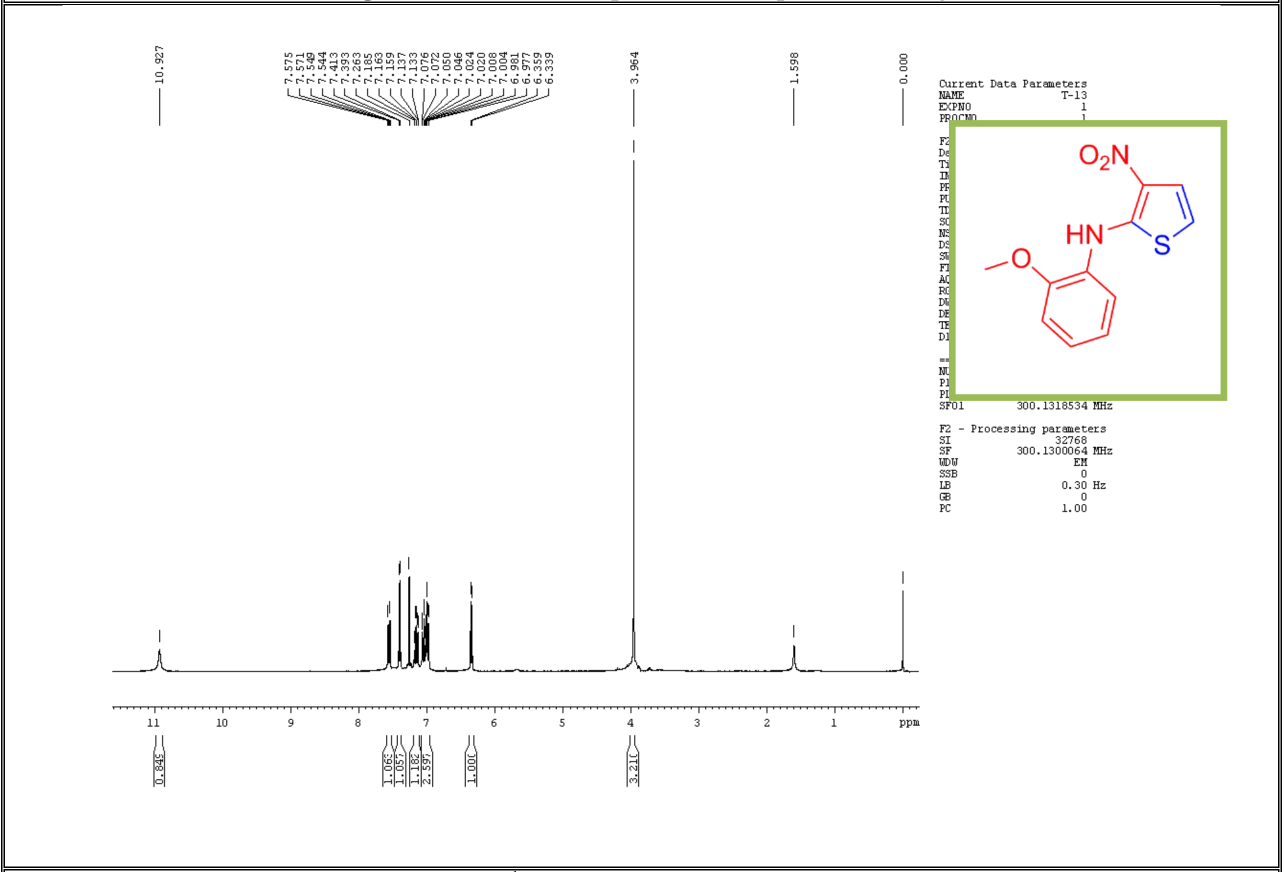

Figure 52 ^{1}H NMR Spectrum **3k** ($CDCl_3$)

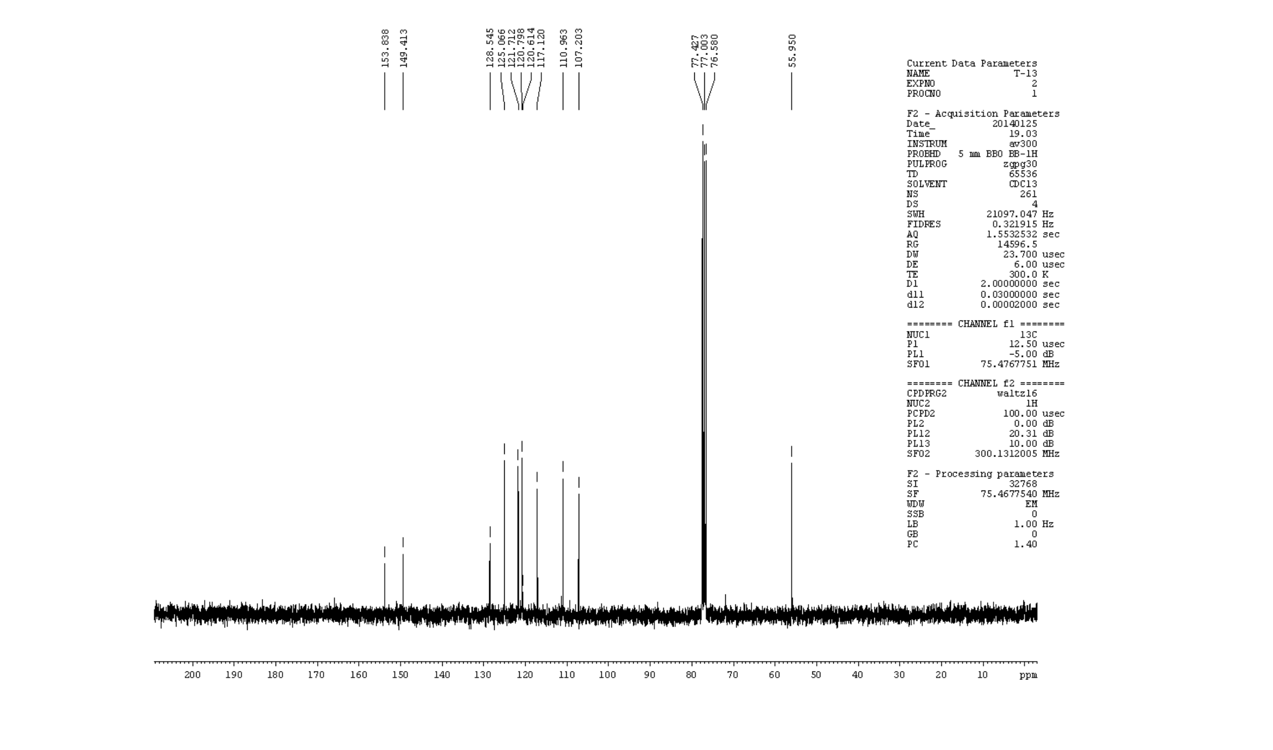

Figure 53 ^{13}C NMR Spectrum of **3k** ($CDCl_3$)

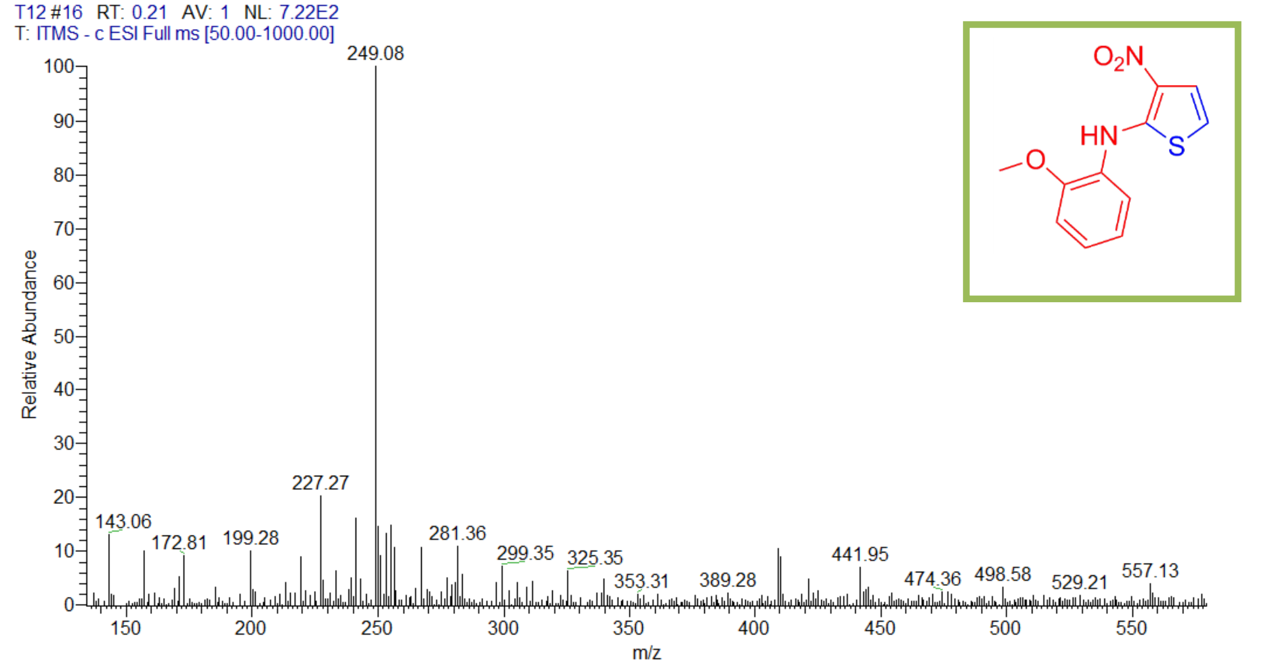

Figure 54 ESI mass spectrum of Spectrum of **3k**

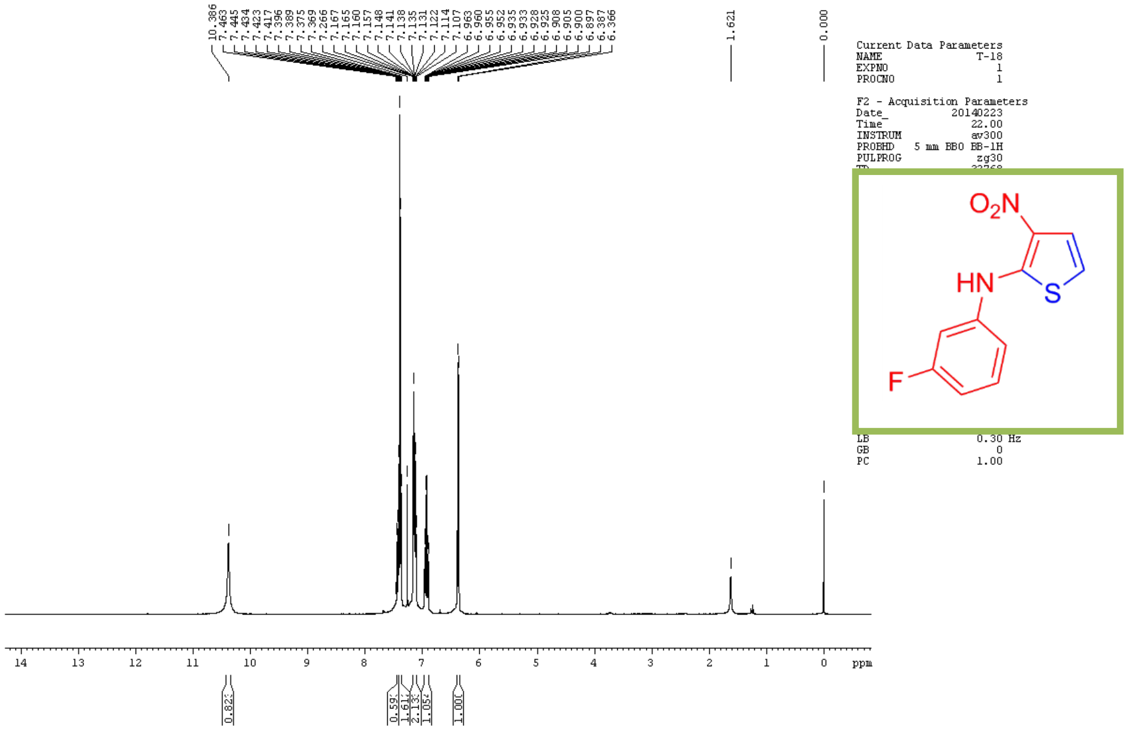

Figure 55 ^{1}H NMR Spectrum **3l** ($CDCl_3$)

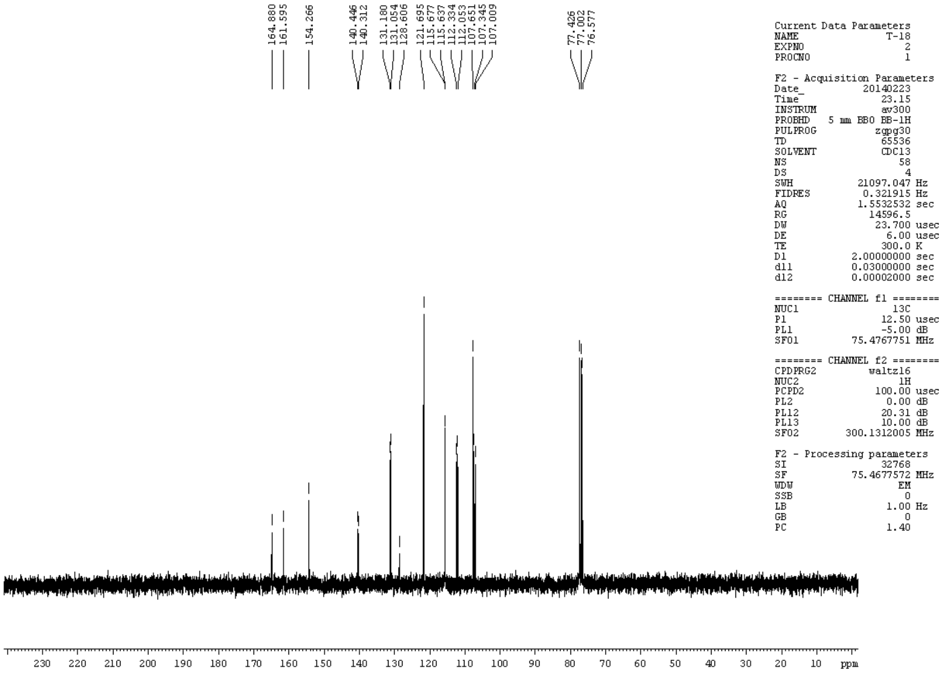

Figure 56 ^{13}C NMR Spectrum of **3l** ($CDCl_3$)

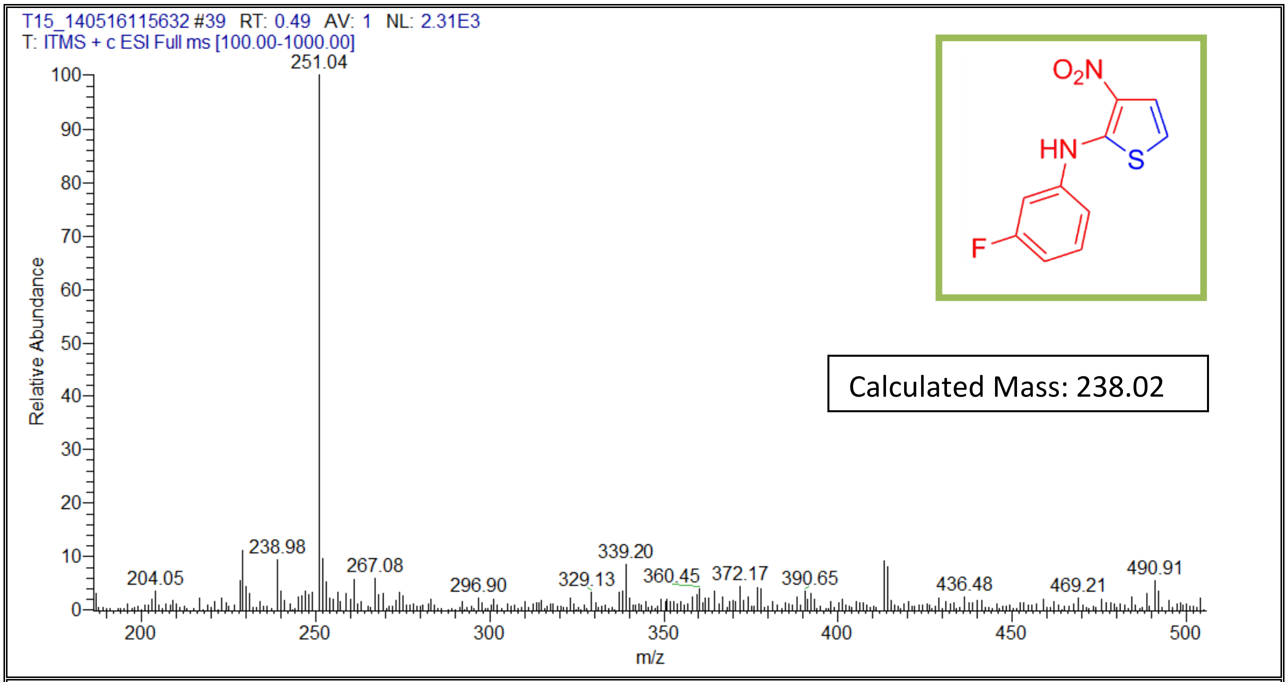

Figure 57 ESI mass spectrum of Spectrum of **3l**

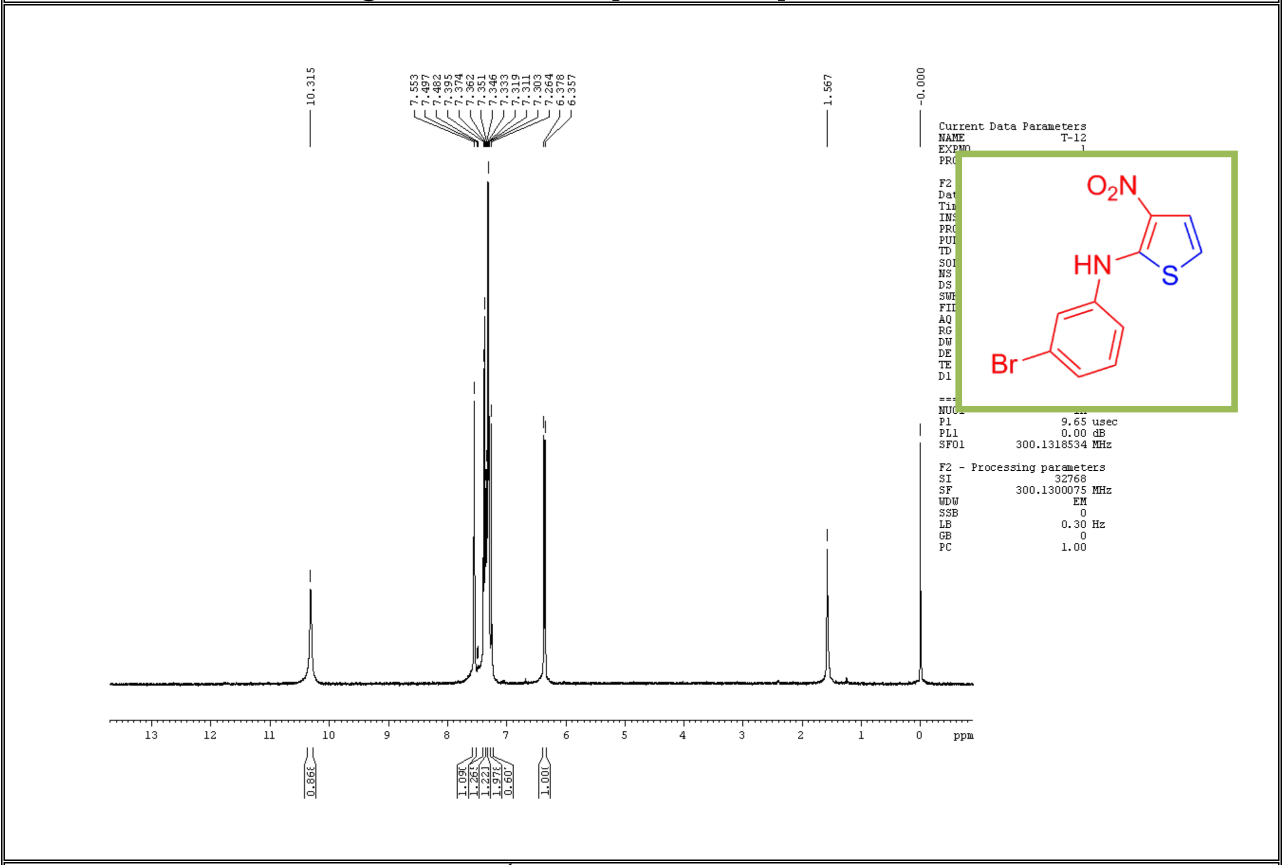

Figure 58 ^{1}H NMR Spectrum **3m** ($CDCl_3$)

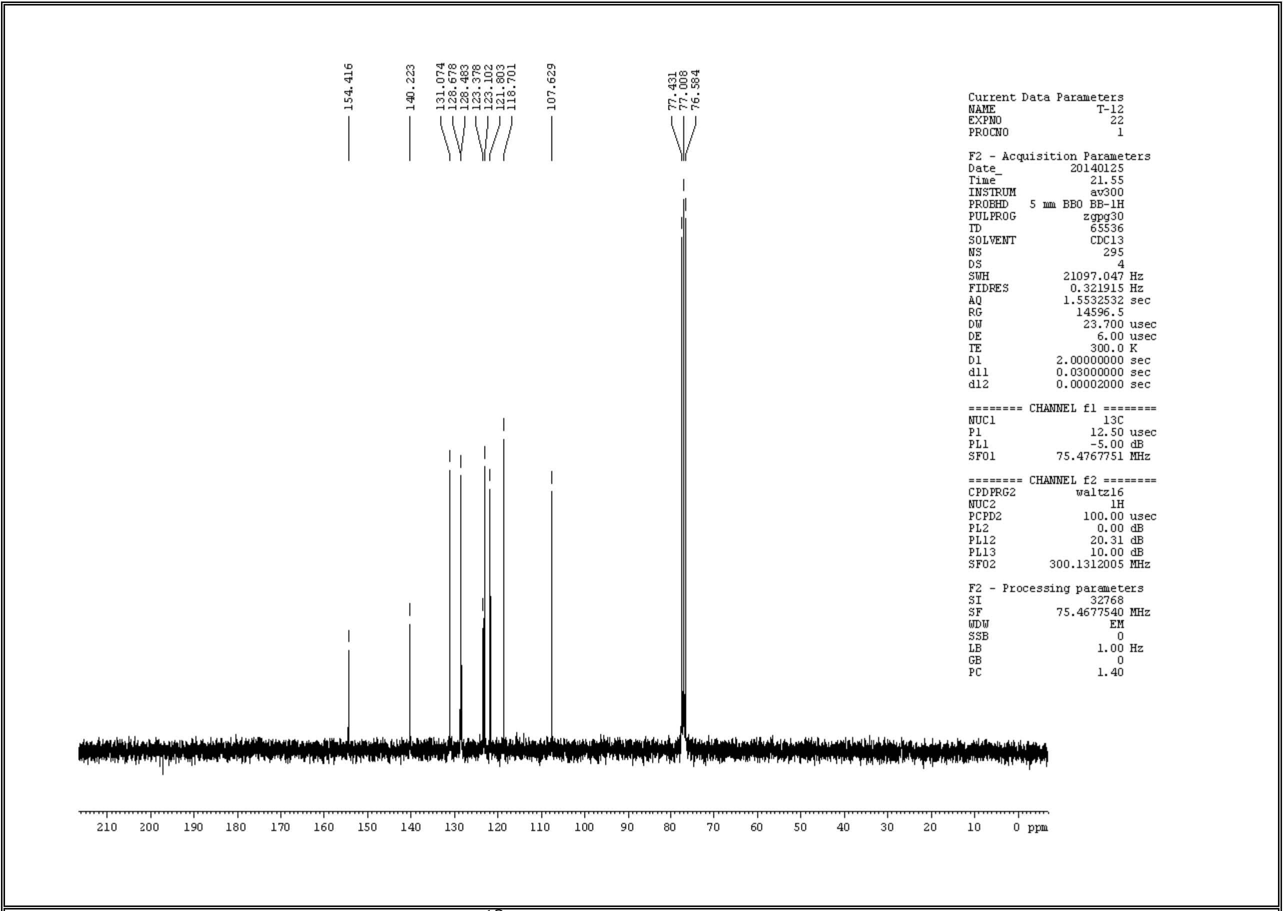

Figure 59 ^{13}C NMR Spectrum of **3m** ($CDCl_3$)

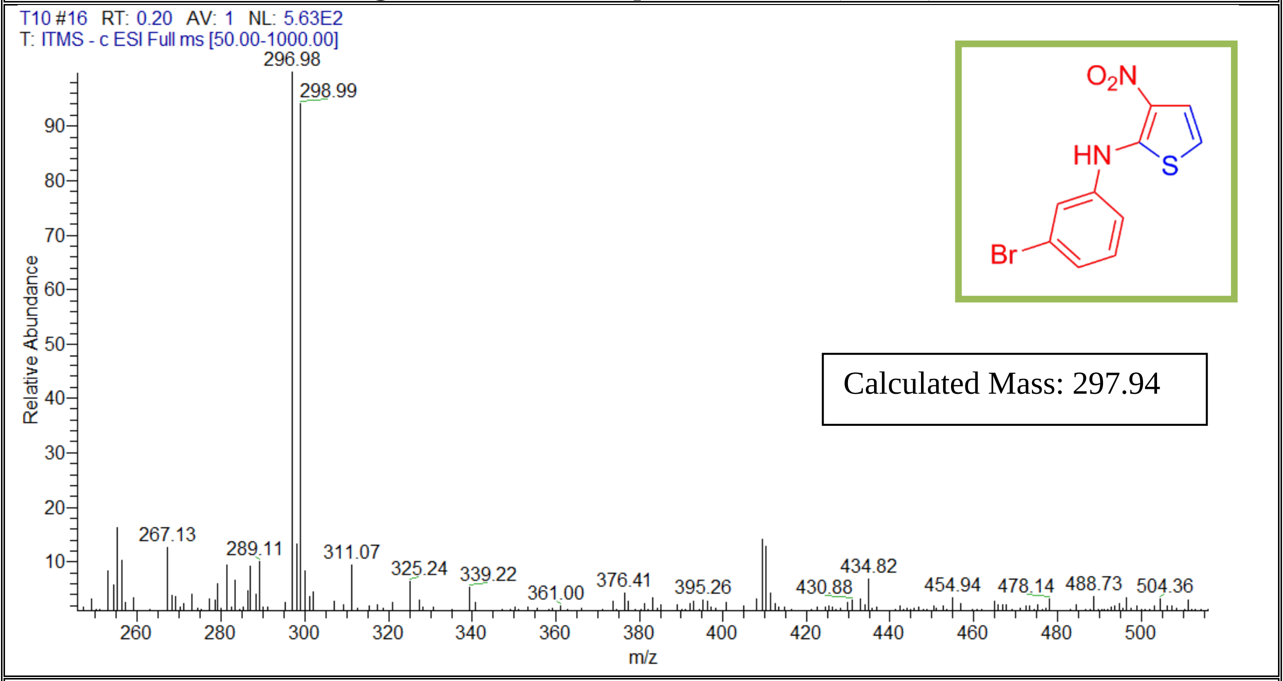

Figure 60 ESI mass spectrum of Spectrum of **3m**

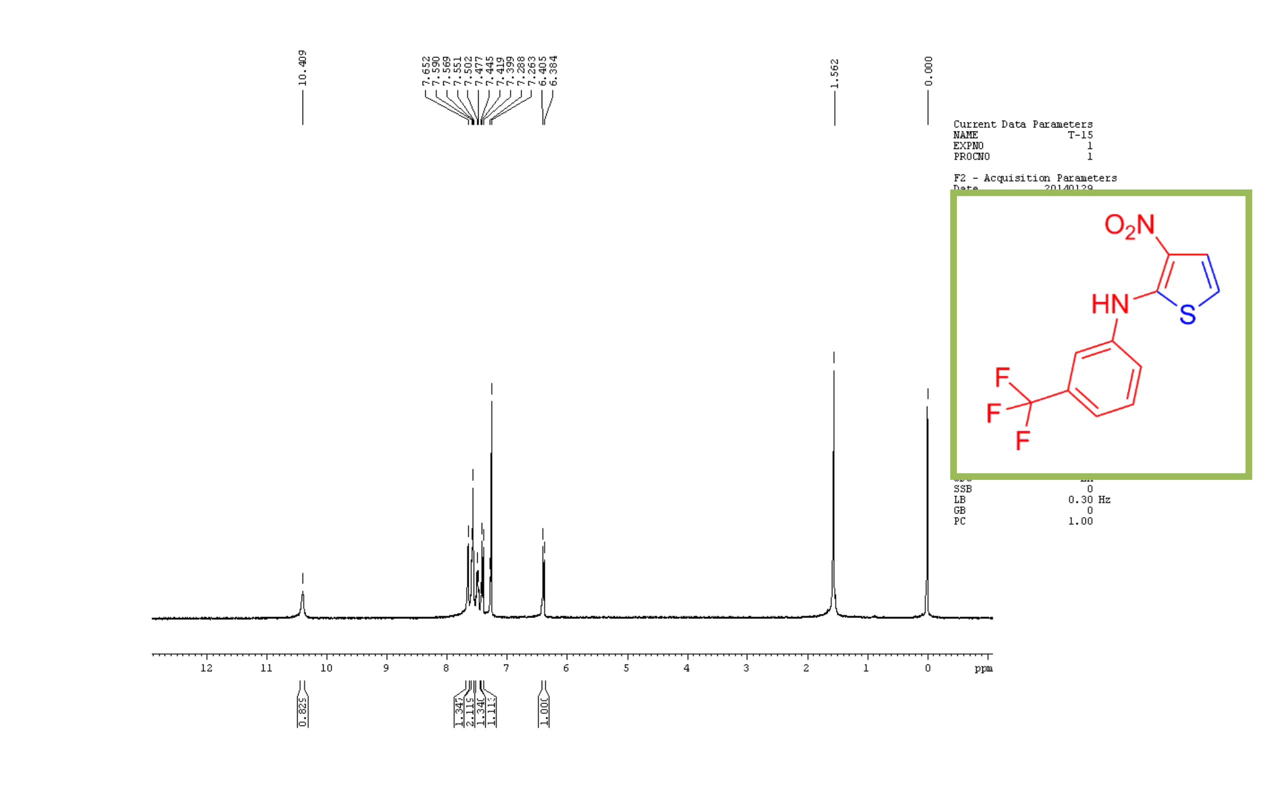

Figure 61 ^{1}H NMR Spectrum **3n** ($CDCl_3$)

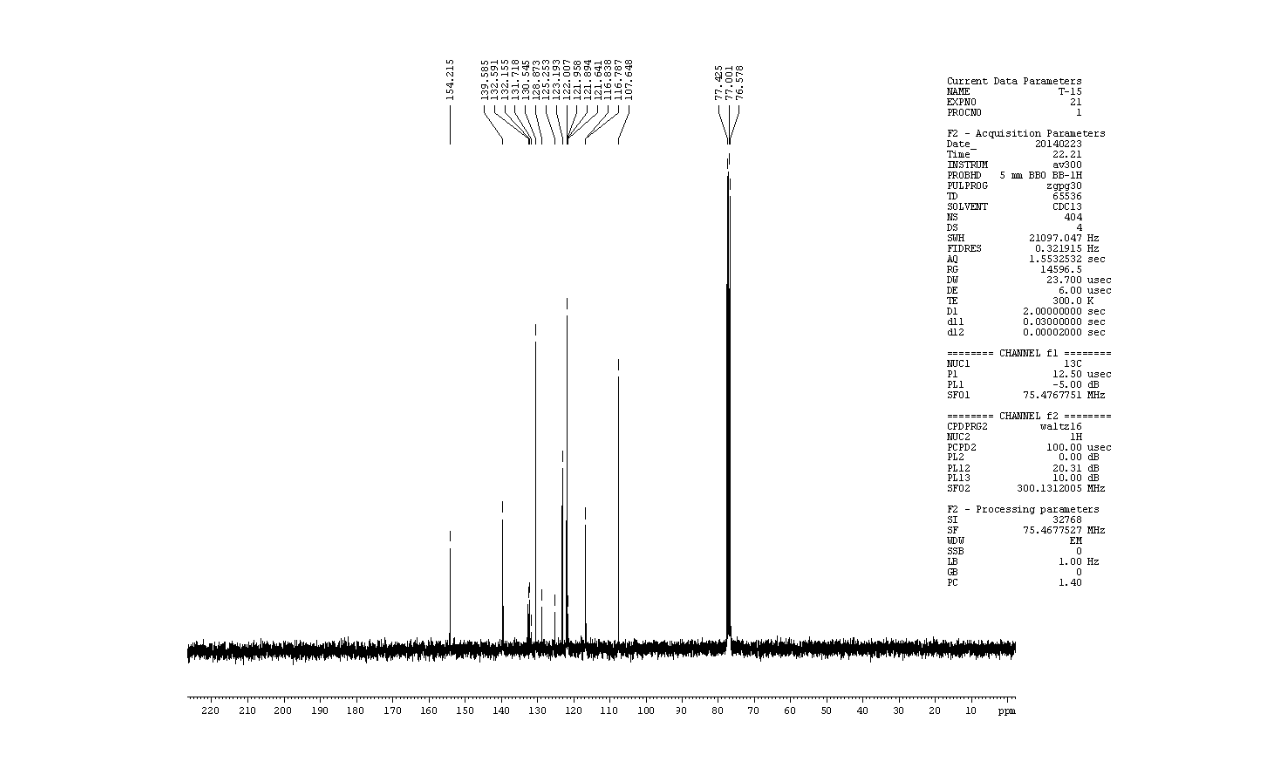

Figure 62 ^{13}C NMR Spectrum of **3n** ($CDCl_3$)

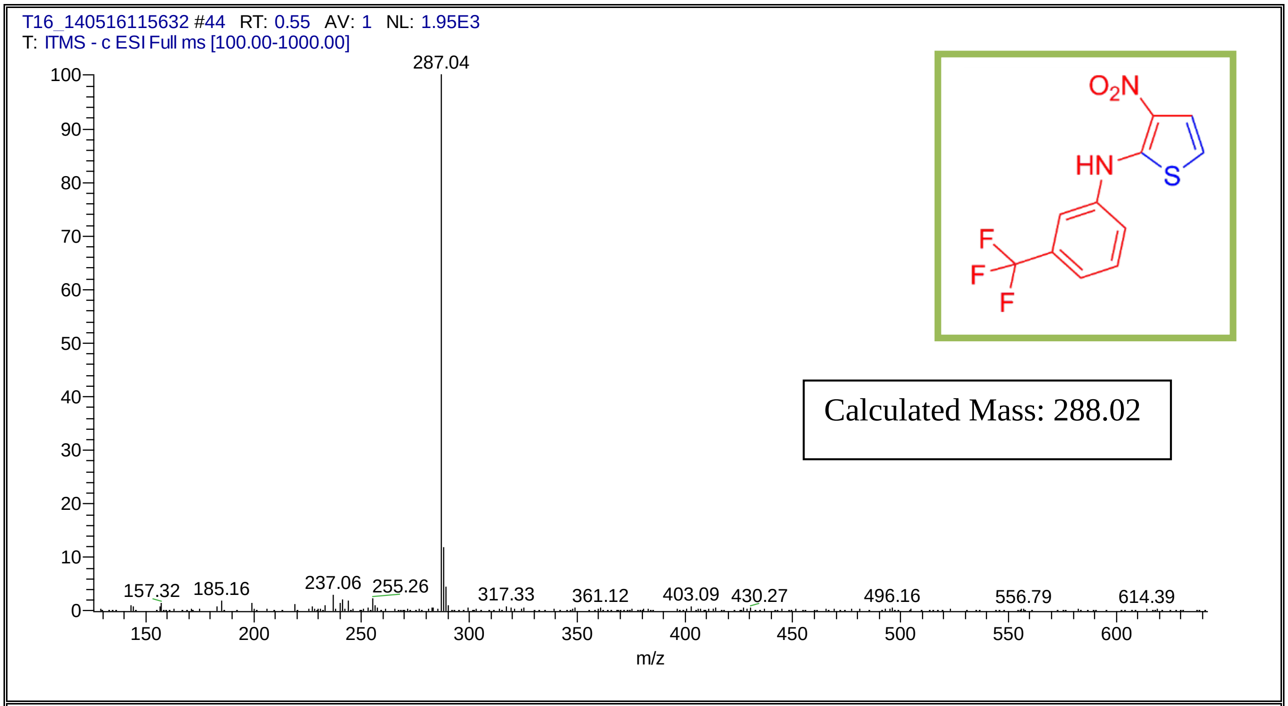

Figure 63 ESI mass spectrum of Spectrum of **3n**

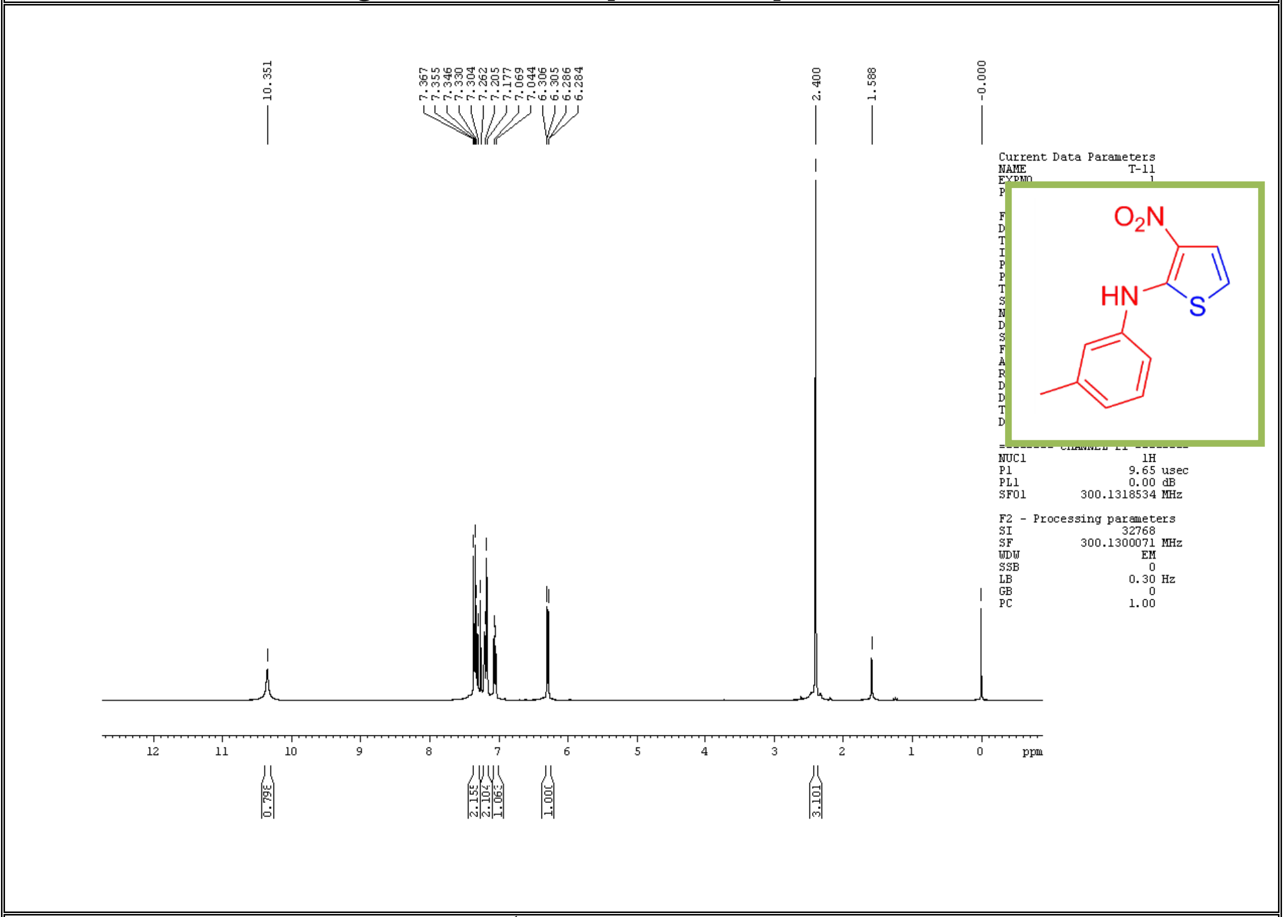

Figure 64 ^{1}H NMR Spectrum **3o** ($CDCl_3$)

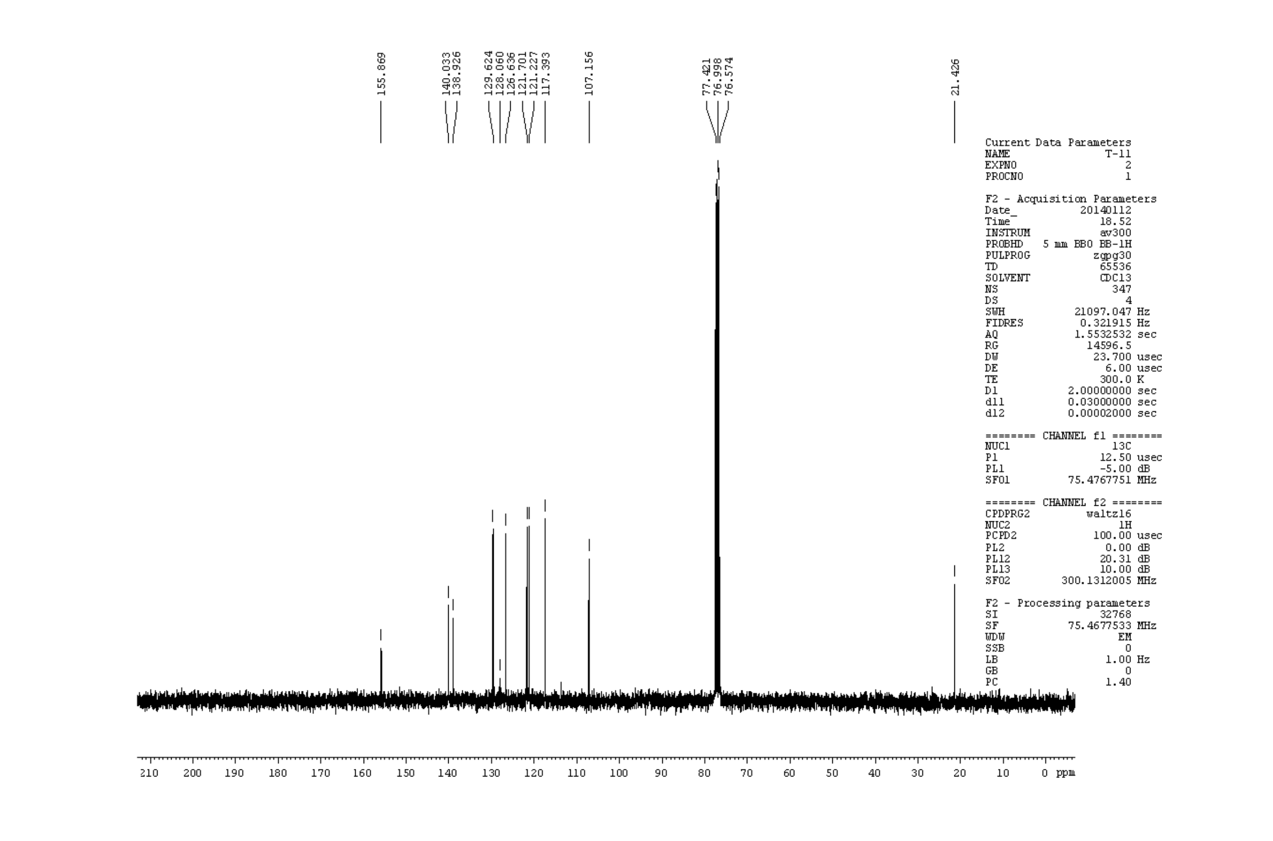

Figure 65 ^{13}C NMR Spectrum of **3o** ($CDCl_3$)

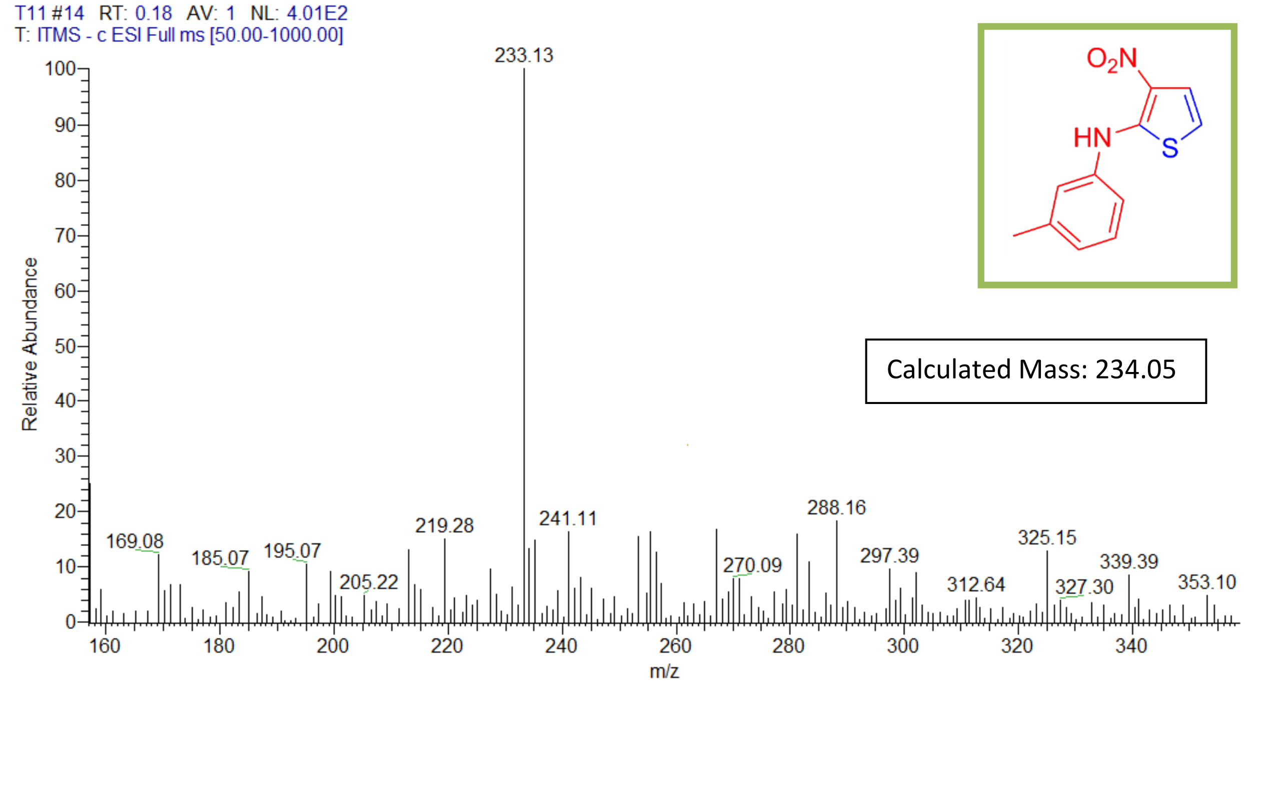

Figure 66 ESI mass spectrum of Spectrum of **3o**

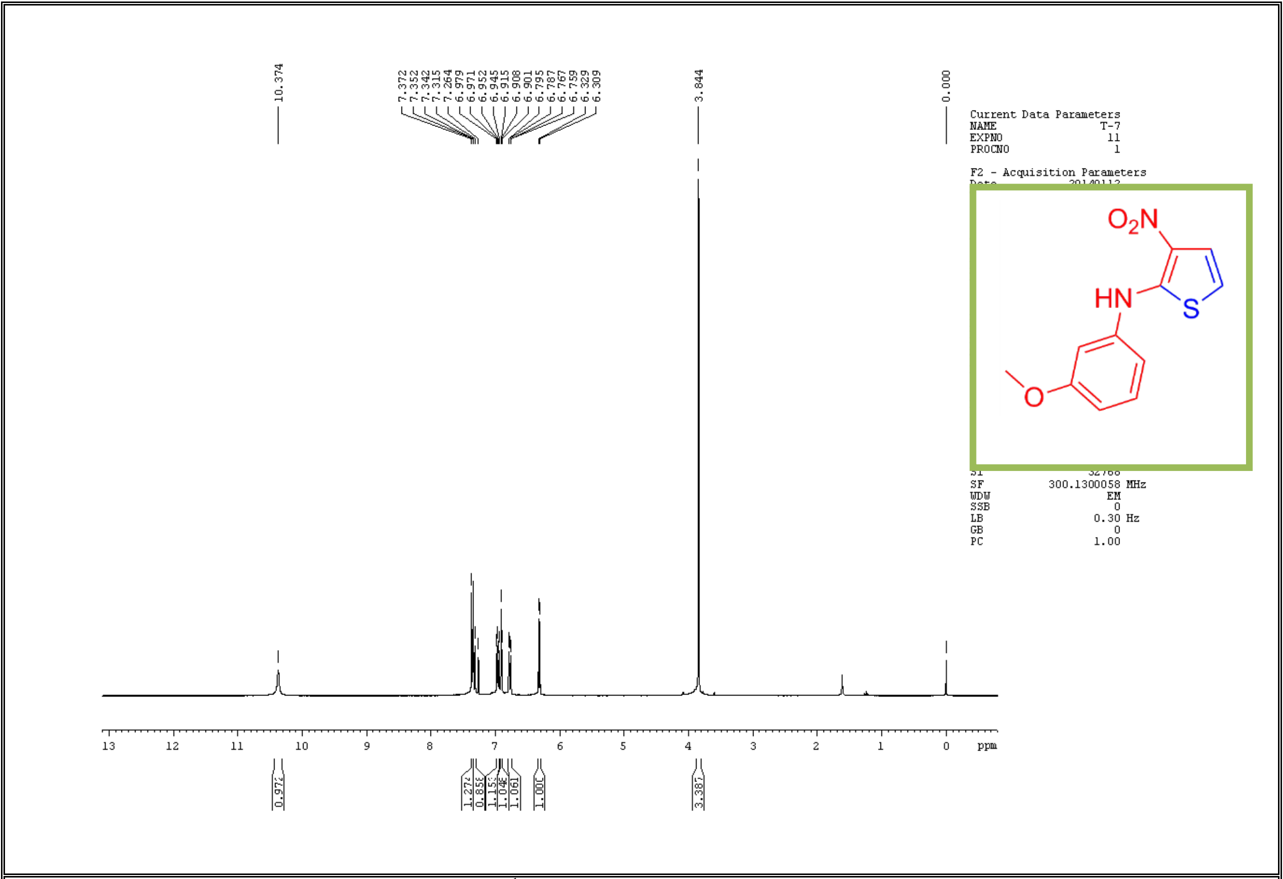

Figure 67 ^{1}H NMR Spectrum **3p** ($CDCl_3$)

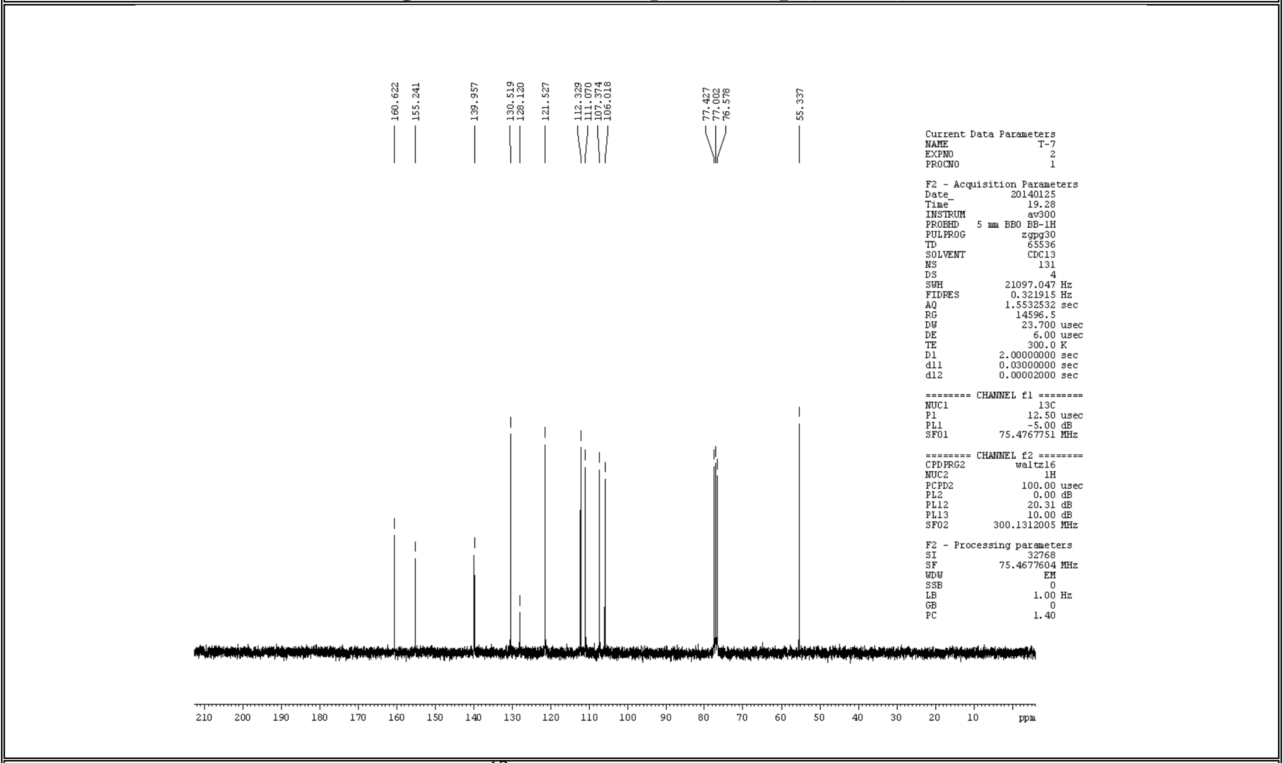

Figure 68 ^{13}C NMR Spectrum of **3p** ($CDCl_3$)

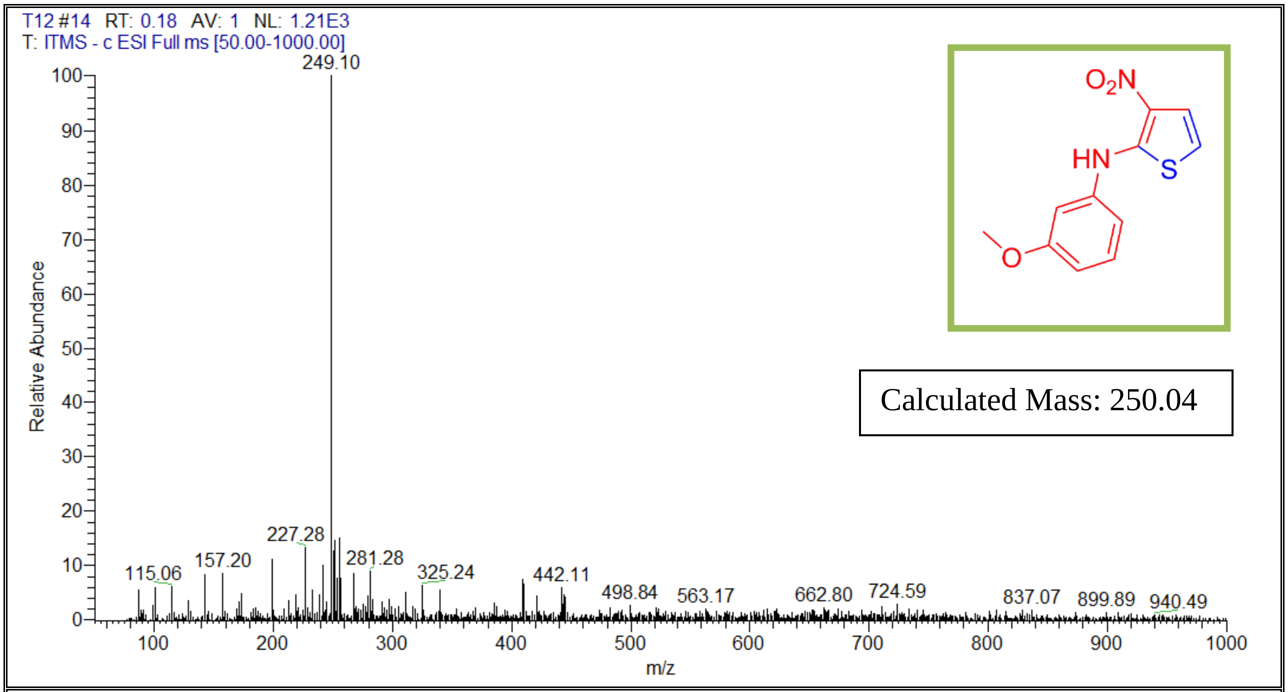

Figure 69 ESI mass spectrum of Spectrum of **3p**

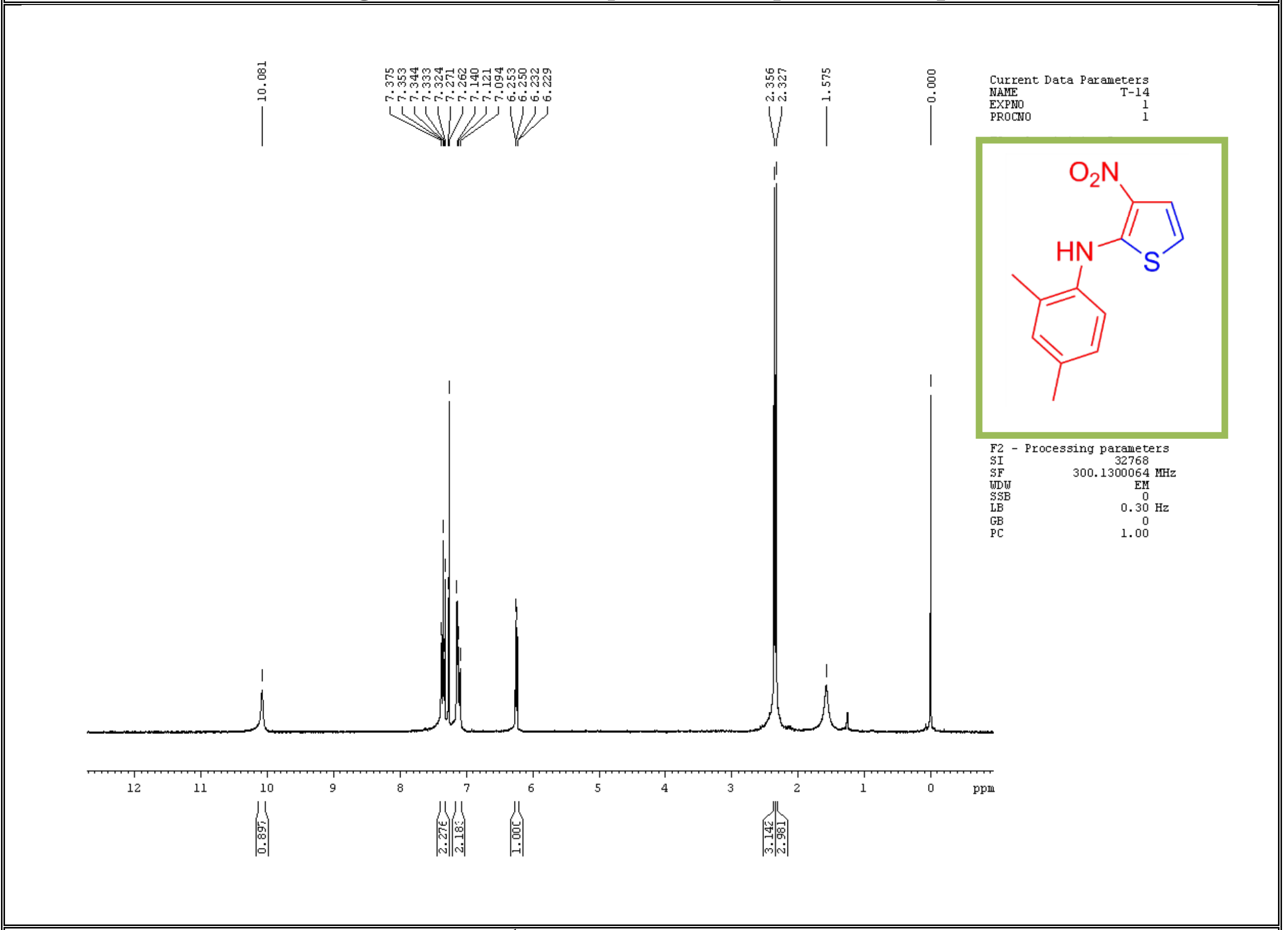

Figure 70 ^{1}H NMR Spectrum **3q** ($CDCl_3$)

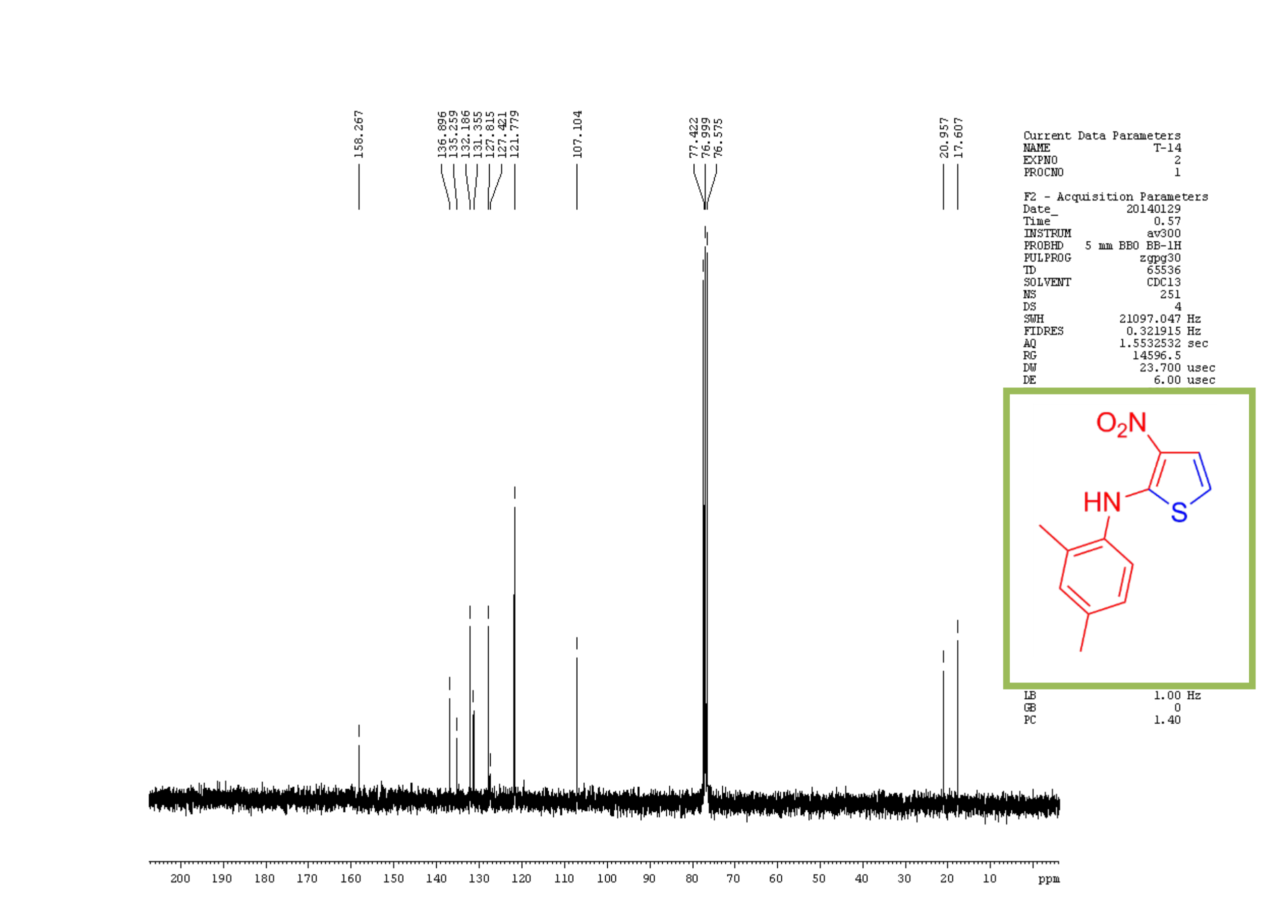

Figure 71 ^{13}C NMR Spectrum of **3q** ($CDCl_3$)

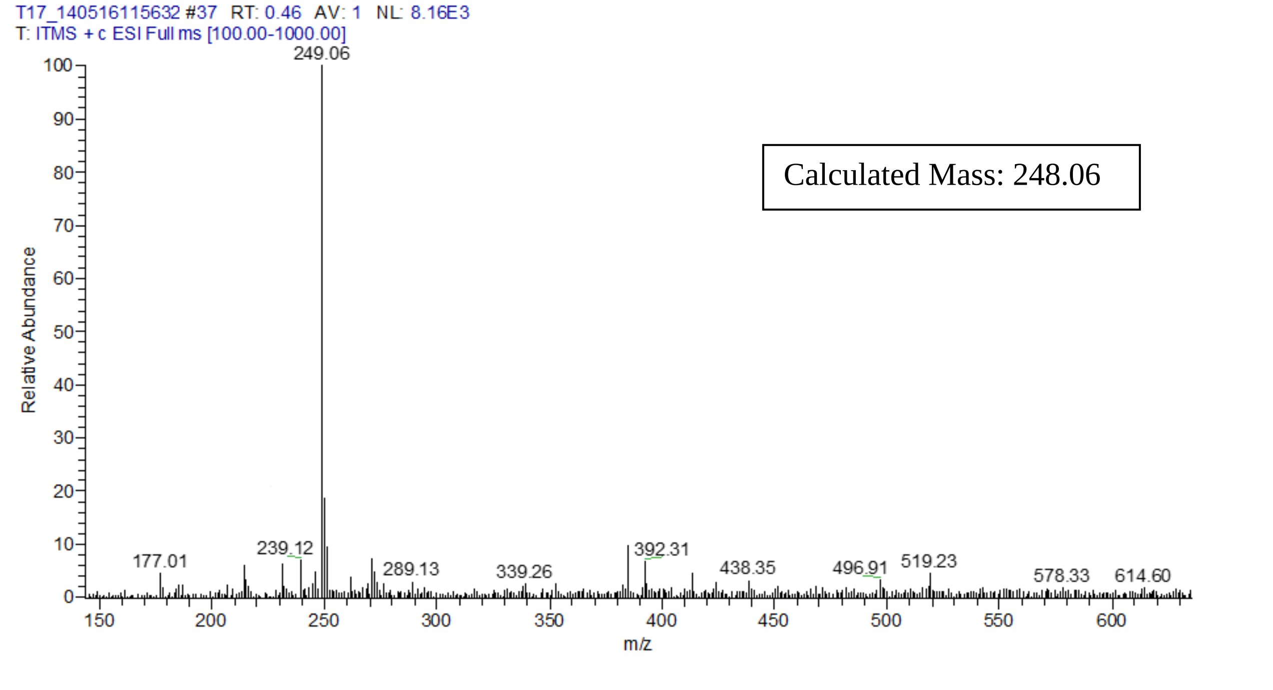

Figure 72 ESI mass spectrum of Spectrum of **3q**

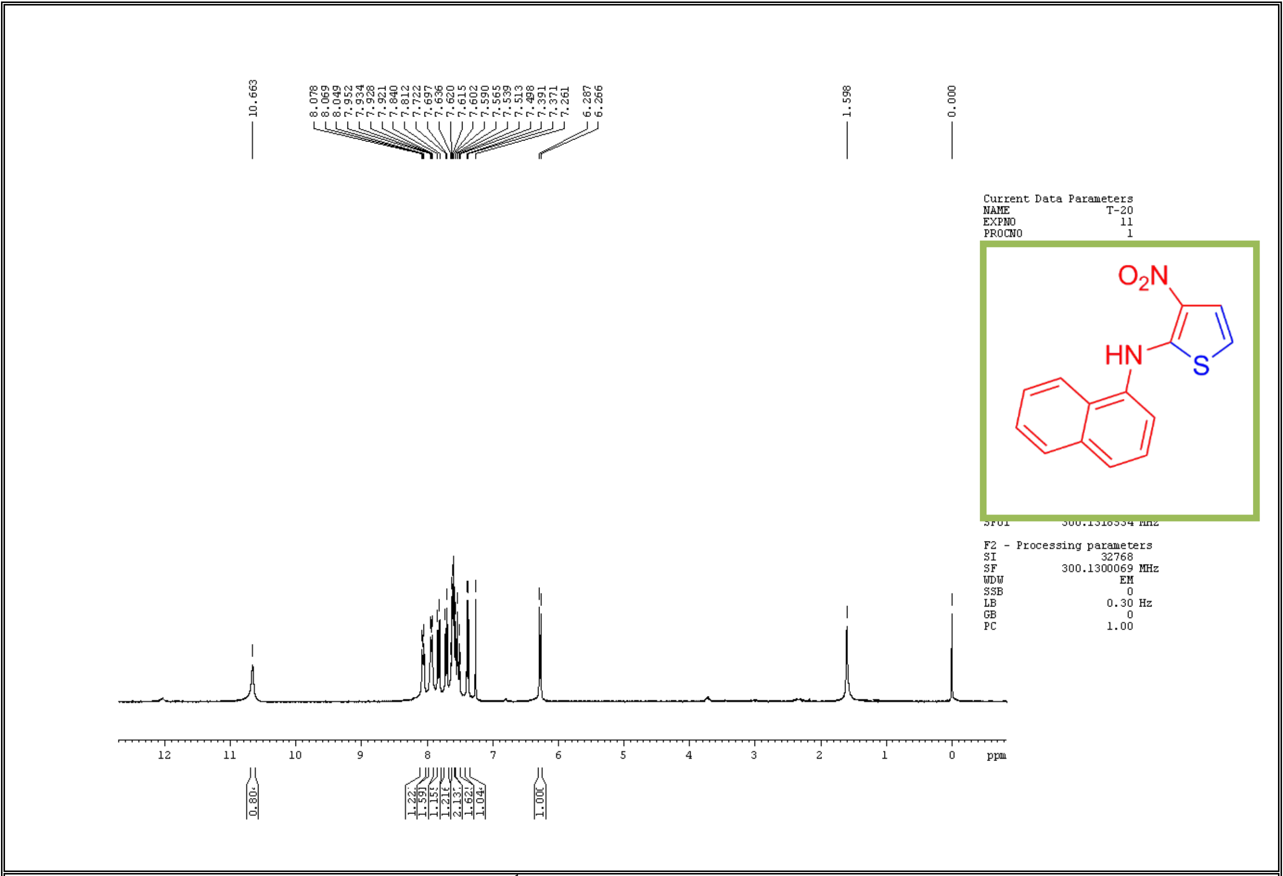

Figure 73 ^{1}H NMR Spectrum **3r** ($CDCl_3$)

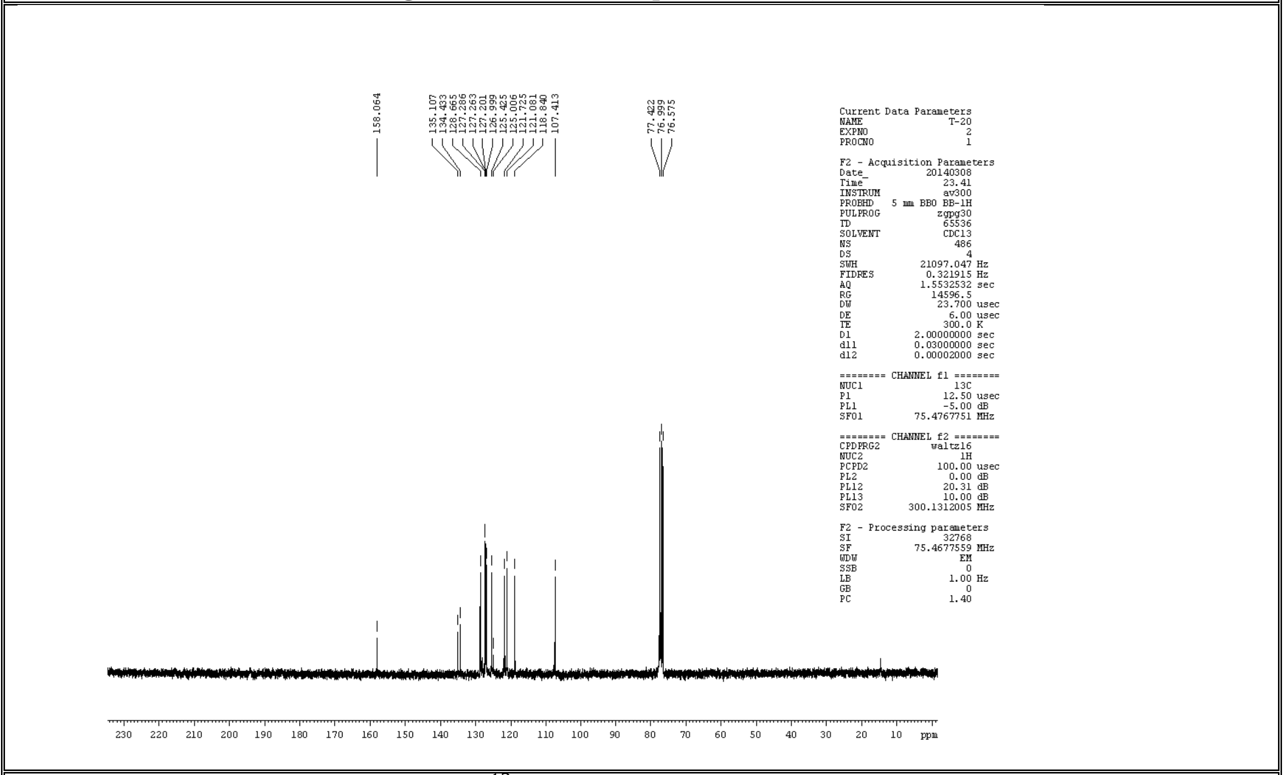

Figure 74 ^{13}C NMR Spectrum of **3r** ($CDCl_3$)

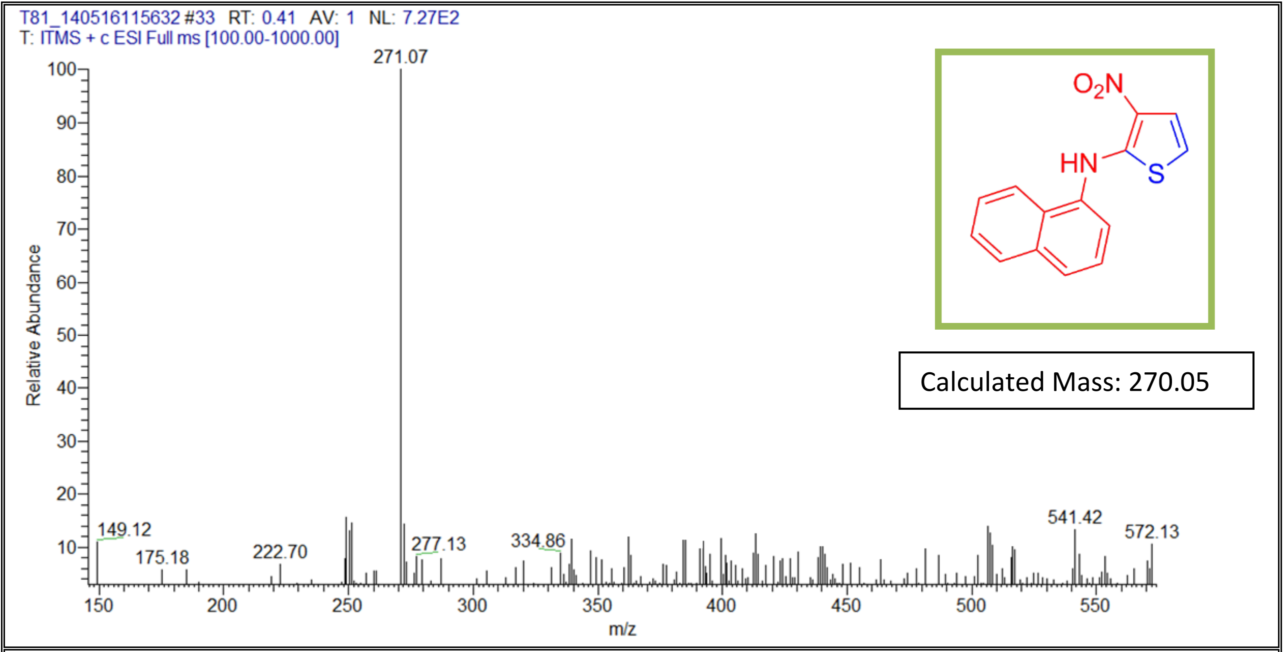

Figure 75 ESI mass spectrum of Spectrum of **3r**

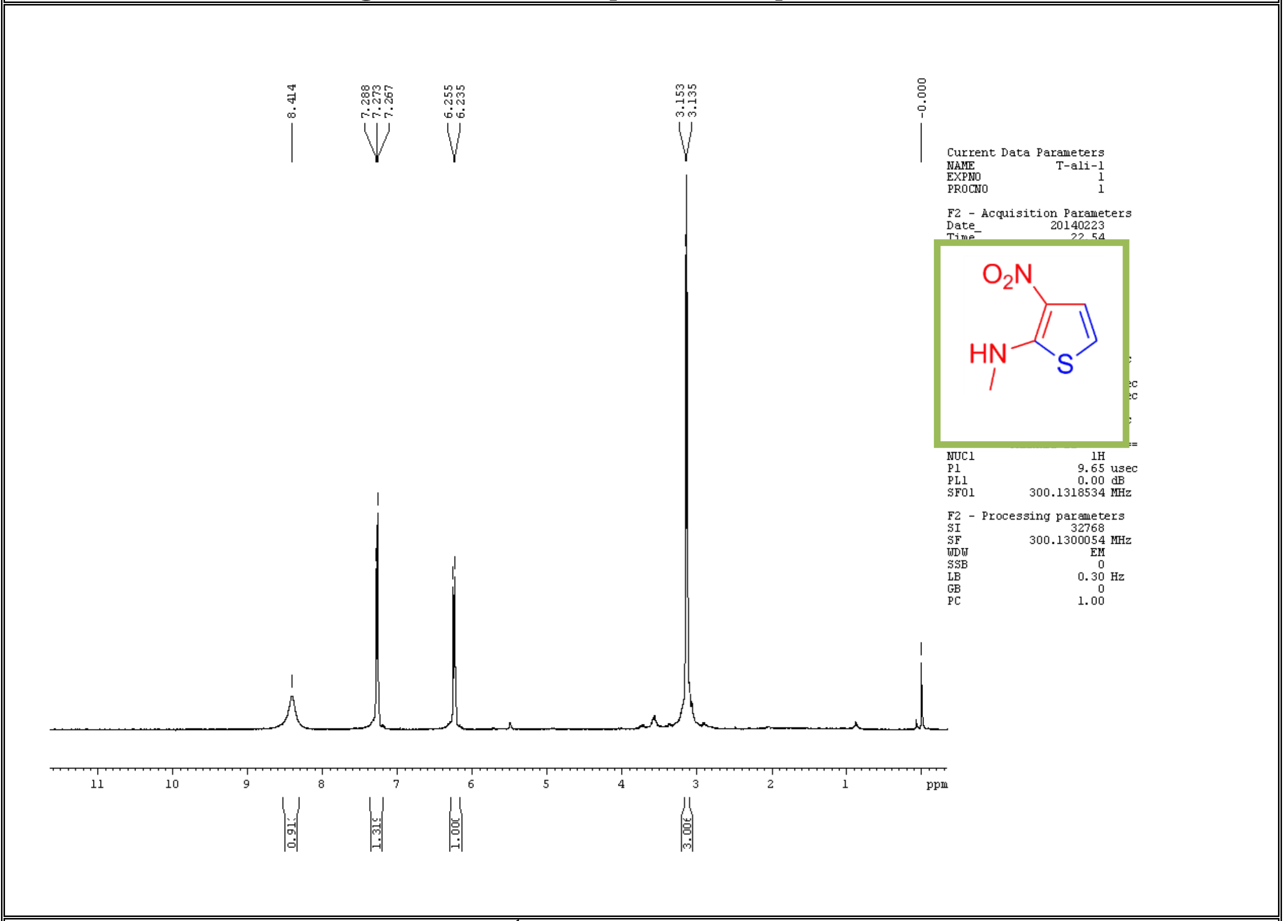

Figure 76 ^{1}H NMR Spectrum **3s** ($CDCl_3$)

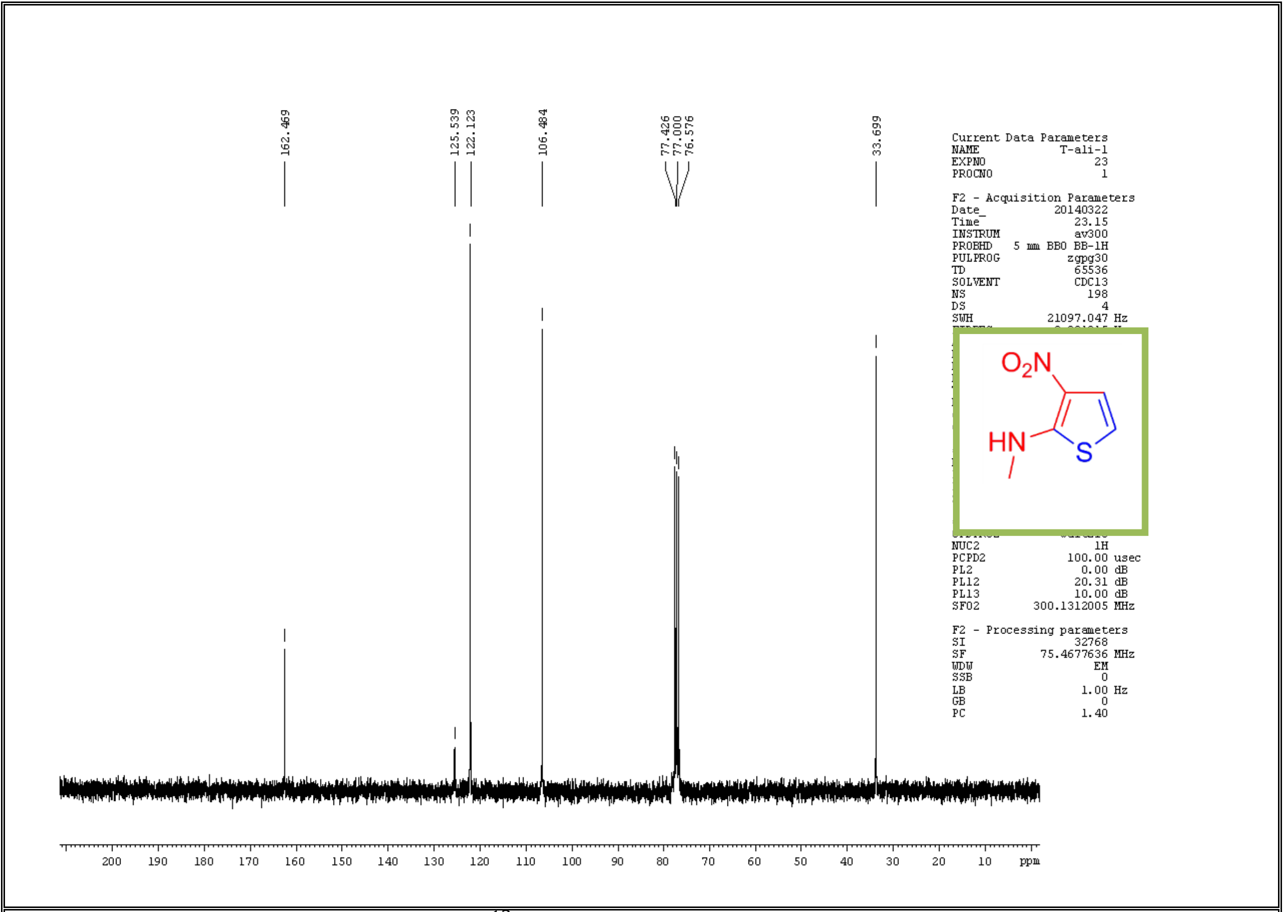

Figure 77 ^{13}C NMR Spectrum of **3s** ($CDCl_3$)

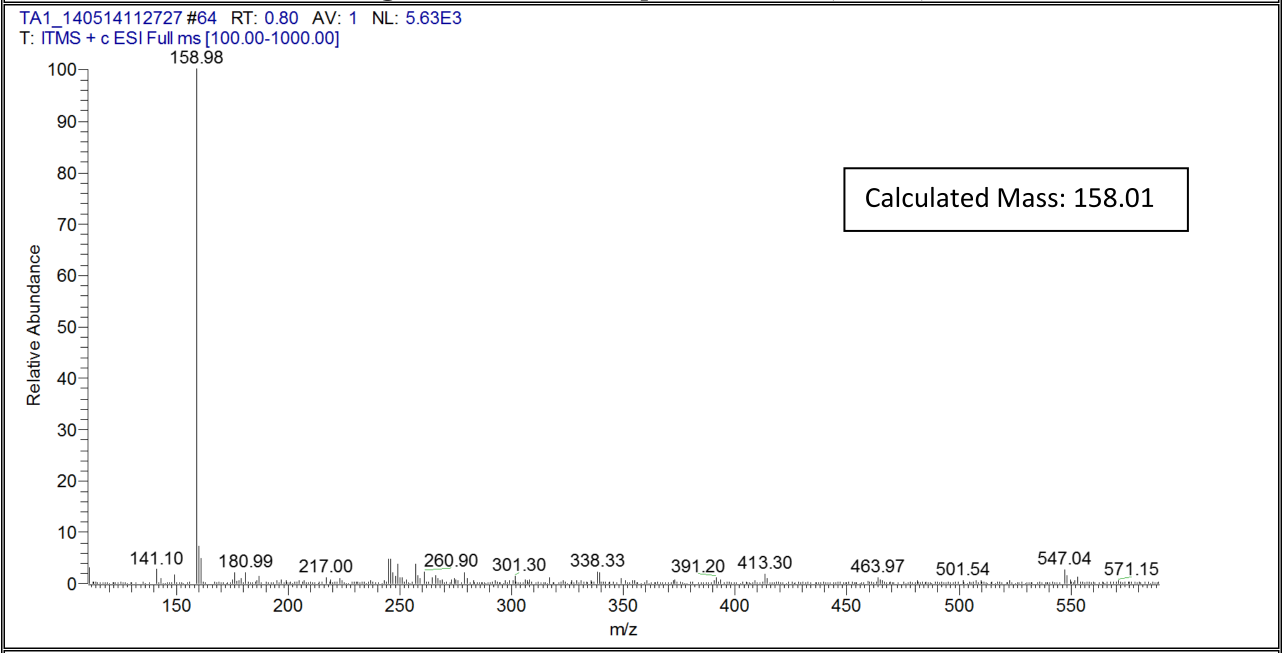

Figure 78 ESI mass spectrum of Spectrum of **3s**

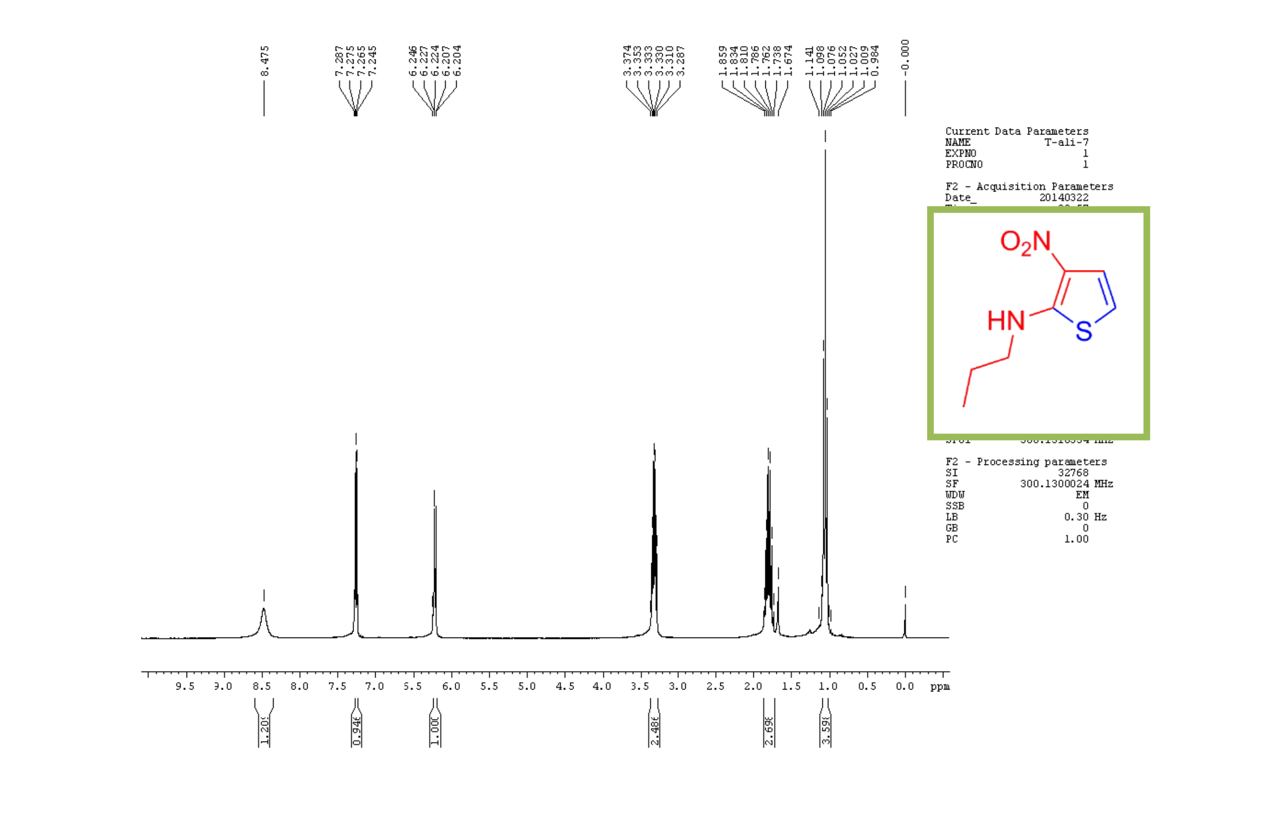

Figure 79 ^{1}H NMR Spectrum **3t** ($CDCl_3$)

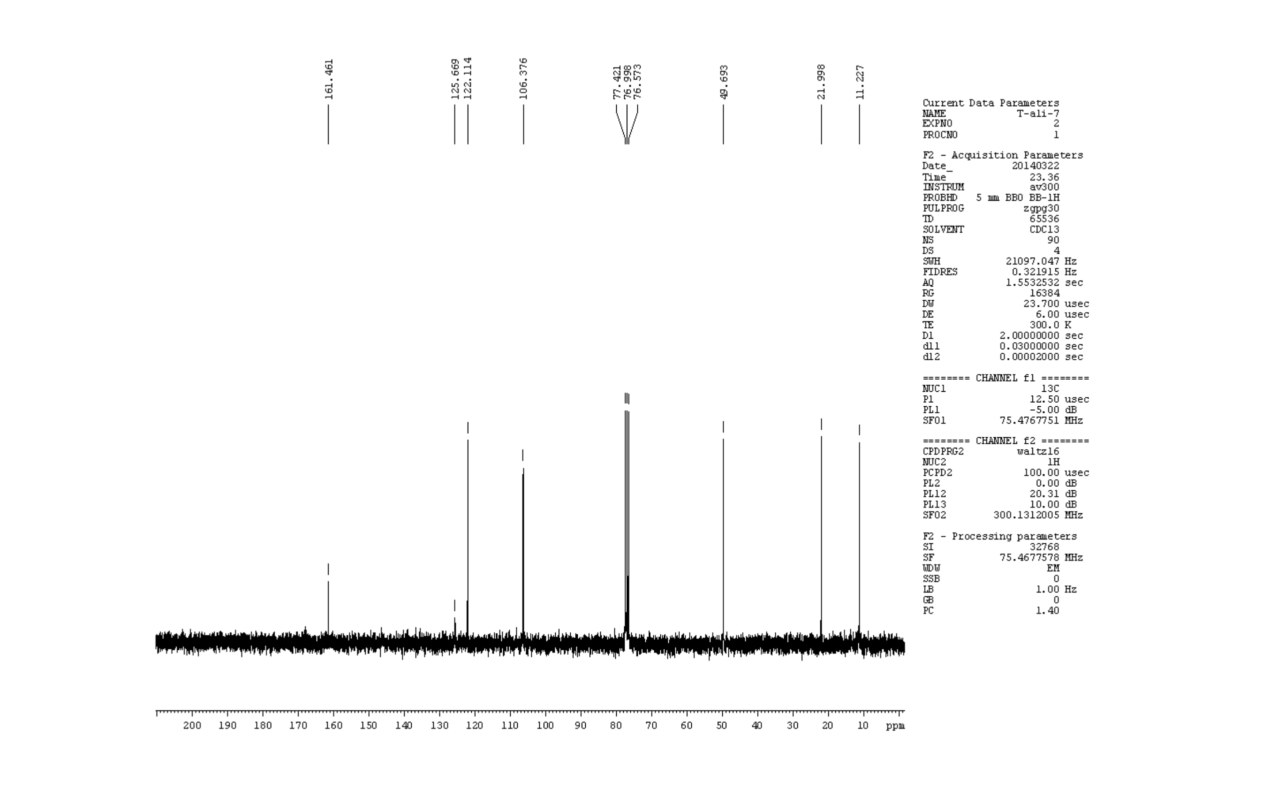

Figure 80 ^{13}C NMR Spectrum of **3t** ($CDCl_3$)

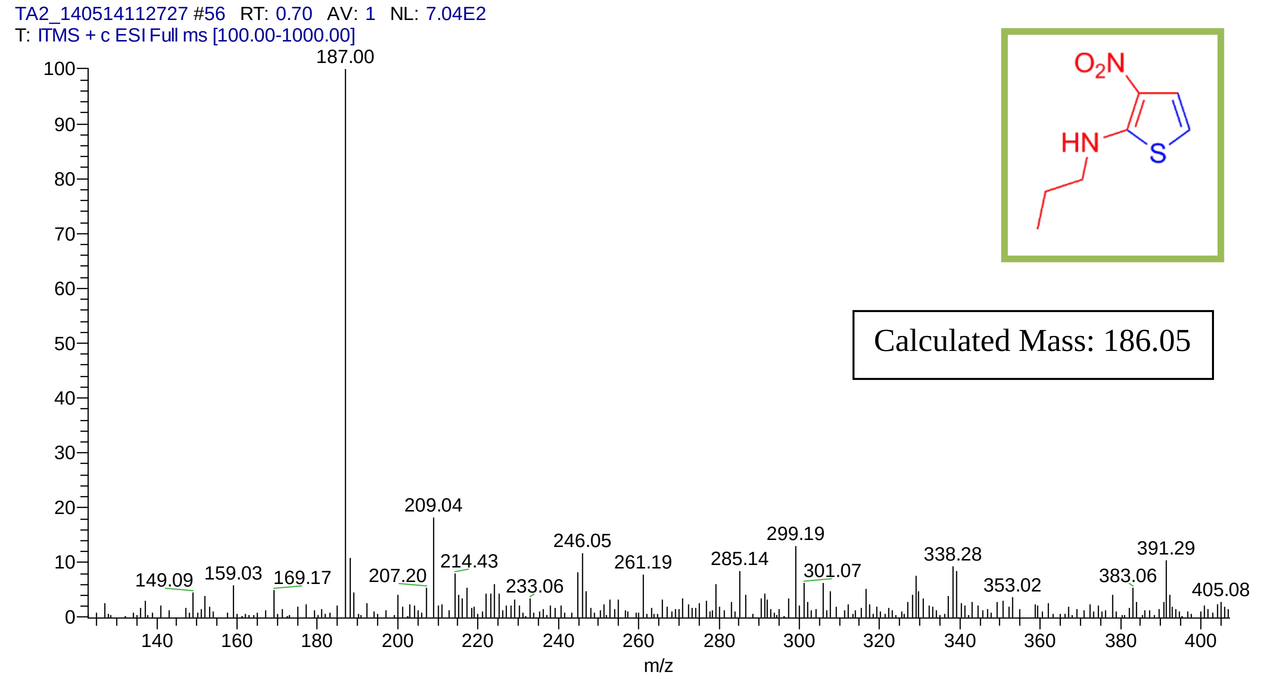

Figure 81 ESI mass spectrum of Spectrum of **3t**

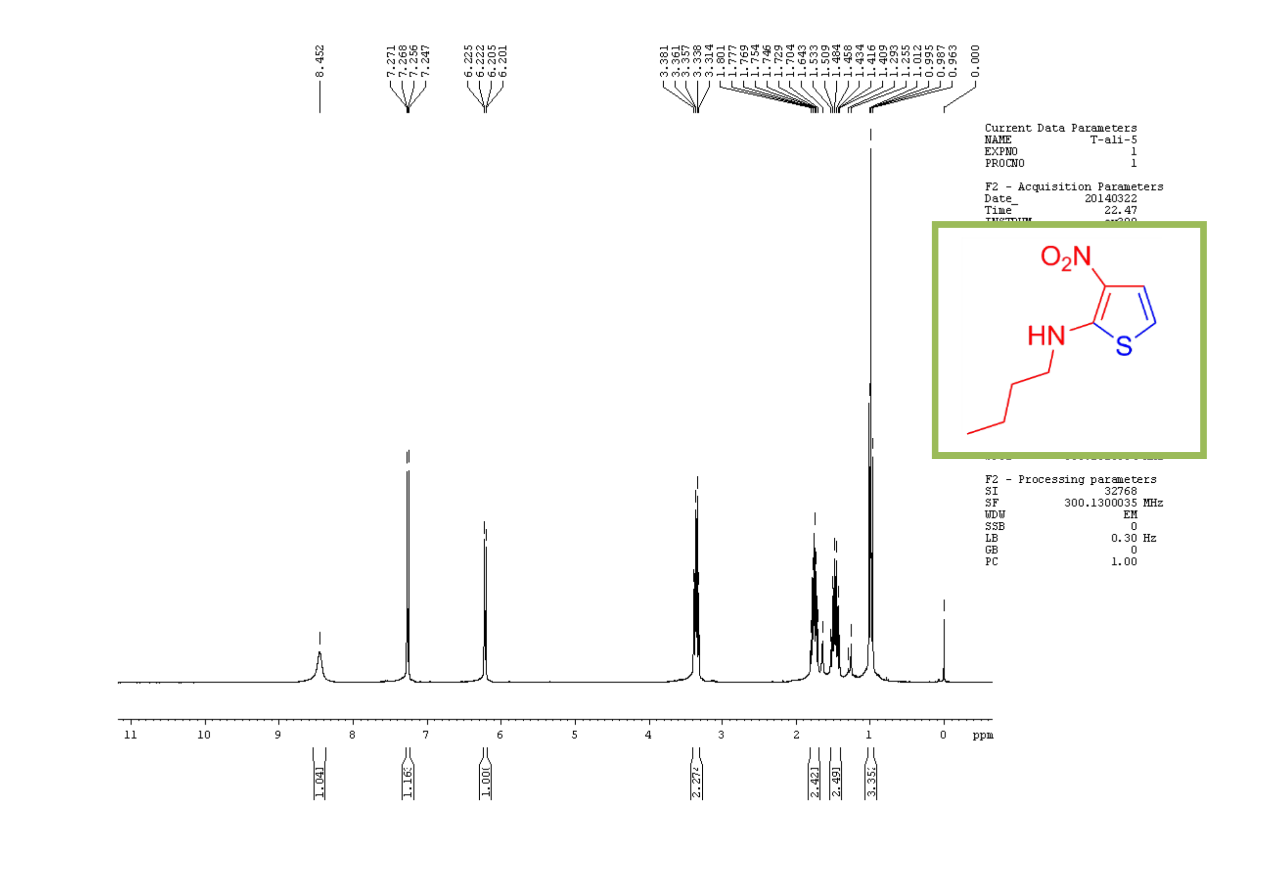

Figure 82 ^{1}H NMR Spectrum **3u** ($CDCl_3$)

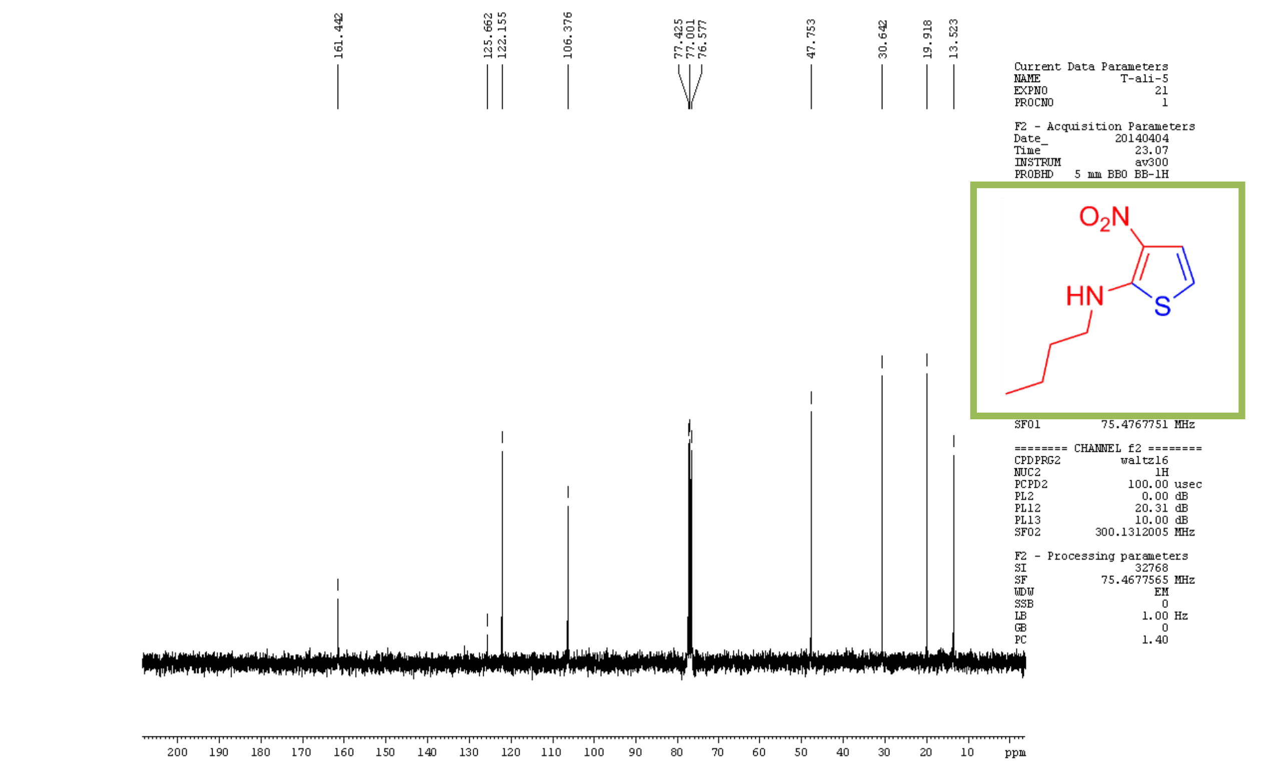

Figure 83 ^{13}C NMR Spectrum of **3u** ($CDCl_3$)

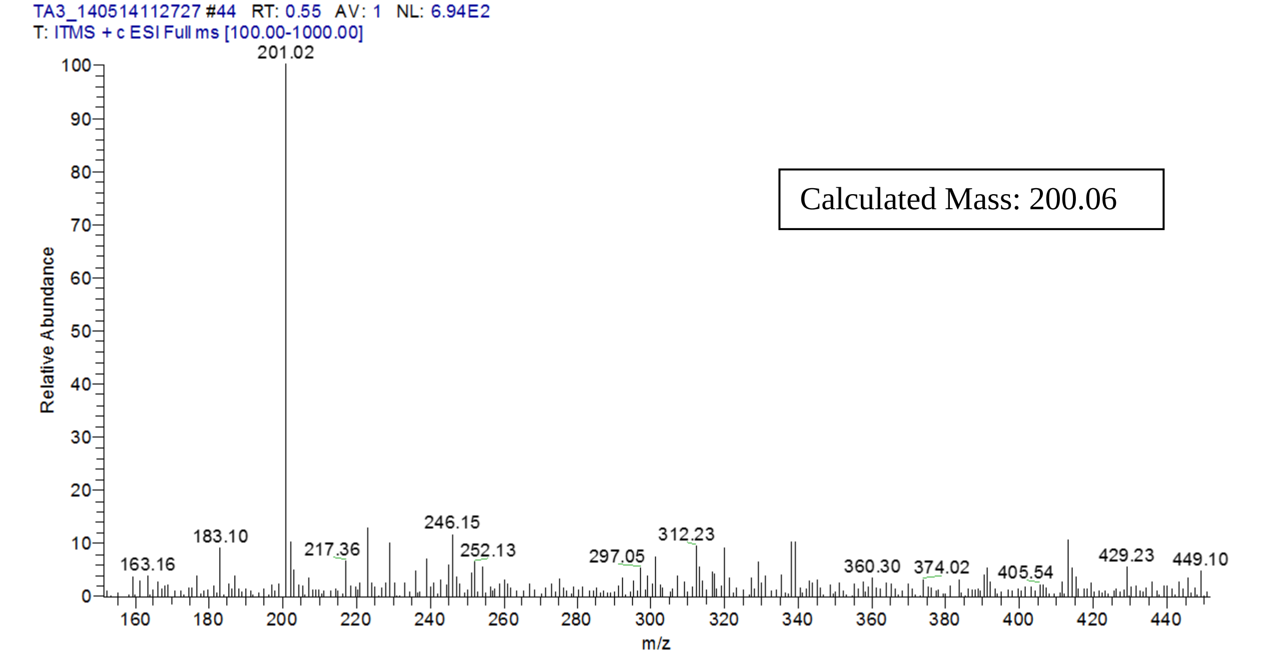

Figure 84 ESI mass spectrum of Spectrum of **3u**

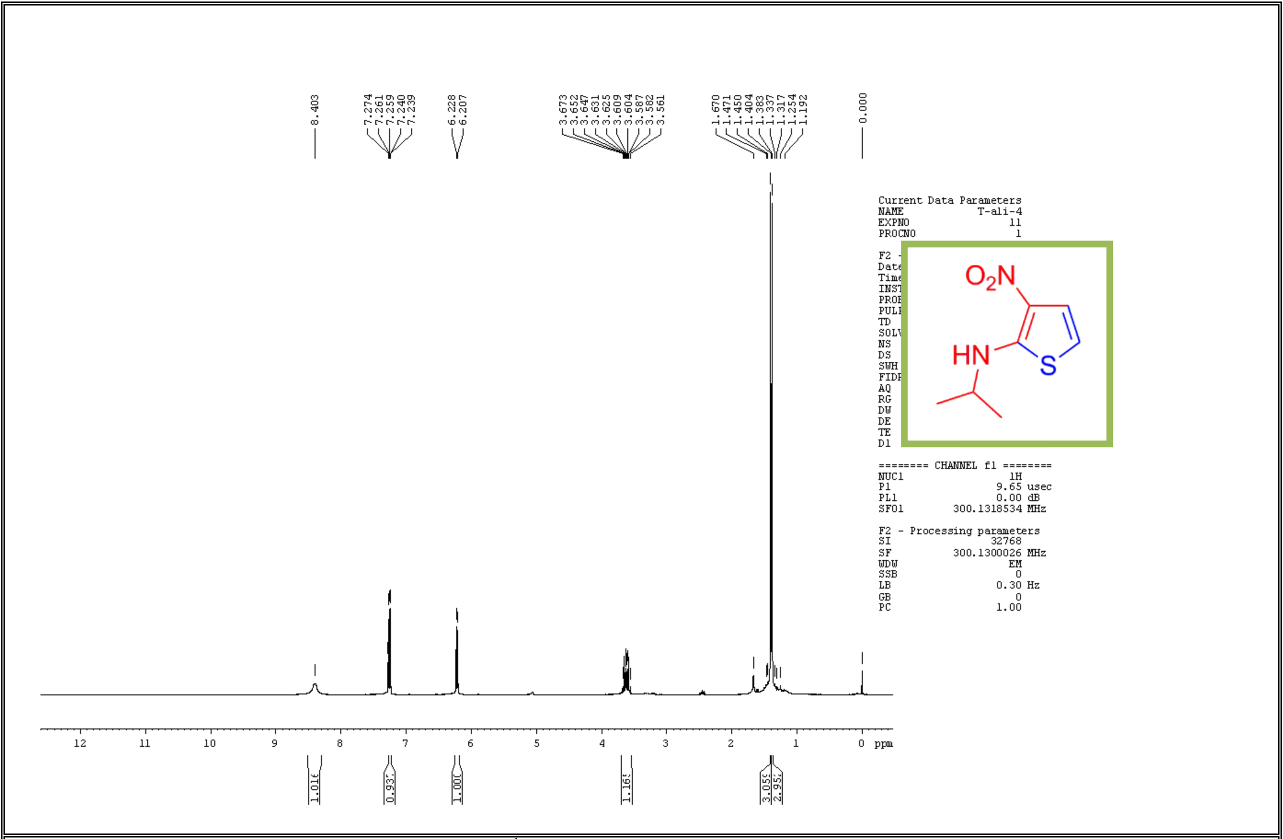

Figure 85 ^{1}H NMR Spectrum **3v** ($CDCl_3$)

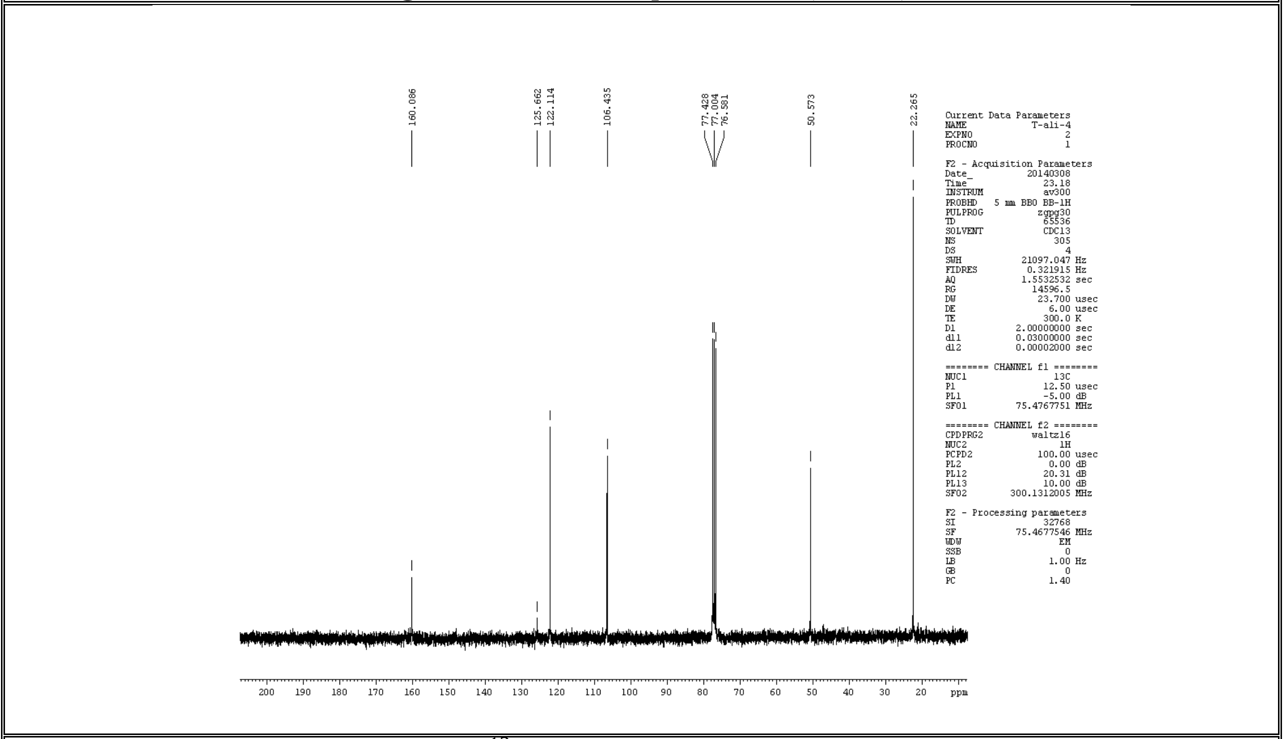

Figure 86 ^{13}C NMR Spectrum of **3v** ($CDCl_3$)

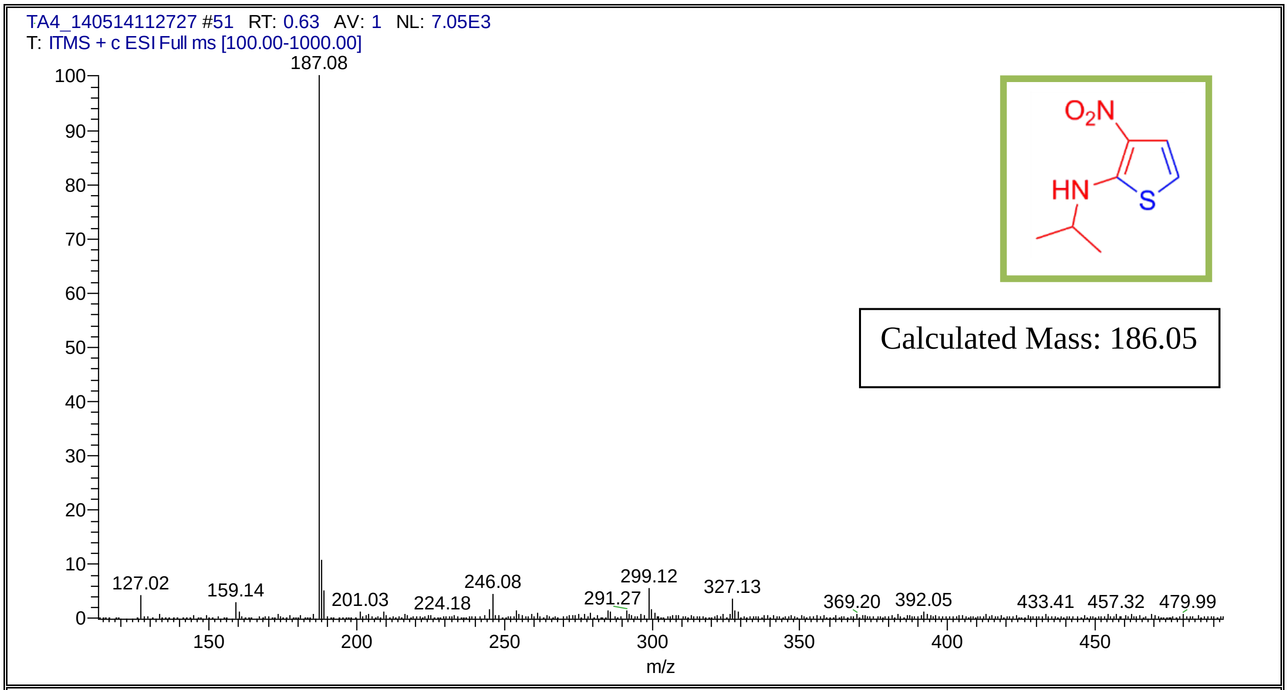

Figure 87 ESI mass spectrum of Spectrum of **3v**

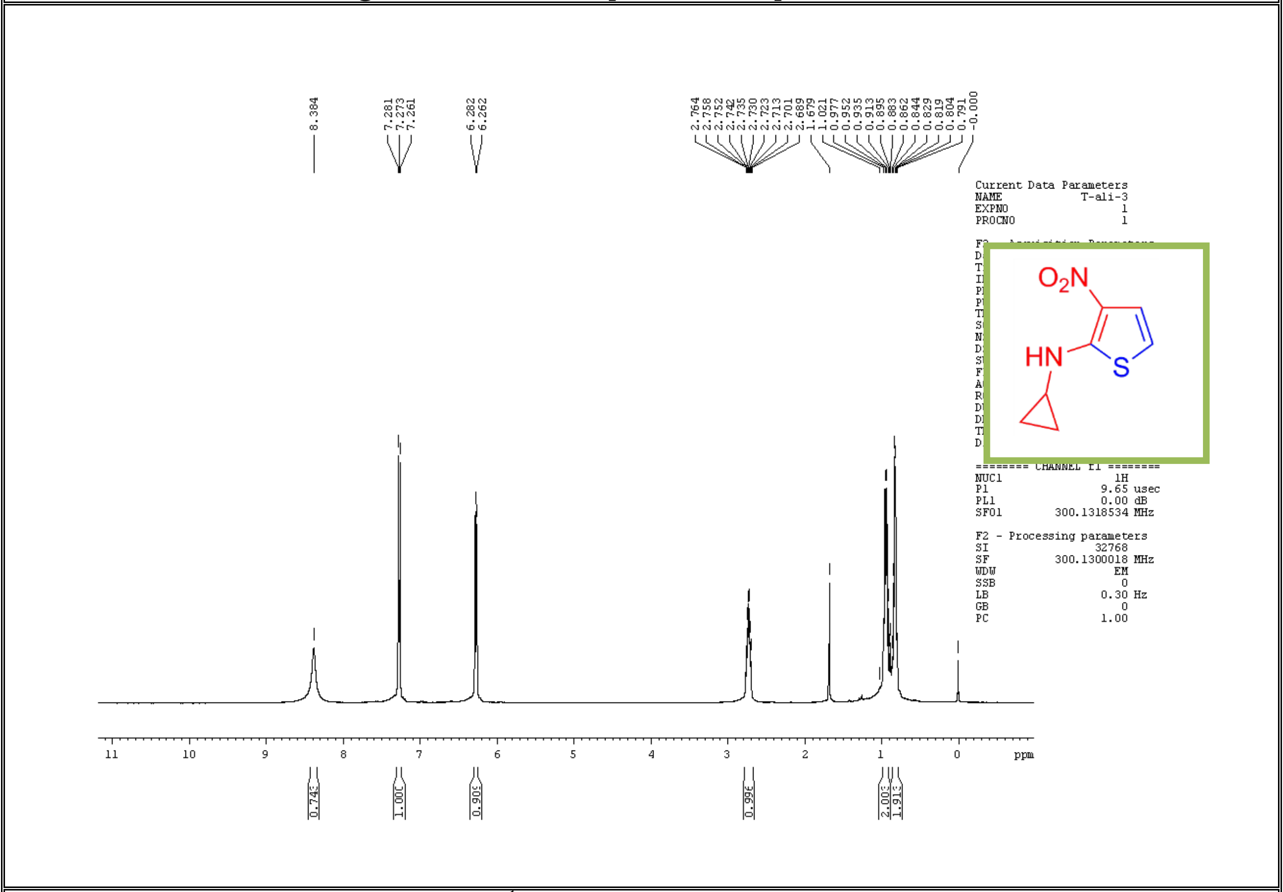

Figure 88 ^{1}H NMR Spectrum **3w** ($CDCl_3$)

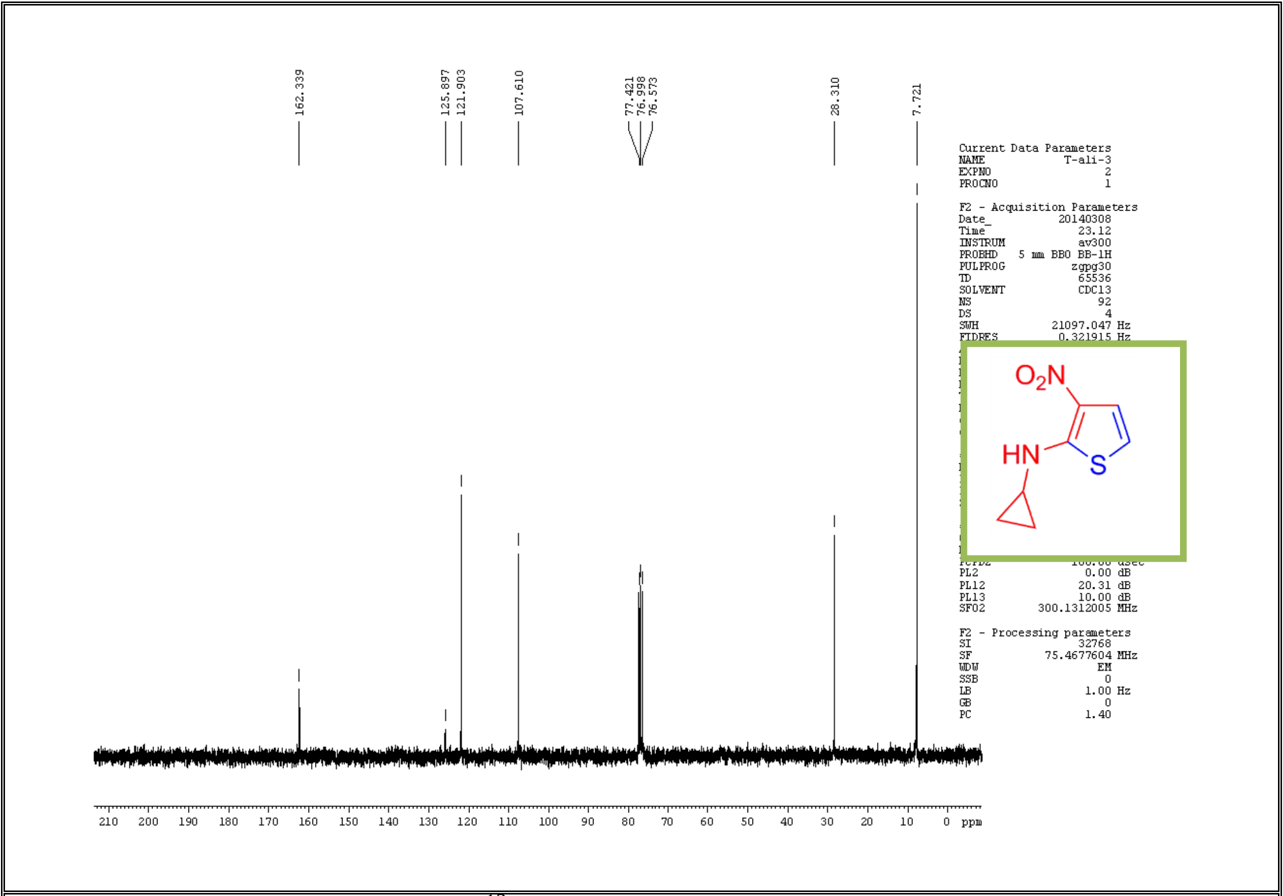

Figure 89 ^{13}C NMR Spectrum of **3w** ($CDCl_3$)

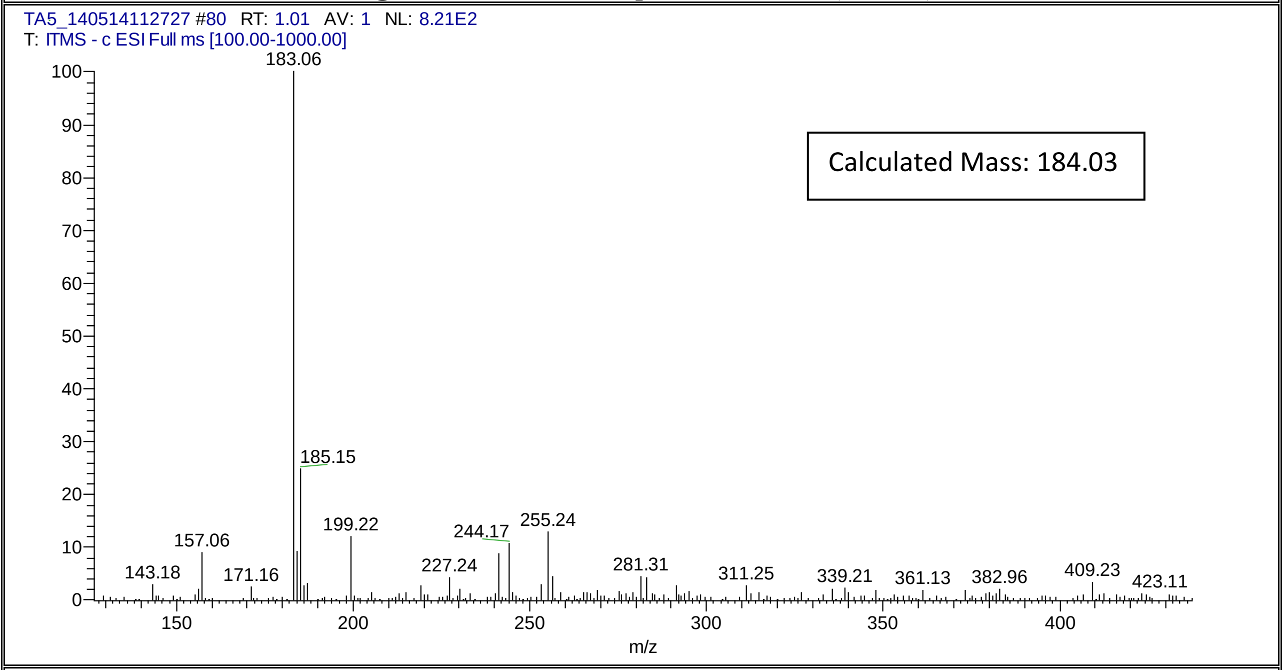

Figure 90 ESI mass spectrum of Spectrum of **3w**

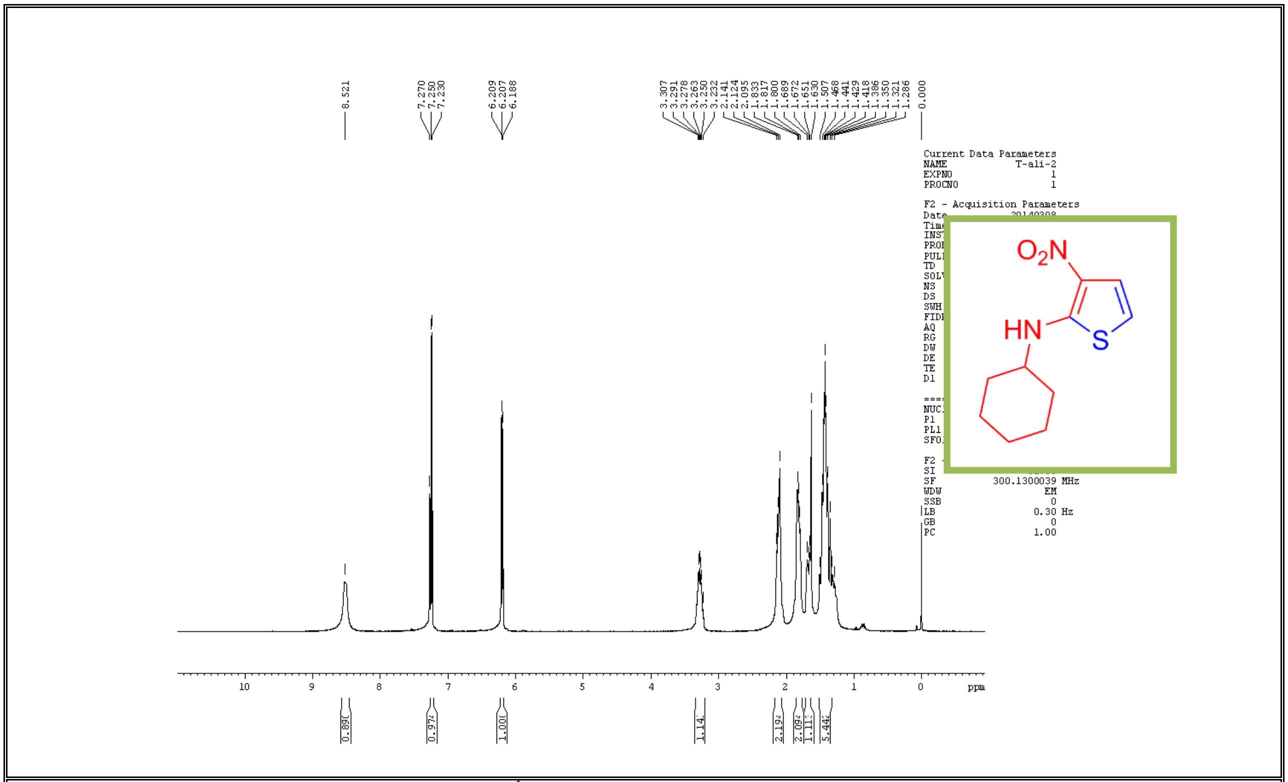

Figure 91 ^{1}H NMR Spectrum **3x** ($CDCl_3$)

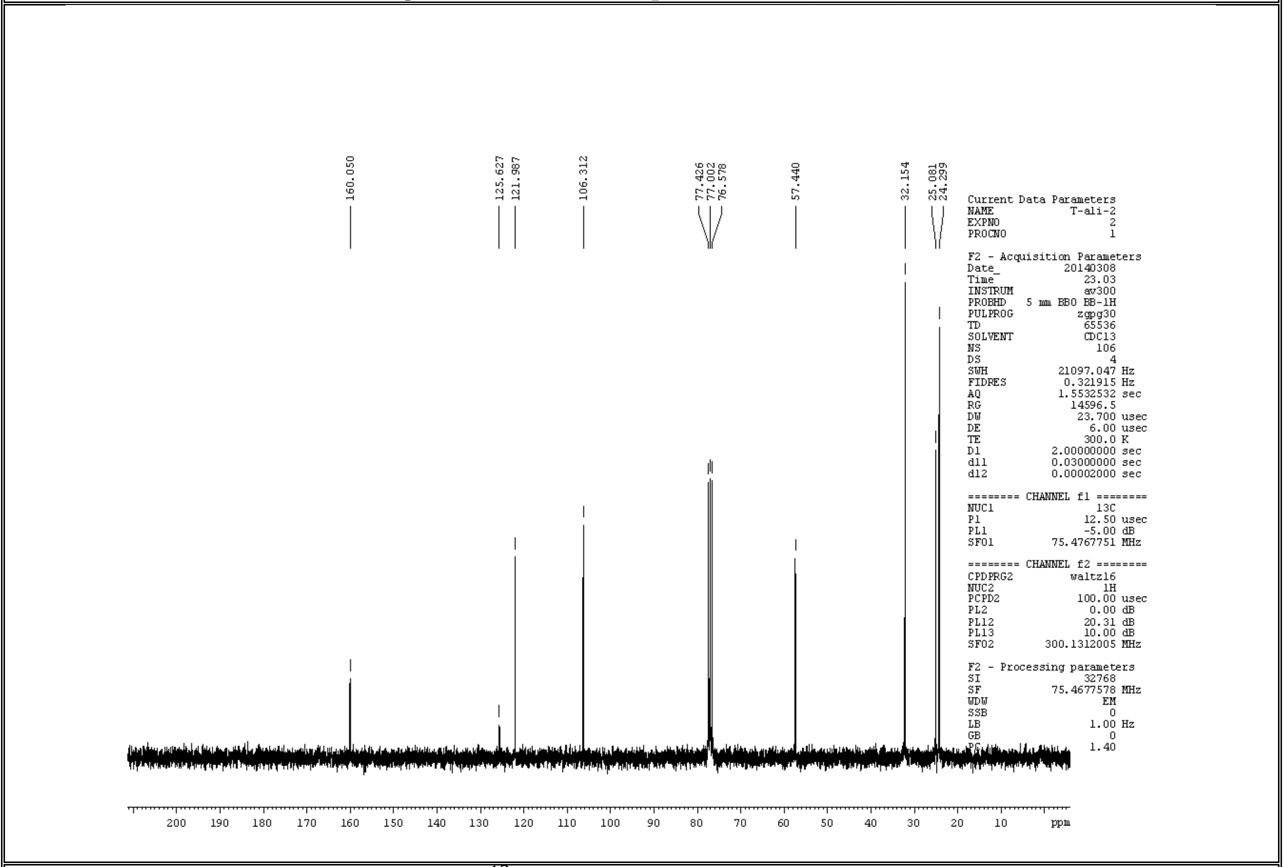

Figure 92 ^{13}C NMR Spectrum of **3x** ($CDCl_3$)

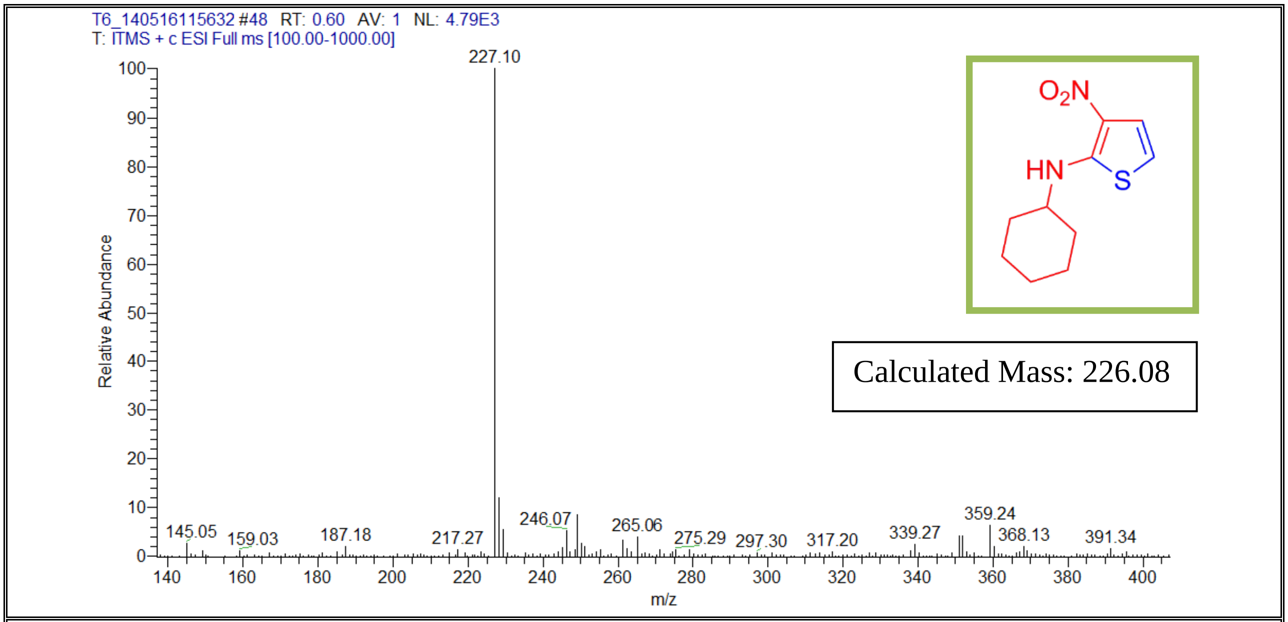

Figure 93 ESI mass spectrum of Spectrum of **3x**

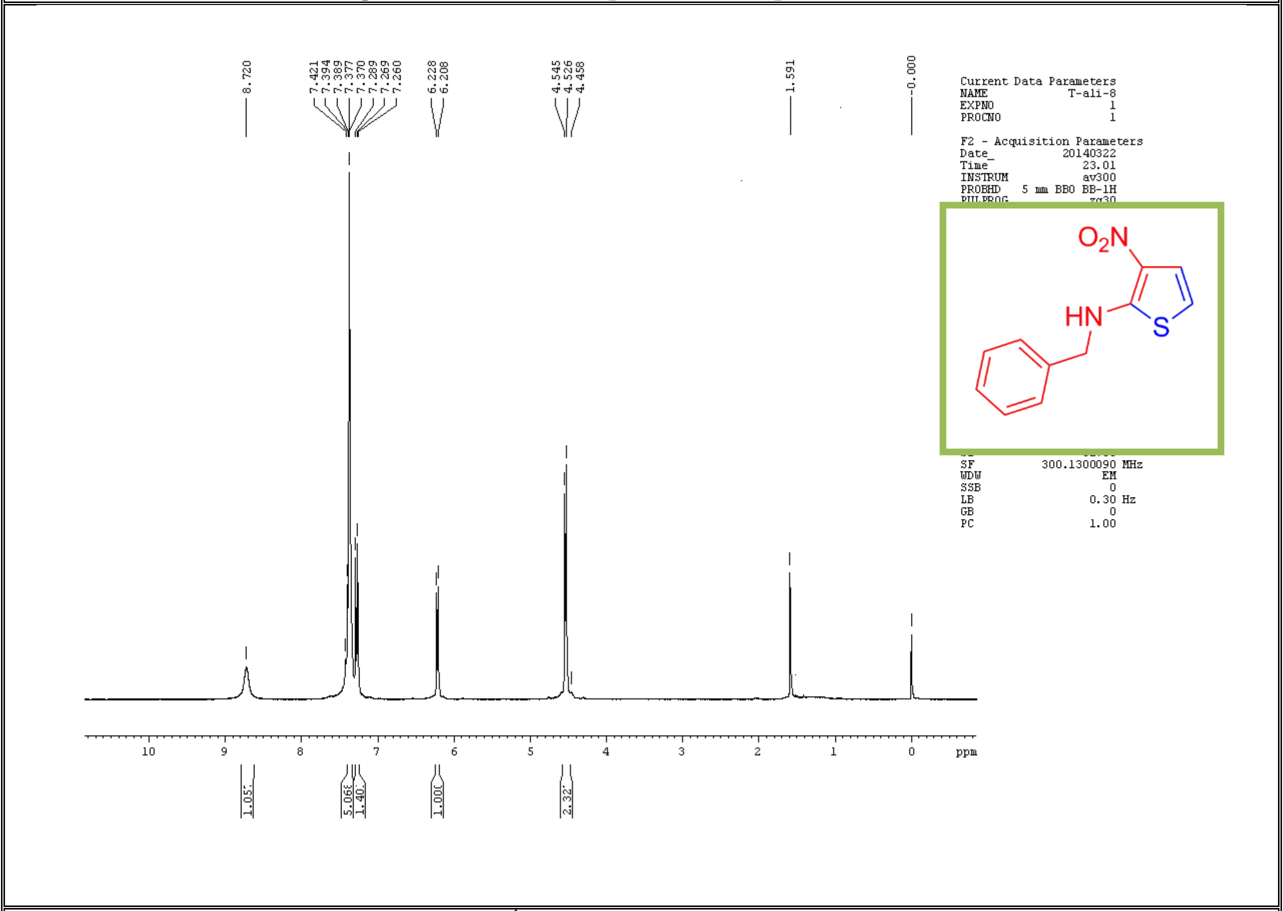

Figure 94 ^{1}H NMR Spectrum **3y** ($CDCl_3$)

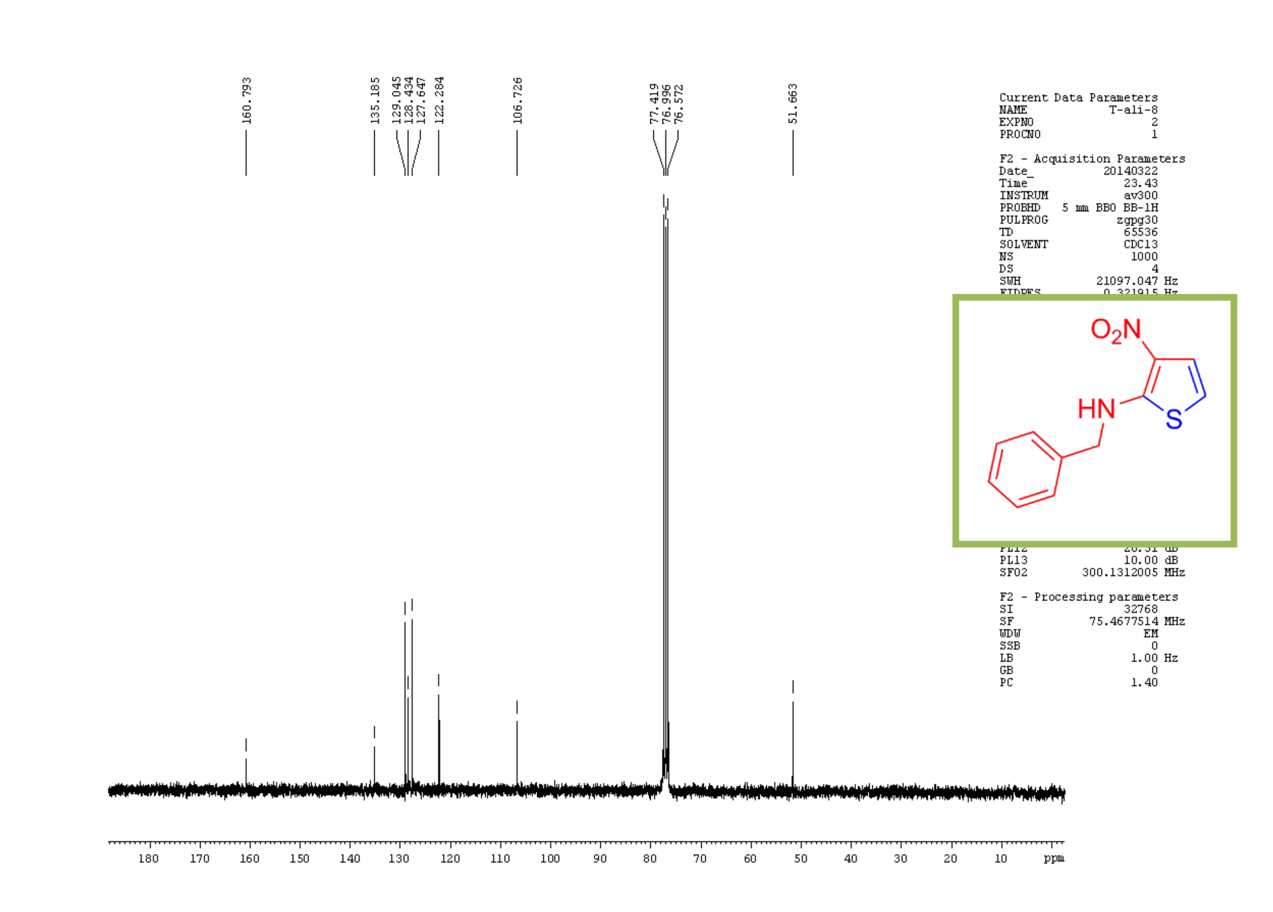

Figure 95 ^{13}C NMR Spectrum of **3y** ($CDCl_3$)

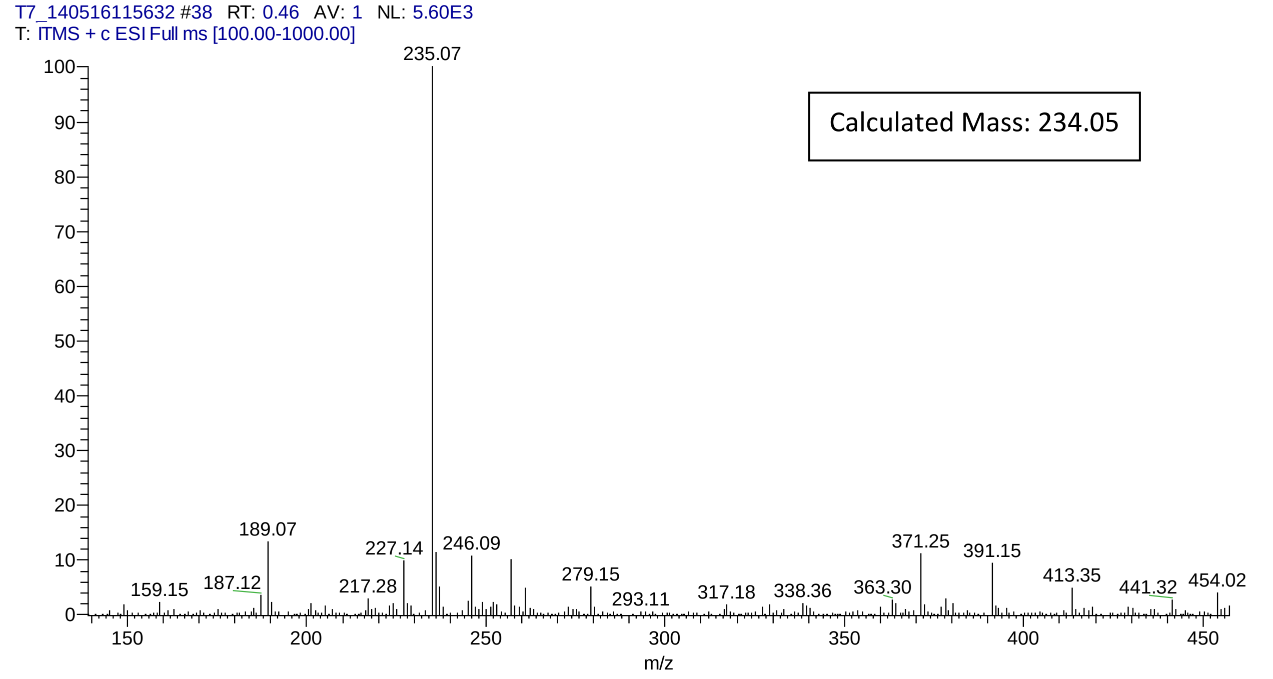

Figure 96 ESI mass spectrum of Spectrum of **3y**

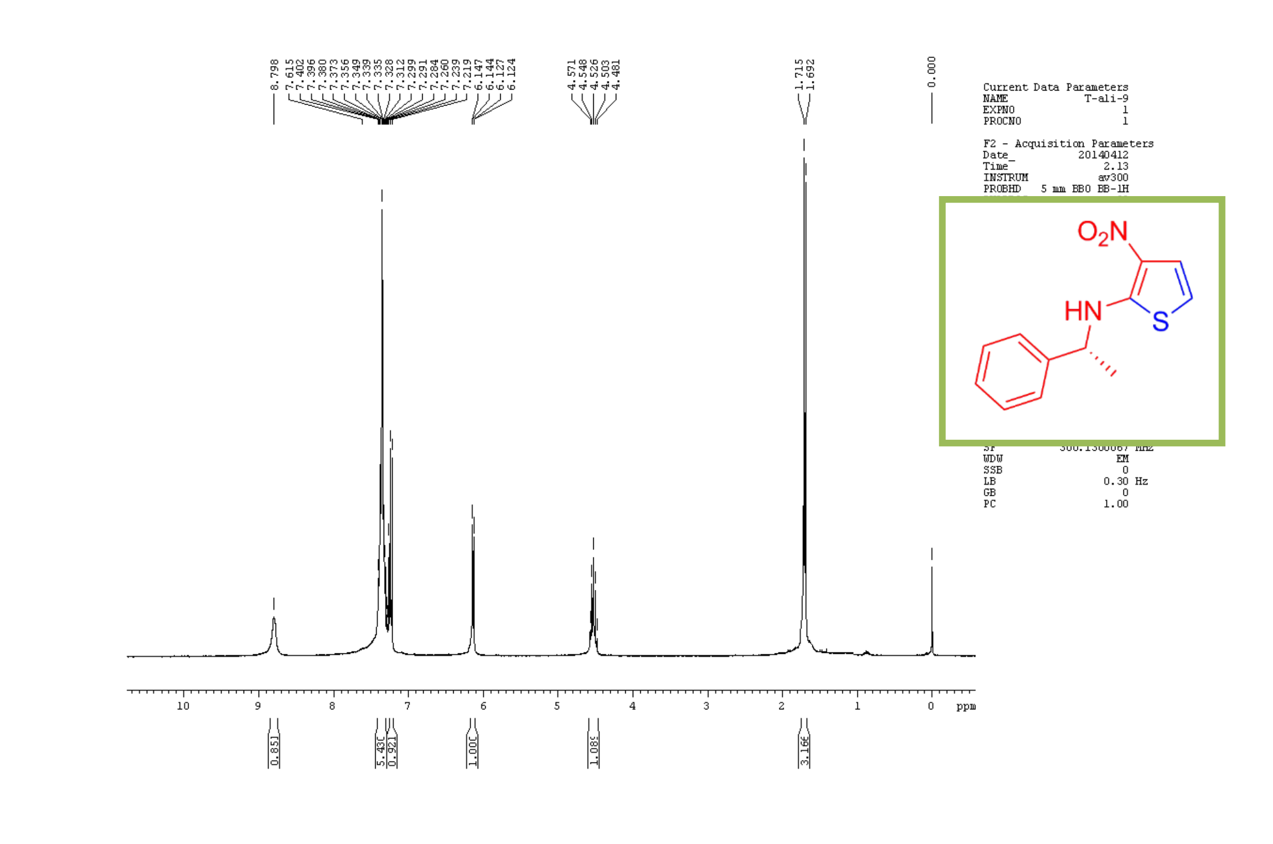

Figure 97 ^{1}H NMR Spectrum **3z** ($CDCl_3$)

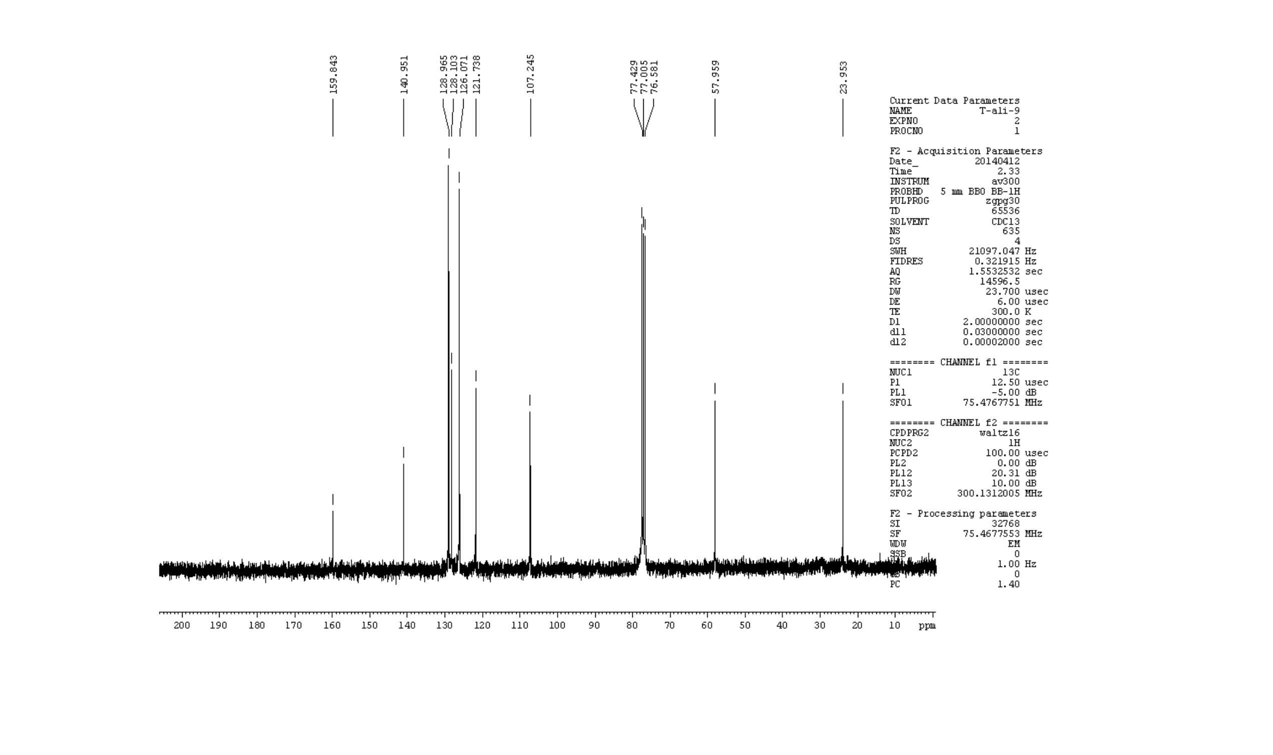

Figure 98 ^{13}C NMR Spectrum of **3z** ($CDCl_3$)

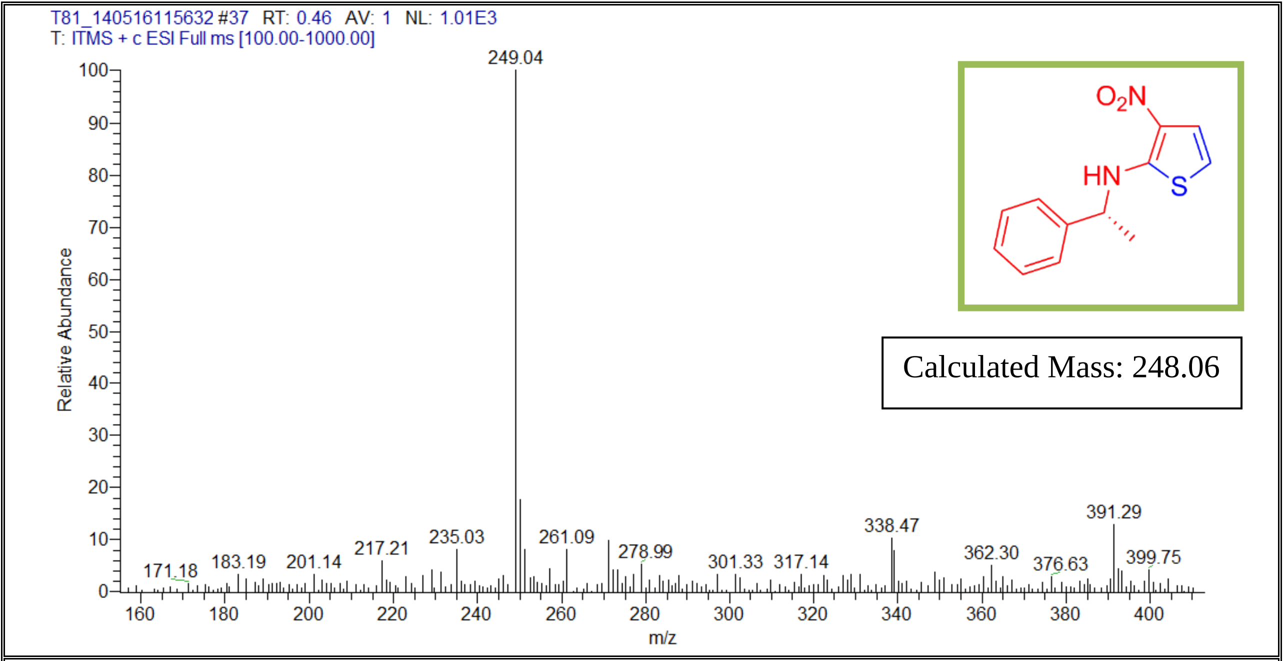

Figure 99 ESI mass spectrum of Spectrum of **3z**

References

1. Venkatesh, C.; Singh, B.; Mahata, P. K.; Ila, H.; Junjappa, H. *Org. Lett.* **2005,** *7,* 2169–2172. doi:10.1021/ol0505095
2. Buchholz, M.; Hamann, A.; Aust, S.; Brandt, W.; Bohme, L.; Hoffmann, T.; Schilling, S.; Demuth, H.-U.; Heiser, U. *J. Med. Chem.* **2009,** *52,* 7069–7080. doi:10.1021/jm900969p
